# The genomic timeline of cichlid fish diversification across continents

**DOI:** 10.1038/s41467-020-17827-9

**Published:** 2020-11-18

**Authors:** Michael Matschiner, Astrid Böhne, Fabrizia Ronco, Walter Salzburger

**Affiliations:** 1 Zoological Institute, University of Basel, Basel, Switzerland; 2 Department of Palaeontology and Museum, University of Zurich, Zurich, Switzerland; 3 Centre for Ecological and Evolutionary Synthesis (CEES), Department of Biosciences, University of Oslo, Oslo, Norway; 4 Center for Molecular Biodiversity Research (ZMB), Zoological Research Museum Alexander Koenig, Bonn, Germany

**Keywords:** Phylogenetics, Ichthyology

## Abstract

Cichlid fishes are celebrated for their vast taxonomic, phenotypic, and ecological diversity; however, a central aspect of their evolution — the timeline of their diversification — remains contentious. Here, we generate draft genome assemblies of 14 species representing the global cichlid diversity and integrate these into a new phylogenomic hypothesis of cichlid and teleost evolution that we time-calibrate with 58 re-evaluated fossil constraints and a new Bayesian model accounting for fossil-assignment uncertainty. Our results support cichlid diversification long after the breakup of the supercontinent Gondwana and lay the foundation for precise temporal reconstructions of the exceptional continental cichlid adaptive radiations.

## Abstract (web summary)

Cichlids are a model for adaptive radiation, but the timing of their diversification is debated. Here the authors assemble 14 cichlid genomes, introduce a Bayesian approach to account for fossil-assignment uncertainty, and present a dated phylogenomic hypothesis of cichlid and teleost evolution.

## Introduction

**Fig. 1:**
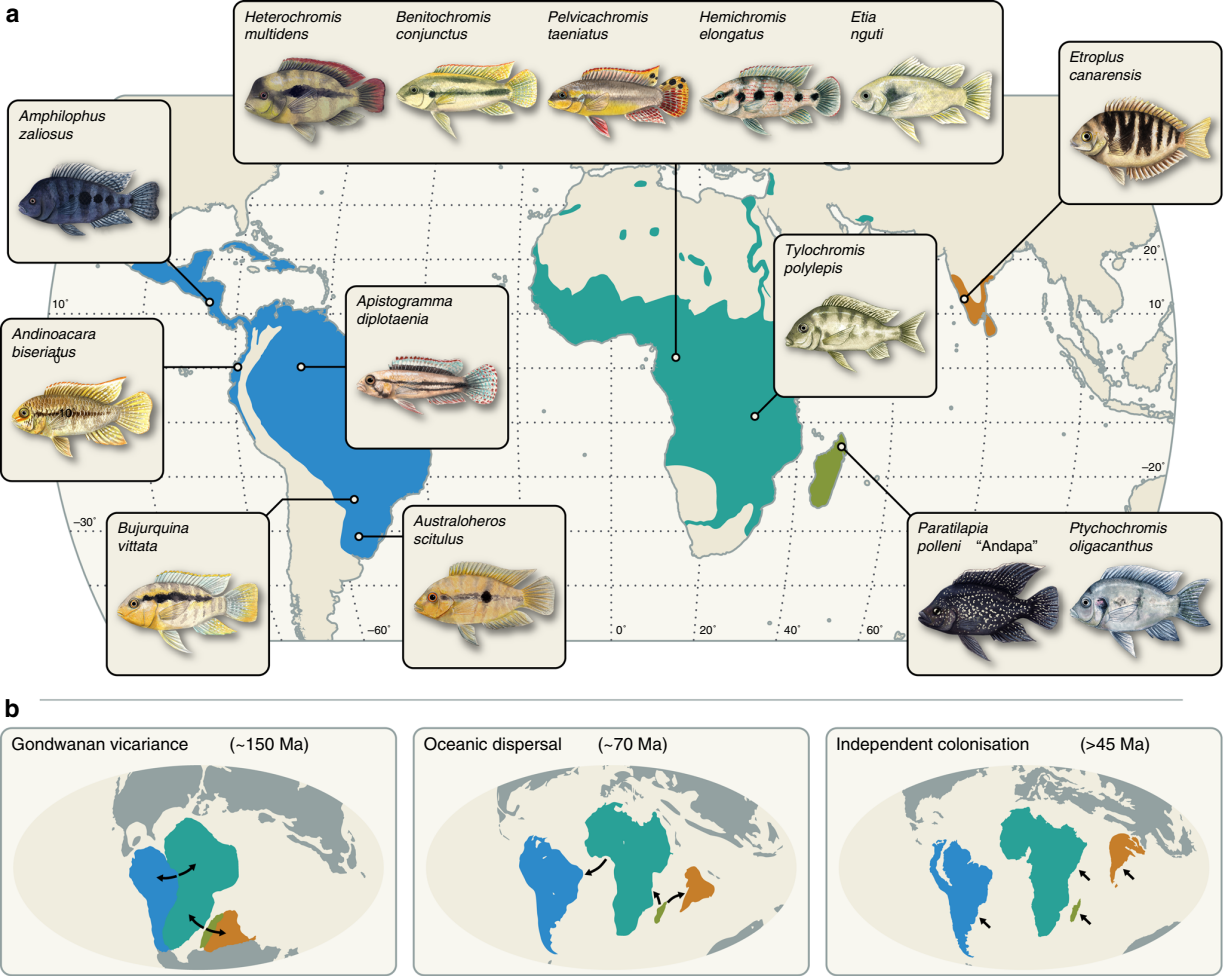
Global distribution of cichlid fishes and diversification scenarios. **a** Present-day distribution of cichlid fishes in the Americas (subfamily Cichlinae; blue), Africa, and the Levant (Pseudocrenilabrinae; cyan), Madagascar (Ptychochrominae; green), and the Indian subcontinent (Etroplinae; orange). The drawings illustrate the 14 cichlid species used for whole-genome sequencing. Their approximate geographic origins are indicated. **b** Three hypotheses for the phylogeographic history of cichlid fishes. According to the “Gondwanan vicariance” hypothesis, cichlids lived on the Gondwanan landmasses South America, Africa, Madagascar, and India before the separation of these landmasses and diverged as a result of this separation. This would require cichlids to be at least as old as the initial Gondwanan split, i.e., 150 million years. The “Oceanic dispersal” hypothesis posits that cichlids are younger than the separation of Gondwanan landmasses and hence reached their current distributions through long-distance oceanic dispersal. Some molecular studies suggest that this could have occurred around 70 Ma. An alternative hypothesis that is consistent with a young age of cichlids is the “Independent colonization” scenario, according to which cichlids on all four landmasses independently evolved from a common marine ancestor that has since either gone extinct or remained undiscovered. This must have occurred before 45 Ma because the presence of freshwater cichlids by that time is well documented in the fossil record.

Owing to their spectacular ecological and morphological diversity and species richness, cichlid fishes have become one of the most important model groups in evolutionary biology and adaptive radiation research^1,2^. Despite the great scientific attention that cichlids have received in the last decades, a key aspect of their evolution—the timeline of their diversification and spread to Africa, the Americas, Madagascar, and India—remains controversial^3^. Depending on the study, available estimates for the age of the family Cichlidae range from 45 to 160 million years (Myr) and the divergence of the American and African subfamilies (which together include ~99% of cichlid species^2^) has been estimated as recently as 26 million years ago (Ma)^4^ or as early as 147 Ma^5^. The different age estimates imply contrasting scenarios for the spread of cichlids across continents: although the oldest estimates are compatible with an ancestral cichlid lineage that lived in freshwaters of the former supercontinent Gondwana and diverged by vicariance with its tectonic breakup between 150 and 85 Ma^3^, all younger timelines require either long-distance oceanic dispersal events or multiple independent transitions to freshwater from an unknown common marine ancestor (Fig. 1). Because of the requirement of salt-water tolerance or even a marine lifestyle for ancestral lineages of a clade that is confined almost exclusively to freshwater today, the two alternatives to vicariance may appear improbable. On the other hand, salinity tolerance has been observed in some of the most divergent extant cichlid species (reviewed in ref. ^3^) and the marine-living convict blenny (*Pholidichthys*) has been identified as the closest living relative to cichlids^6^, suggesting that adaptations to marine levels of salinity may have been more common in the early evolution of cichlids.

The contrasting estimates regarding the timeline of cichlid evolution are due—at least in part—to the use of small phylogenetic datasets dominated by mitochondrial sequences^7–9^ and to the application of strategies for time calibration that rely exclusively on the cichlid fossil record, without taking into consideration the larger context of teleost evolution, into which the cichlid timeline must be placed^5,10,11^. Even if these two issues are addressed, age estimates are still heavily influenced by the often ambiguous assignment of calibration fossils to taxonomic clades, as highlighted in recent studies^12–14^. For example, when analyzing the same genomic dataset twice with two different fossils that are both currently discussed as potential first records of the teleost order Tetraodontiformes (†*Plectocretacicus clarae* with an age of 100.3–98.0 Ma and †*Cretatriacanthus guidottii* with an age of 89.8–83.0 Ma; Supplementary Note 1), Musilova et al.^14^ obtained two timelines that differed by more than 10 Myr for the age of acanthomorph fishes, a group that comprises roughly a third of all vertebrate species. This implies that conclusions drawn even from large phylogenomic analyses may only be valid under certain assumptions for the positions of key fossils. To account for ambiguous fossil positions, methods have been developed that either infer a fossil’s position during the molecular-clock analysis from scored morphological characters^15^ or allow multiple positions for one and the same fossil on a fixed tree topology^16^. However, neither of these methods is suitable for the examination of highly diverse groups of species^17^ when morphological character matrices are not available and the tree topology is not known a priori.

We here address all three issues that have so far prevented reliable age estimates for cichlid fishes: we (i) provide whole-genome sequencing data for representatives of the global diversity of cichlids, (ii) embed these species into a genome-based phylogeny of teleosts, and (iii) develop and apply a new method to account for uncertain fossil assignments. The resulting timeline based on 91 fish genomes and the fossil record supports the diversification of cichlid fishes long after the breakup of the Gondwanan supercontinent.

## Results

### Phylogenomic inference of the species-tree topology

We generated draft genome assemblies based on low-coverage Illumina sequencing (7–23×) for 14 cichlid species including 1 species from India, 2 species from Madagascar, 5 from the Americas, and 6 from Africa (Fig. 1 and Supplementary Tables 1 and 2). We then used these whole-genome assemblies, together with a targeted assembly of candidate genes (Supplementary Tables 3–5), to identify 646 single-copy markers with a total alignment length of 127,638 bp for phylogenomic analyses (Supplementary Tables 6–8). Based on these markers, we inferred the species tree for a set of 90 teleost species, including 18 cichlid species, and 1 non-teleost outgroup (Supplementary Figs. 1–4).

Although species-tree estimates produced with the program BEAST 2^18^ from concatenated alignments (Supplementary Figs. 1 and 2) agreed well with the current understanding of teleost taxonomy^12,13,19^, a number of clades that have received unambiguous support from both morphological and molecular datasets (e.g., Acanthomorphata^14,19–22^) were not recovered in analyses with the program ASTRAL-III^23^ based on the multi-species coalescent model (Supplementary Figs. 3 and 4). We therefore consider concatenation as the more suitable approach for phylogenomic inference with our dataset. Given the long evolutionary time over which the species in our taxon set diversified (with branch lengths on the order of millions to hundreds of millions of years), the effect of incomplete lineage sorting is likely negligible and the proven statistical inconsistency of concatenation^24^ due to incomplete lineage sorting is unlikely to affect our conclusions^25^.

### Fossil-based time calibration

**Fig. 2:**
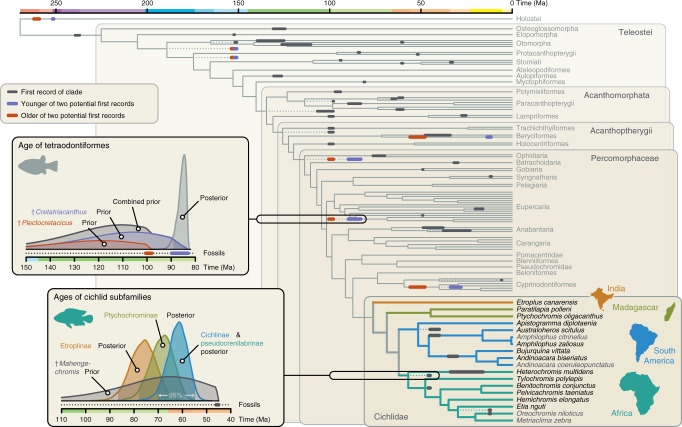
The genomic timeline of cichlid and teleost diversification. The 14 cichlid species with new genome sequences are highlighted in bold. Fossils used for time calibration are marked on branches according to the fossil’s age and its uncertainty. For seven clades, the first occurrence is ambiguous. In these cases, the younger and older of the two potential first records are marked in purple and red, respectively. One example of such a clade, Tetraodontiformes, is shown in the first inset. Either †*Cretatriacanthus guidottii* (89.8–83.0 Ma) or †*Plectocretacicus clarae* (100.3–98.0 Ma) represent the first record of this clade. Our new model for time calibration with ambiguous first records calculates separate CladeAge prior densities for both fossils (shown in purple and red) and forms a combined prior density (shown in dark gray) based on these. The resulting genomic age estimate (posterior; shown in light gray) indicates that Tetraodontiformes are younger than †*Plectocretacicus* and thus supports †*Cretatriacanthus* as the first record of the group. The CladeAge prior density for the oldest cichlid record, †*Mahengechromis*, and the age estimates for the four cichlid subfamilies are shown in the second inset. A version of this phylogeny with all tip labels is provided in Supplementary Fig. 6.

To account for ambiguity in fossil assignments, we extended the CladeAge approach for BEAST 2^9,18^ so that two fossils can now be specified as potential first records of a clade and weighed according to their relative credibilities. A prior density for the age of the clade is then calculated, taking into account both fossils and their relative credibilities simultaneously (Supplementary Fig. 5). We applied this extended CladeAge approach to time calibrate the teleost species tree with fossil calibrations for 51 clades, of which 7 clades had 2 ambiguous first records (Supplementary Figs. 6 and 7). In six of these seven ambiguous cases, we assigned equal weights to each of two potential first records, naively considering both equally likely to be the true first record of the clade (Supplementary Note 2). The exception to this were the two potential first records of Tetraodontiformes, †*P. clarae* and †*C. guidottii*, where we assigned twice the weight to the latter, because we considered it more likely to be the true first record of the clade based on its recent re-evaluation^12–14,26–28^ (Fig. 2).

Our divergence-time estimates are in agreement with the teleost and cichlid fossil records, and pinpoint the age of cichlids at 87.3 Ma (96.9–77.9 Ma; 95% highest posterior density interval), the divergence of the Indian subfamily Etroplinae at 76.2 Ma (86.6–66.3 Ma), the separation of the Malagassy subfamily Ptychochrominae at 68.7 Ma (78.0–59.6 Ma), and the divergence between American Cichlinae and African Pseudocrenilabrinae at 62.1 Ma (70.1–54.6 Ma) (Fig. 2), whereby the latter two divergences are close to the Cretaceous-Paleogene boundary, a time of global turmoil^29^. These estimates are robust to alternative assumptions for the fossilization process modeled with CladeAge and for the topology of the species tree, and are reduced when the cichlid fossil record is ignored (Table 1). By accounting for uncertainty in fossil assignment, our estimates are also able to resolve the dispute regarding the first record of Tetraodontiformes: as the estimated age of the order is younger than the older of the two potential first records (†*P. clarae*), our results reject the placement of this fossil within the order.

**Table 1:** Age estimates for selected clades, obtained with different settings and datasets.

Setting/Dataset	Holostei and Teleostei	Cichlidae	Cichlinae and Pseudocrenilabrinae
“permissive” gene set	269.6 (290.0–251.6)	76.2 (86.6–66.3)	62.1 (70.1–54.6)
“strict” gene set	269.2 (288.5–251.5)	76.9 (87.1–66.2)	62.2 (70.3–54.6)
MCMC sampling from prior	269.6 (290.9–251.5)	79.7 (94.9–63.9)	68.0 (81.3–55.1)
Without cichlid calibrations	268.9 (288.7–251.6)	61.2 (77.8–45.5)	38.7 (50.8–27.1)
Doubled net diversification rate	264.0 (277.5–251.4)	73.7 (83.6–64.2)	60.0 (66.9–53.3)
Halved net diversification rate	277.4 (306.7–251.9)	79.7 (90.8–69.4)	63.5 (72.5–55.0)
Doubled fossil sampling rate	263.5 (279.0–251.4)	73.7 (84.2–64.5)	59.9 (67.2–52.7)
Halved fossil sampling rate	277.9 (302.5–252.5)	81.3 (92.3–70.6)	65.3 (74.1–55.8)
(Osteoglossomorpha, Elopomorpha)	269.4 (289.5–251.5)	77.3 (88.1–67.9)	62.2 (70.3–54.9)
(Osteoglossomorpha, Clupeocephala)	269.2 (289.2–251.5)	76.7 (86.1–66.2)	62.0 (70.1–54.1)

Unless specified, the “strict” set of genes was used in all analyses. Mean estimates of crown ages in millions of years are given for the three clades, followed by 95% highest posterior density intervals in parentheses. Specified settings in the last two rows indicate monophyly constraints according to alternative relationships among Osteoglossomorpha, Elopomorpha, and Clupeocephala^12, 14^. The age estimates obtained with the “permissive” gene set correspond to those shown in Fig. 2.

## Discussion

In this study, we generated draft genome assemblies of 14 representative cichlid species and developed a new Bayesian model to account for fossil-assignment uncertainty to estimate clade ages in a set of 90 teleosts with a particular focus on cichlid diversification times, using 58 re-evaluated fossil constraints. Our genomic timeline of cichlid diversification supports the conclusions of earlier studies (reviewed in ref. ^3^; Supplementary Table 9), which argued against Gondwanan vicariance, given that, e.g., the split between American and African cichlids occurred about 40 Myr after the separation of South America and Africa. On the other hand, our results are unable to distinguish between the two alternative scenarios of post-Gondwanan cichlid divergence: freshwater cichlids could have reached the different landmasses by oceanic dispersal^3^ or they could have undergone multiple transitions from marine to freshwater to colonize each landmass independently (Fig. 1b).

Both of these scenarios pose questions that our results are unable to answer: if cichlid fishes dispersed from Africa to South America around 62.1 Ma when the Atlantic Ocean was already around 900 km wide^30^, why is there no evidence of repeated dispersal between Africa and Madagascar across the much narrower Mozambique Channel, which had a width of only 400 km? And if each landmass should have been colonized independently by an unknown marine ancestor, why were most landmasses apparently colonized only once? Possible explanations for both questions are that perhaps secondary colonizations were unsuccessful due to competition with already-established local cichlid faunas, or that by chance alone the landmasses were colonized just once^31^.

Regardless of these remaining questions concerning the family’s early history, our new timeline of cichlid evolution will be valuable as the basis for the precise temporal reconstructions of the more recent “explosive” adaptive radiations of cichlid fishes that take place in the East African Lakes Tanganyika^31^, Malawi^32^, and Victoria^33^, as well as in numerous other lakes across central Africa and the Neotropics^34–36^. Owing to their increased precision, these reconstructions may then allow to address important questions about environmental triggers of these radiations such as the roles of lake-level fluctuations^37^ or ecological opportunity in newly formed lakes^38^, which could so far not be solved conclusively.

## Methods

### Species selection for whole-genome sequencing

The species for whole-genome sequencing were selected to cover a wide range of the native cichlid distribution worldwide, including South and Central America, India, Madagascar, Western and Eastern Africa, and to represent all cichlid subfamilies and multiple tribes of the subfamilies Cichlinae and Pseudocrenilabrinae^39^. Specimens of the species *Etroplus canarensis*, *Paratilapia polleni* “Andapa”, *Ptychochromis oligacanthus*, *Apistogramma diplotaenia*, *Australoheros scitulus*, *Amphilophus zaliosus*, *Bujurquina vittata*, *Andinoacara biseriatus*, *Heterochromis multidens*, *Tylochromis polylepis*, *Benitochromis conjunctus*, *Pelvicachromis taeniatus*, *Hemichromis elongatus*, and *Etia nguti* were obtained during field work in Cameroon and Zambia, provided by collaborators or museums, or purchased from the aquarium trade (Supplementary Table 1).

### Sequencing

We extracted genomic DNA from fin-clips using the E.Z.N.A Tissue DNA Kit (Omega Bio-Tek) including an RNAse treatment (5 μl, 100 mg/ml, for 2 min) and then sheared the DNA on a Covaris E220 (60 μl with 10% duty factor, 175 W, 200 cycles for 65 s). Library preparation was performed using the TruSeq DNA PCR-Free Sample Preparation kit (Low Sample Protocol) for 350 bp insert size. We measured the DNA concentration of each library with quantitative PCR and then performed paired-end sequencing of six libraries per lane with a read length of 126 bp on an Illumina HiSeq2500 instrument (v4 chemistry).

### Whole-genome assembly

De novo whole-genome assemblies were generated from the Illumina raw sequencing data for each individual following the approach described in Böhne et al.^40^ and Malmstrøm et al.^41^ using CeleraAssembler v.8.3^42^ and FLASh v.1.2.11^43^. Assembly quality and read coverage were evaluated with QUAST v.4.5^44^ (Supplementary Table 1). The completeness of the assemblies was assessed with BUSCO v.3^45^ using the BUSCO test library of 4584 conserved actinopterygian genes and specifying zebrafish as the reference species (Supplementary Table 2).

### Marker selection

As teleost fishes began to diverge over 200 Ma^46,47^ and their genomes have undergone duplications and frequent rearrangements^48^, it is difficult to reliably determine the orthology of most genomic regions across divergent teleost species. We therefore focused on conserved coding genes as the most suitable type of markers for phylogenomic inference, using a strategy that has already been applied successfully in several studies of teleost divergence times^14,18,49,50^. This strategy makes use of the information on gene relationships among teleost model species in the Ensembl database^51^ to exclude markers with evidence for duplications or deletions. Although previous applications of this strategy were limited to information for 10 teleost species present in the Ensembl database, a massive addition of teleost genomes in release 94 of the Ensembl database (published in October 2018) now allowed us to select markers based on gene relationships among 42 teleost species. We thus selected 3718 genes with a total of 19,995 exons, which all fulfilled the following criteria: (1) each gene had at least three exons with a minimum length of 150 bp; (2) each gene could be assigned to an Ensembl gene tree; (3) the gene tree included sequences for at least 40 of the 42 teleost species in the database; and (4) the gene tree did not indicate duplications on internal branches within teleosts. For each of the 19,995 exons, we then compared sequences of 15 representative teleost species out of the 42 species from the Ensembl database (Supplementary Tables 3 and 4). We quantified sequence similarity between zebrafish (*Danio rerio*), which was used as an outgroup, and 14 ingroup species by their TBLASTN^52^ bitscores, and we excluded exons unless the following conditions were met: (1) maximally 2 of the 14 ingroup species had bitscores below 50 and (2) all exon sequences that were orthologous according to the Ensembl gene tree had bitscores that were at least 20 units higher than all other sequences from the same genome. These bitscore thresholds were applied to ensure that orthologs could be reliably separated from paralogs for all selected markers. Following this filtering, we once again removed all genes with less than three remaining exons; the marker set then included 1247 genes with a total of 5869 exons. For each of these selected markers, zebrafish exon sequences were recorded together with the determined bitscore threshold value required for consideration as ortholog.

### Targeted assembly

As targeted assembly can yield greater contiguity in targeted regions compared with whole-genome assembly^53^, we also performed targeted exon assembly for the 14 newly sequenced cichlid species. To obtain a suitable set of closely related query sequences for these analyses, we repeated the marker selection described above with medaka (*Oryzias latipes*) as the outgroup and the genome assemblies for five cichlid species in the Ensembl database: *Amphilophus citrinellus*, *Oreochromis niloticus*, *Neolamprologus brichardi*, *Astatotilapia burtoni*, and *Metriaclima zebra*^1,35^. This resulted in a set of 10,590 medaka exon sequences in amino acid format and corresponding bitscore threshold values. For each of these sequences, we then used TBLASTN to identify the most similar homolog in the latest version of the Nile tilapia (*O. niloticus*) genome assembly^54^. As no homologs with bitscore values above the exon-specific treshold could be identified for 217 exons, this step produced a set of 10,373 tilapia exon sequences that we used as queries in the subsequent targeted assembly. We separately used the programs Kollector v.1.0.1^53^ and aTRAM v.2.0.alpha.5^55^ to assemble all target sequences. The assembler programs ABySS v.2.0.2^56^ and Trinity v.2.5.1^57^ were used internally by Kollector and aTRAM, respectively. For targeted assembly with Kollector, we set the maximum number of iterations to 6, the minimum match length for tagging reads to 0.5, the k-mer size for ABySS contig assembly to 32, the k-mer size for read overlap detection to 20, and the maximum number of k-mers to recruit to 1 million. For targeted assembly with aTRAM, we translated all nucleotide query sequences into amino acid sequences and then specified a timeout duration of 1000 s, an expected coverage of 10, and a minimum bitscore of 50. As the number of aTRAM iterations, we retained the default value of 5. We observed that assembly success depended more strongly on phylogenetic distance when Kollector was used compared to aTRAM (Supplementary Table 5), which may be explained by a greater degree of conservation in the amino acid query sequences that were used with aTRAM compared to the nucleotide query sequences used with Kollector. For each of the 14 newly sequenced cichlid genomes, the targeted and whole-genome assembly files were merged into a combined assembly file for subsequent TBLASTN searches.

### Species selection for phylogenomic analyses

To compile a dataset for phylogenomic inference, we complemented the combined assemblies for the 14 newly sequenced cichlid genomes with previously available genome assemblies for 76 teleost species (including four further cichlid species) and one non-teleost outgroup, for a total of 91 assemblies (Supplementary Tables 6 and 7). These species were selected to represent all major lineages within teleosts with increased sampling density of lineages closely related to cichlid fishes (e.g., the order Cyprinodontiformes and other groups within the series Ovalentaria). As a non-teleost outgroup, we included the spotted gar (*Lepisosteus oculatus*), a member of Holostei, the sister group of Teleostei^19^. We chose this sampling scheme, as it allowed us to use a wide range of fossil occurrences, including the earliest records of teleosts and their sister group, to calibrate the origin and the timeline of teleost evolution.

### Ortholog identification and filtering

Similar to the marker selection procedure, the identification and filtering of sequences orthologous to the selected markers followed the workflow established in Malmstrøm et al.^49^ and refined in Musilova et al.^14^. To compile a first set of candidate orthologs, we used each of the selected 5869 zebrafish exons as query and each of the 91 genome assemblies as subjects in TBLASTN searches. Per combination of exon query and assembly subject, we accepted the best hit as a candidate ortholog if its bitscore was above the exon-specific bitscore threshold recorded during the marker selection. The TBLASTN searches produced a total of 488,171 sequences for the 5869 exons. Per exon, we then aligned all candidate orthologs using MAFFT v.7.300^58^, while ensuring the integrity of codon triplets. Exon alignments were then filtered according to several criteria, aiming to exclude potentially remaining paralogs and to select the most suitable alignments for phylogenetic divergence-time estimation: (1) we excluded sequences with TBLASTN bitscore values lower than 90% of the highest bitscore value achieved by any of the ingroup sequences; this removed 130,321 out of 488,171 sequences across the 5869 exon alignments. (2) We calculated dN/dS between each ingroup sequence and the outgroup sequence using the software codeml of the PAML 4 package^59^, and removed sequences with dN/dS ratios > 0.25, as high dN/dS ratios can indicate positive selection, reading-frame shifts, or intronic regions. This filter removed 2470 sequences overall. (3) We excluded all exon alignments that had more than 10 missing sequences; this removed 4178 of the 5869 alignments. (4) We excluded all alignment codons that had at least one site with more than 20% missing data or a smoothed entropy-like score above 0.5, determined with the program BMGE v.1.1^60^. This filter removed 6035 codons and thus 18,105 bp out of 373,545 bp. (5) We excluded exon alignments shorter than 150 bp; this filter removed 71 of the 1690 remaining alignments. (6) As high GC-content variation has been shown to potentially misguide phylogenomic inference^61^, we excluded all exons with an among-sequence SD in GC content above 0.04; this removed 247 of the remaining 1619 exon alignments. (7) We excluded the exons of all genes if these genes did not have at least three exons remaining in the dataset or if their exons were over 100,000 bp apart on the zebrafish genome; this removed 453 of the remaining 1372 exon alignments. (8) We excluded all exons with exon trees that were significantly discordant to the trees of other exons of the same gene, which could potentially result from paralogy. As in Malmstrøm et al.^49^ and Musilova et al.^14^, these concordance tests were performed with the program Concaterpillar v.1.7.2^62^, which internally used RAxML v.7.2.8^63^ and the generalized time-reversible (GTR) model of sequence evolution^64^ for maximum-likelihood tree inference. Following the concordance tests, we concatenated, per gene, the alignments of all exons with concordant exon trees. Genes that did not have at least three exons with concordant exon trees were removed. (9) The concatenated exon alignments of all remaining 161 genes were visually checked to avoid homology errors^65^ and two genes were excluded due to potential misalignment.

Finally, we quantified the substitution rate and its among-species variation for each gene in separate molecular-clock analyses with the Bayesian software BEAST 2 v.2.5.0^18^, and we selected two nested sets of genes according to different thresholds for these parameters. The models used in these BEAST 2 analyses assumed the uncorrelated lognormal (UCLN) relaxed molecular clock^66^ and a pure-birth speciation process^67^, and the bModelTest package^68^ for BEAST 2 was employed to average over nucleotide substitution models. In each BEAST 2 analysis, the Markov-chain Monte Carlo (MCMC) process was run for 50 million iterations, which produced effective samples sizes (ESS) for all model parameters of at least 200 for all but 14 genes (ESS values reached at least 100 in all but 5 genes). As low ESS values can result from conflicting phylogenetic signals within genes or low information content of the alignment, we considered these ESS values in our selection of genes for further phylogenomic analyses. Thus, we selected a “permissive” set of genes that included all genes with a minimum ESS value above 100, a substitution rate below 1.6 × 10^−9^ per year and site, and a coefficient of rate variation below 0.7; this set included 147 genes with a total alignment length of 127,638 bp. In addition, we selected a “strict” set of genes as a subset of the “permissive” set that included all genes with a minimum ESS value above 200, a substitution rate below 1.4 × 10^−9^ per year and site, and a coefficient of rate variation below 0.6; this set included 77 genes with a total alignment length of 62,776 bp (Supplementary Table 8). The two sets complemented each other as the “permissive” set was expected to be more phylogenetically informative due to its larger size and higher mean substitution rate, whereas the “strict” set was expected to contain less homoplasies and evolve in a more clock-like manner, both of which could lead to more accurate age estimates.

### Species-tree inference

We performed species-tree inference with both the “permissive” and the “strict” set of genes, and applied both the multi-species coalescent model and concatenation. Analyses with the multi-species coalescent model were conducted with the software ASTRAL-III v.5.6.3^23^, using maximum-clade credibility summary trees of the per-gene BEAST 2 analyses as input. The concatenated alignments of the “permissive” and “strict” gene sets were separately analyzed with BEAST 2, applying the birth–death tree prior^69^, the UCLN relaxed clock model, and independent GTR site models with γ-distributed rate variation for eight (“permissive”) and five (“strict”) partitions. One-over-x prior distributions were placed on the mean mutation rates of each partition and an exponential prior distribution (with a mean of 0.5) was used for the SD of among-branch rate variation. The partitions were selected from the set of all first and second codon positions, using the rcluster algorithm of the software PartitionFinder v.2^70,71^, with equal weights for all model parameters and a minimum partition size of 5000 sites. Third codon positions were exluded from this set of BEAST 2 analyses to reduce both the computational demand and the degree of saturation in the alignment. As these BEAST 2 analyses aimed to infer the species-tree topology rather than its divergence times, they were time-calibrated only by an age constraint on the root node, which was arbitrarily set to 300 Ma with a SD of 0.1 Myr. To facilitate MCMC convergence, a single monophyly constraint was placed to group Syngnatharia and Pelagiaria. The sister-group relationship of these two clades is overwhelmingly supported by molecular data^12,13,19^, but is difficult to infer in molecular-clock analyses due to highly divergent substitution rates of the two lineages. For each of the “permissive” and “strict” gene sets, we performed 10 replicate BEAST 2 analyses, each with 300 million MCMC iterations. Convergence of the MCMC was confirmed by ESS values of at least 200 (“permissive”) or 400 (“strict”) for all model parameters.

### Accounting for fossil-assignment uncertainty

The CladeAge add-on package for BEAST 2^18^ replaces the specification of lognormal, normal, uniform, or other types of distributions for prior densities with automatically calculated prior densities that are shaped according to expectation for clade ages under certain assumptions for diversification parameters and the fossil sampling rate. This calibration framework is well suited to account for uncertainty in fossil assignment. With a single unambiguous first record of a clade, CladeAge calibration densities correspond to the probability density that the clade originated at time *t* before the age of the first record (thus, *t* ≥ 0),

1 $$f(t)={\mathbb{E}}[\psi N{{\rm{e}}}^{-\psi S(t)}| N\ge 1],$$

where *ψ* is the fossil sampling rate, *N* is the number of species surviving at the time of fossilization, and *S*(*t*) is the sum of lineage durations between clade origin and the time of fossilization, with both *N* and *S*(*t*) being stochastic outcomes of a time-homogeneous diversification process (see Matschiner et al.^9^ for the derivation of this probability density).

We now extend this model so that when two fossils *q*_younger_ and *q*_older_ with ages *t*_younger_ and *t*_older_ (*t*_older_ > *t*_younger_) are both possible first occurrences of a clade with probabilities *p*_younger_ and *p*_older_ = 1 − *p*_younger_, respectively, the calibration density *f*(*t*) is calculated as the sum of two individual calibration densities *f*_younger_ and *f*_older_, weighted according to their relative probabilities:

2 $${f}_{{\rm{younger}}}(t)={\mathbb{E}}[\psi N{{\rm{e}}}^{-\psi S(t-{t}_{{\rm{younger}}})}| N\ge 1]$$

3 $${f}_{{\rm{older}}}(t)={\mathbb{E}}[\psi N{{\rm{e}}}^{-\psi S(t-{t}_{{\rm{older}}})}| N\ge 1]$$

4 $$f(t)=\left\{\begin{array}{ll}{p}_{{\rm{younger}}}{f}_{{\rm{younger}}}(t),&{\rm{if}}\,t\,<\,{t}_{{\rm{older}}}.\\ {p}_{{\rm{younger}}}{f}_{{\rm{younger}}}(t)+{p}_{{\rm{older}}}{f}_{{\rm{older}}}(t),&{\rm{if}}\,t\ge {t}_{{\rm{older}}}.\end{array}\right.$$

The probabilities *p*_younger_ and *p*_older_ are to be set by the user. In this framework, it is assumed that the younger one of the two fossils is unambiguously assigned to the clade, while the assignment of the older one is questionable, in which case the probability that the older fossil is the first record of the clade, *p*_older_, equals the probability of its correct assignment to the clade. On the other hand, the probability that the younger fossil is the clade’s first record, *p*_younger_, equals the probability that the older fossil is incorrectly assigned (1 − *p*_older_). Thus, to set both *p*_younger_ and *p*_older_, the reliability of the assignment of the older fossil needs to be considered. When the user is unable to decide whether the assignment of the older fossil is more likely to be correct than false, the naive specification of identical probabilities *p*_younger_ = *p*_older_ = 0.5 may be appropriate. If, however, it appears more likely that the assignment is correct, a *p*_older_ > 0.5 should be chosen (and a *p*_older_ below 0.5 should be chosen if it appears more likely to be incorrect). Uncertainties in fossil ages can be accounted for as in the case of a single unambiguous first record (see Matschiner et al.^9^ for details). Uncertainties in fossil ages can be accounted for as in the case of a single unambiguous first record (see Matschiner et al.^9^ for details).

Depending on the model parameter values and the difference between *t*_younger_ and *t*_older_, the calibration density *f*(*t*) can be uni- or bimodal (Supplementary Fig. 5). To test whether bimodal calibration densities could lead to poor MCMC convergence, we performed a series of analyses with increasing temporal distance (30, 60, 90, and 120 Myr) between two simulated fossils, leading to increasingly pronounced bimodality. No further age constraints were used in these analyses and uninformative priors were applied to speciation and clock rates. In each case, the posterior density for the calibrated node corresponded to the specified calibration density and the MCMC trace revealed that the chain switched frequently between the two peaks; thus, we found no signs of poor convergence (Supplementary Fig. 5).

### Phylogenetic divergence-time estimation

Teleost divergence times were estimated based on the CladeAge approach^9^ in which calibration priors are calculated from estimates of fossil age, diversification rates, and the fossil sampling rate. The model underlying this type of divergence-time estimation assumes that prior distributions are defined for all clades that fulfill the following three conditions: (1) the clade must be represented in the fossil record, (2) the clade must be morphologically recognizable so that fossils can be assigned directly to it, not only indirectly through assignment to a subclade, and (3) all potential sister lineages of this clade must be present in the phylogeny so that the origin of this clade is guaranteed to be represented by a node in the phylogeny^9^. For 51 clades that matched the criteria for CladeAge calibrations, we identified the earliest fossil occurrences, determined their geological stages and the absolute ages of these stages, and used these to define age constraints with CladeAge (Supplementary Note 2). The first occurrences of seven clades were found to be ambiguous with two fossils in each case that could potentially represent the clades’ earliest records. In these cases, both potential first occurrences were used in the analyses, with weights as specified in Supplementary Note 2. We assumed the same estimates for the teleost fossil sampling rate (0.0066–0.01806 per lineage per Myr^72^), their net diversification rate (0.041–0.081 per lineage per Myr^73^), and their turnover (0.0011–0.37 per lineage per Myr^73^) as in Matschiner et al.^9^. To fix the tree topology to that of the species tree inferred from the concatenated “permissive” gene alignments (see above), we used this species tree as the starting tree and disabled all topology operators. As in the earlier analyses of the species-tree topology based on concatenation, we performed phylogenetic divergence-time estimations separately with both the “permissive” and the “strict” set of gene alignments, and we applied the same partitioning schemes as in these earlier analyses. The settings for the assumed substitution model (the GTR model with γ-distributed rate variation) and the tree prior (the birth–death tree prior) were also identical to the earlier analyses of the species-tree topology. We again performed ten replicate BEAST 2 analyses for both the “permissive” and the “strict” gene set, in each case with 100 million MCMC iterations. These analyses produced ESS values > 200 (“permissive”) or 1000 (“strict”) for all model parameters.

We assessed the robustness of our divergence-time estimates with a range of re-analyses that were identical to those with the “strict” dataset except that (1) MCMC sampling was done without data, only from the prior distributions; (2) all cichlid fossils were excluded; (3) the fossil sampling rate assumed for CladeAge calibrations was doubled or halved; (4) the net diversification rate assumed for calibrations was doubled or halved; or (5) the interrelationship of Osteoglossomorpha, Elopomorpha, and Clupeocephala (all remaining teleosts) was constrained so that Osteoglossomorpha were either the sister group to Elopomorpha^14,74^ or to Clupeocephala^12,19^.

### Reporting summary

Further information on research design is available in the Nature Research Reporting Summary linked to this article.

## Supplementary information

Reporting summary

Supplementary information

## Data Availability

Data generated for this study are available from NCBI under the BioProject accession number PRJNA550295. Previously available datasets used in this study are either hosted at the Ensembl (Ensembl.org), NCBI (ncbi.nlm.nih.gov), or EBI (ebi.ac.uk) databases, or deposited on datadryad.org, figshare.com, parrot.genomics.cn, surfdrive.surf.nl, cichlid.gurdon.cam.ac.uk, efishgenomics.integrativebiology.msu.edu, or creskolab.uoregon.edu (see Supplementary Table 7 for details). Sequence alignments used for phylogenomic inference are available from http://evoinformatics.eu/continental.htm. Figure 2 and Supplementary Figs. 1–4, 6, and 7 have associated raw data available from http://evoinformatics.eu/continental.htm.

## Code availability

Code for computational analyses is available from Github (http://github.com/mmatschiner/continental).
