# Supplementary material for: The genomic timeline of cichlid fish diversification across continents
Source: Nat Commun. 2020 Nov 18;11:5895. doi: 10.1038/s41467-020-17827-9 (PMC7674422; doi:10.1038/s41467-020-17827-9)
Supplement: Supplementary file 1 — Reporting summary [file 41467_2020_17827_MOESM1_ESM.pdf]

nature research

Corresponding author(s): Michael Matschiner, Walter Salzburger

Last updated by author(s): Jul 9, 2020

Reporting Summary

Nature Research wishes to improve the reproducibility of the work that we publish. This form provides structure for consistency and transparency in reporting. For further information on Nature Research policies, see Authors & Referees and the Editorial Policy Checklist .

Statistics

For all statistical analyses, confirm that the following items are present in the figure legend, table legend, main text, or Methods section.

n/a	Confirmed	
☒	☐	The exact sample size (*n*) for each experimental group/condition, given as a discrete number and unit of measurement
☒	☐	A statement on whether measurements were taken from distinct samples or whether the same sample was measured repeatedly
☒	☐	The statistical test(s) used AND whether they are one- or two-sided<br>*Only common tests should be described solely by name; describe more complex techniques in the Methods section.*
☒	☐	A description of all covariates tested
☒	☐	A description of any assumptions or corrections, such as tests of normality and adjustment for multiple comparisons
☐	☒	A full description of the statistical parameters including central tendency (e.g. means) or other basic estimates (e.g. regression coefficient) AND variation (e.g. standard deviation) or associated estimates of uncertainty (e.g. confidence intervals)
☒	☐	For null hypothesis testing, the test statistic (e.g. *F*, *t*, *r*) with confidence intervals, effect sizes, degrees of freedom and *P* value noted<br>*Give P values as exact values whenever suitable.*
☐	☒	For Bayesian analysis, information on the choice of priors and Markov chain Monte Carlo settings
☒	☐	For hierarchical and complex designs, identification of the appropriate level for tests and full reporting of outcomes
☒	☐	Estimates of effect sizes (e.g. Cohen's *d*, Pearson's *r*), indicating how they were calculated

Our web collection on statistics for biologists contains articles on many of the points above.

Software and code

Policy information about availability of computer code

Data collection	No software was used in the data collection process. Sequencing data were generated through Illumina HiSeq sequencing at the Norwegian Sequencing Centre, Oslo, Norway.
Data analysis	Whole-genome assembly: CeleraAssembler v.8.3, FLASh v.1.2.11, QUAST v.4.5, BUSCO v.3; Marker selection: BLAST v.2.29; Targeted assembly: Kollector v.1.0.1, aTRAM v.2.0.alpha.5, ABySS v.2.0.2, Trinity v.2.5.1; Ortholog identification and filtering: MAFFT v.7.300, PAML 4, BMGE v.1.1, Concaterpillar v.1.7.2, RAxML v.7.2.8; BEAST v.2.5.0; Species-tree inference: ASTRAL v.5.6.3, PartitionFinder v.2. All custom code is available from https://github.com/mmatschiner/continental.

For manuscripts utilizing custom algorithms or software that are central to the research but not yet described in published literature, software must be made available to editors/reviewers. We strongly encourage code deposition in a community repository (e.g. GitHub). See the Nature Research guidelines for submitting code & software for further information.

Data

Policy information about availability of data

All manuscripts must include a data availability statement. This statement should provide the following information, where applicable:

- Accession codes, unique identifiers, or web links for publicly available datasets
- A list of figures that have associated raw data
- A description of any restrictions on data availability

Data generated for this study are available from NCBI under the BioProject accession numbers PRJNA552202 and PRJNA550295. Previously available datasets used in this study are either hosted at the Ensembl (Ensembl.org), NCBI (ncbi.nlm.nih.gov), or EBI (ebi.ac.uk) databases or deposited on datadryad.org, figshare.com, parrot.genomics.cn, surfdrive.surf.nl, cichlid.gurdon.cam.ac.uk, efishgenomics.integrativebiology.msu.edu, or creskolab.uoregon.edu (see Supplementary Table 7 for details). Sequence alignments used for phylogenomic inference are available from http://evoinformatics.eu/continental.htm.
Figure 2, Supplementary Figures 1-4, and Supplementary Figures 6-7 have associated raw data available from http://evoinformatics.eu/continental.htm.

Field-specific reporting

Please select the one below that is the best fit for your research. If you are not sure, read the appropriate sections before making your selection.

☐ Life sciences ☐ Behavioural & social sciences ☒ Ecological, evolutionary & environmental sciences

For a reference copy of the document with all sections, see nature.com/documents/nr-reporting-summary-flat.pdf

Ecological, evolutionary & environmental sciences study design

All studies must disclose on these points even when the disclosure is negative.

Study description	In this study, we investigate the timeline of cichlid fish diversification across continents with newly generated genome sequences and a new model for phylogenetic divergence-time estimation.
Research sample	Sampling for whole-genome sequencing included one specimen of each of 14 species of cichlid fishes. Samples used for this study were selected from a larger set of available samples so that all major geographic and taxonomic groups of cichlids were represented (see below for details on the sampling strategy). All samples were obtained from zoos, collaborators, field excursions described in other publications, and the aquarium trade, as listed in Supplementary Table 1. The 14 species are as listed below, with information on sample sex (m: male; f: female; u:unknown) in parentheses:<br>Etroplus canarensis (u)<br>Paratilapia polleni “Andapa” (u)<br>Ptychochromis oligacanthus (m)<br>Apistogramma diplotaenia (m)<br>Australoheros scitulus (m)<br>Amphilophus zaliosus (m)<br>Bujurquina vittata (m)<br>Andinoacara biseriatus (u)<br>Heterochromis multidens (u)<br>Tylochromis polylepis (f)<br>Benitochromis conjunctus (m)<br>Pelvicachromis taeniatus (m)<br>Hemichromis elongatus (m)<br>Etia nguti (u)<br>(The ages are unknown for all samples.) This sample of cichild species is highly representative for their global diversity.<br><br>In addition to these 14 species, we included publicly available data for 77 further fish species. These datasets were taken either from the Ensembl (Ensembl.org), NCBI (ncbi.nlm.nih.gov), or EBI (ebi.ac.uk) databases or from deposits on datadryad.org, figshare.com, parrot.genomics.cn, surfdrive.surf.nl, cichlid.gurdon.cam.ac.uk, efishgenomics.integrativebiology.msu.edu, or creskolab.uoregon.edu (full links to all datasets are provided in Supplementary Table 7).
Sampling strategy	The 14 cichlid species for whole-genome sequencing were selected to cover a wide range of the native cichlid distribution worldwide, including South and Central America, India, Madagascar, Western and Eastern Africa, and to represent all cichlid subfamilies and multiple tribes of the subfamilies Cichlinae and Pseudocrenilabrinae. In cases where multiple samples were available for a clade so that their joint inclusion would have been redundant, the sample with the highest quality of the genomic data was selected. The sample size was determined to be sufficient because the selected lineages included all cichlid clades with fossil records for time calibration with our new Bayesian approach.
Data collection	Whole-genome sequencing data were generated by Illumina HiSeq sequencing on an Illumina HiSeq2500 machine with v4 chemistry at the Norwegian Sequencing Centre (NSC), Oslo, Norway. Genome sequencing libraries were prepared by authors A.B. and F.R.; the Illumina machine was operated by NRC staff members.
Timing and spatial scale	Not applicable as no field work was conducted for this study; all samples were already available before the start of the study.
Data exclusions	Whole-genome sequencing data were filtered to identify the most suitable sequences for phylogenomic inference. the criteria for the selection of genomic regions for phylogenomic analyses were pre-established in earlier studies (e.g. Roth O, Solbakken MH, Tørresen OK et al. (2020) Evolution of male pregnancy associated with remodeling of canonical vertebrate immunity in seahorses and pipefishes. Proceedings of the National Academy of Sciences USA, 117, 9431–9439.). All steps of the filtering pipeline are described in Supplementary Information 6, and the applied custom code is available from https://github.com/mmatschiner/continental.
Reproducibility	To enable the reproduction of our result by other researchers, we provide all datasets, analysis code, and input files for certain programs on https://github.com/mmatschiner/continental and http://evoinformatics.eu/continental.htm. As part of our study, reproducibility was confirmed for specific analyses, such as all BEAST 2 analyses, for which we performed three replicates analyses with each dataset, and additionally sets of analyses with different datasets, that all supported the same result. Overall, all attempts at replication were successful, and no results can not be reproduced.
Randomization	Randomization is not relevant for our study as all samples were used for all analyses.

Blinding	Species-tree inference was not constrained according to previously available taxonomic information; thus, the applied methods were blind to taxonomic groupings, some of which have been established beyond doubt by past research. The inferred species tree agreed with all established groupings.

Did the study involve field work? ☐ Yes ☒ No

Reporting for specific materials, systems and methods

We require information from authors about some types of materials, experimental systems and methods used in many studies. Here, indicate whether each material, system or method listed is relevant to your study. If you are not sure if a list item applies to your research, read the appropriate section before selecting a response.

Materials & experimental systems

n/a	Involved in the study	
☒	☐	Antibodies
☒	☐	Eukaryotic cell lines
☒	☐	Palaeontology
☒	☐	Animals and other organisms
☒	☐	Human research participants
☒	☐	Clinical data

Methods

n/a	Involved in the study	
☒	☐	ChIP-seq
☒	☐	Flow cytometry
☒	☐	MRI-based neuroimaging
