# Supplementary material for: The genomic timeline of cichlid fish diversification across continents
Source: Nat Commun. 2020 Nov 18;11:5895. doi: 10.1038/s41467-020-17827-9 (PMC7674422; doi:10.1038/s41467-020-17827-9)
Supplement: Supplementary file 2 — Supplementary information [file 41467_2020_17827_MOESM2_ESM.pdf]

Supplementary Information

The genomic timeline of cichlid fish diversification across continents

Contents

Supplementary Notes 1-2 2
Supplementary Figures 1-7 15
Supplementary Tables 1-9 22

Supplementary Notes

Supplementary Note 1: Consequences of fossil-assignment uncertainty.
The fossil record of a clade informs about the age of origin of that clade. In particular, the oldest known fossil of a clade indicates its minimum age. Unfortunately, however, the assignment of fossils to clades is often ambiguous when fossils are fragmentary or when morphological characters evolved convergently in multiple clades. For many clades, it is therefore uncertain if one or another fossil represents the earliest record of that clade.

A prominent example of this issue is the teleost fish order Tetraodontiformes, the earliest record of which has long been assumed to be †*Plectocretacicus clarae* from the limestone of Haqil, Israel [1, 2] with an age of 100.3-98.0 Ma (Supplementary Note 2). The tetraodontiform affinity of the fossil has recently been reevaluated (see discussion in[3]), and many authors [4–9] have since opted not to accept †*Plectocretacicus clarae* as the earliest tetraodontiform record. Exhibiting more tetraodontiform synapomorphies than †*Plectocretacicus clarae*, the earliest undisputed tetraodontiform record is †*Cretatriacanthus guidottii* from the Calcari di Melissano, Italy, with an age of 89.8-83.0 Ma (Supplementary Note 2). However, ambiguity remains regarding the status of †*Plectocretacicus clarae*, and a recent combined analysis of morphological and molecular data supported its original placement in Tetraodontiformes [10]. To address this ambiguity, several recent studies of teleost divergence times conducted separate analyses with and without the age constraint based on †*Plectocretacicus clarae* [7–9]. Unfortunately, the results of these studies highlight the strong impact of the fossil on a large part of the timeline of teleost evolution. For example, the age estimate for the origin of Acanthomorphata, a group comprising roughly a third of vertebrate diversity, shifted by over 10 Myr in the analyses of Musilova et al.[9], depending on whether or not †*Plectocretacicus clarae* was used as an age constraint for Tetraodontiformes. Frustratingly, this means that conclusions drawn from these timelines may only be valid under a certain assumption for the position of †*Plectocretacicus clarae*. Furthermore, the approach of repeating phylogenetic divergence-time estimation separately with and without ambiguous constraints is only feasible when the investigated group has few such ambiguities, as the number of possible combinations of constraints grows exponentially with each clade that has an ambiguous first record.

Instead of conducting phylogenetic divergence-time estimation separately with and without all ambiguous constraints, accounting for all uncertainties in a single Bayesian analysis would have the advantages that only a single set of results would be obtained, which would facilitate the interpretation, and that the results could be obtained at lower computational cost. When morphological characters have been scored for both fossils and extant species, this type of analysis is possible with so-called "tip-dating" approaches that consider fossils as terminal taxa and can infer their position as part of the analysis [11–13]. In the absence of a morphological character matrix, a recently implemented feature of the program PhyTime [14, 15] allows specifying probabilities for the assignment of a fossil to different clades; however, PhyTime allows only uniform calibration densities with hard upper boundaries that are usually selected arbitrarily and approximate the *a priori* expectations of clade ages only poorly [16–19]. In addition, PhyTime does not allow the joint estimation of tree topology and divergence times. To address these limitations in divergence-time estimation without morphological character matrices, we here develop and apply a new approach that allows the

specification of two alternative first occurrences, with equal or unequal probability weights, for each clade, and we implement this approach in the CladeAge package v.2.0.0 for BEAST 2 [18].

Supplementary Note 2: Fossil constraints.
In the following, we discuss, for each clade that fulfills the conditions for use as CladeAge calibration point, the first record of the clade and the age of this record. The nomenclature follows that of Betancur-R. et al.[20], and ages of geological stages are according to the ICS international chronostratigraphic chart v.2018/8 [21] unless noted otherwise. All clades listed below are considered to include stem lineages.

Holostei: †*Watsonulus eugnathoides* (Piveteau, 1935) (251.9-251.2 Ma) or †*Acentrophorus varians* Kirkby, 1862 (262.1-259.1 Ma). Holostei combine Amiiformes (with the only extant species *Amia calva*), Lepisosteiformes (with the extant family Lepisosteidae) and the extinct order Semionotiformes [22–24].
†*Watsonulus eugnathoides* from the Middle Sakamena Formation at Ambilombe Bay has been placed within the crown of Holostei or as a stem member of Holostei in number of studies [3, 24]. The Middle Sakamana Formation is dated as Induan (see discussion in[3]) with an age between 251.9-251.2 Ma. †*Watsonulus eugnathoides* is predated by †*Acentrophorus varians* [25] from the Raisby Formation at Fulwell Hill (Sunderland, UK). This species has been accepted by some authors as the earliest record of Holostei [26] but its phylogenetic placement within the group has been questioned [3]. The Raisby Formation was deposited during cycle 1 of the English Zechstein sequence [27], which represents the upper part of the Capitanian [28]. This age can be interpreted as 262.1-259.1 Ma. We here consider both †*Watsonulus eugnathoides* and †*Acentrophorus varians* as equally probable first occurrences of Holostei (weights: 0.5/0.5). Our phylogenomic data set includes a single representative of Holostei, *Lepisosteus oculatus*.

Teleostei: †*Prohalecites porroi* (Tintori, 1990) (239.5-237.0 Ma).
The earliest records of total-group Teleostei include Pholidophoriformes as well as †*Prohalecites* [29, 30]. Of these, †*Prohalecites porroi* from the middle Kalkschieferzone (Meride Kalk) near Ca' del Frate (Viggiú, Varese, Italy) [31] represents the first occurrence of teleosts [3, 29]. Based on palynomorphology, a latest Ladinian age has been suggested for †*Prohalecites porroi* [32]. Here, this age is interpreted as 239.5-237.0 Ma. Our phylogenomic data set includes 91 members of Teleostei.

Osteoglossomorpha: †*Lycoptera* (Sauvage, 1880) (131.0-124.4 Ma).
The earliest records of Osteoglossomorpha are provided by members of the *Lycoptera* assemblage of the Chinese Jehol Biota [33]. *Lycoptera* first appear in the Jingangshan Bed of the Yixian Formation [34]. The fish fossils underlie a tuff layer for which a minimum age of 124.4 was directly determined [34]. The maximum age of the *Lycoptera* fossils comes from the maximum age of the Yixian Formation, 131 Ma [35]. Our phylogenomic data set includes the osteoglossid *Scleropages formosus* as a representative of Osteoglossomorpha.

Elopomorpha: †*Anaethalion zapporum* Arratia, 2000 (152.6-152.1 Ma).
The oldest representative of Elopomorpha is †*Anaethalion zapporum* from the Nusplingen lithographic limestone (Swabia, Germany) [3, 36]. The ammonite fauna present at Nusplingen indicates an age within the Ulmense Subzone of the latest Kimmeridgian [37]. This subzone is estimated to

be maximally 0.5 Ma older than the Kimmeridgian/Tithonian boundary [38]. Our phylogenomic data set includes a single representative of Elopomorpha, the anguillid *Anguilla anguilla*.

Clupeocephala: †*Leptolepides haerteisi* Arratia, 1997, (152.1-150.9 Ma) or †*Leptolepides* sp. [39] (153.9-152.2 Ma).
In the absence of known stem-group fossils, the earliest record of Clupeocephala is provided by crown-group fossils of the euteleosteomorph genus †*Leptolepides* that have been reported from Tithonian and Kimmeridgian lithographic limestones of Germany and France. The oldest of these are fossils assigned to †*Leptolepides haerteisi* from the Zandt Member of the Solnhofen Formation (Eichstätt, Germany). Based on its ammonite fauna the Zandth Member can be constrained to the Tithonian *Hybonoticeras hybonotum* Ammonite Zone [3] with an age of 152.1-150.9 Ma [40]. Older fossils assigned to †*Leptolepides* occur in Kimmeridgian deposits of Cerin (France) and Nusplingen and Wattendorf (Germany), but since these have never been analyzed in as much detail as †*Leptolepides haerteisi*, Benton et al.[3] did not consider these sufficiently reliable to be used as age constraints. The earliest Kimmeridgian records of †*Leptolepides* are from the Wattendorf Limestone [39], which can be constrained to the *Aulacostephanus eudoxus* Ammonite Zone [3] with an age of 153.9-152.2 Ma [40]. We here consider both †*Leptolepides haerteisi* and †*Leptolepides* sp. as equally probable first occurrences of Clupeocephala (weights: 0.5/0.5). Our phylogenomic data set includes 88 representatives of Clupeocephala.

Otomorpha: †*Tischlingerichthys viohli* Arratia, 1997 (152.1-150.9 Ma).
No stem-group fossils are known for Otomorpha, thus the earliest crown-group member, represented by the ostariophysan †*Tischlingerichthys viohli* from the Mörnsheim Formation (Mühlheim, Germany), marks the appearance of Otomorpha in the fossil record [3, 41]. The Mörnsheim Formation lies within the *Hybonoticeras hybonotum* Ammonite Zone [3] with an age of 152.1-150.9 Ma [40]. Our phylogenomic data set includes four representatives of Otomorpha.

Clupeiformes: †*Ezkutuberezi carmeni* Poyato-Ariza, López-Horgue, and García-Garmilla, 2000 (139.8-125.0 Ma).
A number of clupeiform genera appear in the fossil record of the Early Cretaceous. Among the oldest specimens are †*Diplomystus kokuraensis* and †*Diplomystus primotinus* from the Kanmon Group at Kitakyushu City (Kyushu Island, Japan). The two specimens of †*Diplomystus* have been discovered in the upper layer of the Wakino subgroup [42], which was claimed to be Neocomian (Berriasian-Hauterivian) by the original authors, but is more likely Barremian [43] or Hauterivian-Barremian [44]. Approximately the same age are †*Ellimmichthys longicostatus* and †*Scutatuspinosus itapagipensis* from the Marfim Formation at Itapagipe (Recôncavo Basin, Brazil), that is also assumed to be Hauterivian-Barremian in age [45, 46]. However, the four species might be predated by †*Ezkutuberezi carmeni* from the Villaro Formation in the Arratia Valley close to Bilbao (Basque Country, Spain) [47]. Through correlation with the Bárcena Mayor Formation, a Valangian-Barremanian age is supported for the Villaró Formation [48]; thus, the age of †*Ezkutuberezi carmeni* lies between 139.8-125.0 Ma. Our phylogenomic data set includes *Clupea harengus* as a single representative of Clupeiformes.

Cypriniformes: †*Amyzon* spp. (Wilson, 1980) (60.7-59.9 Ma).
The oldest known cypriniforms are catostomids of the genus †*Amyzon* from the Paskapoo Formation (Alberta, Canada) [49, 50]. These fossils of the Paskapoo Formation are from "Site 5 at or near Erickson's Landing" [51], which corresponds to the Ti3 stage of the Tiffanian North American Land Mammal Age (NALMA), with an age of 60.7-59.9 Ma [52]. Cypriniformes are represented in our phylogenomic data set by *Danio rerio*.

Characiformes: †*Santanichthys diasii* (Silva Santos, 1958) (126.3-113.0 Ma).
The earliest record of Characiformes is provided by the stem-characiform †*Santanichthys diasii* [53–55]. The species is reported from the Brazilian Santana, Riachuelo, and Codó Formations, with the best-investigated specimens coming from the Santana Formation [53]. Of the three formations, the ages of the Santana and Riachuelo Formations are given as Aptian-Turonian [56] and Upper Aptian-Albian [57], respectively, but the age of the Codó Formation can be limited to the Aptian based on palynology [58]. Thus, †*Santanichthys diasii* dates to 126.3-113.0 Ma. Our phylogenomic data set includes a single representative of Characiformes, *Astyanax mexicanus*.

Euteleosteomorpha: †*Leptolepides haerteisi* Arratia, 1997, (152.1-150.9 Ma) or †*Leptolepides* sp. [39] (153.9-152.2 Ma).
The earliest records of Clupeocephala are simultaneously the oldest euteleosteomorphs. See above for details. Our phylogenomic data set includes 84 representatives of Euteleosteomorpha.

Esociformes: †*Estesesox foxi* Wilson et al., 1992 (84.5-83.5 Ma).
The earliest fossil record of Esociformes is provided by specimens of †*Estesesox foxi* from the Milk River Formation (Alberta, Canada) [59]. According to U–Pb geochronology, the Milk River Formation was deposited between 84.5-83.5 Ma [60]. Esociformes are represented in our phylogenomic data set by *Esox lucius*.

Salmoniformes: †*Eosalmo driftwoodensis* Wilson, 1977 (52.1-51.4 Ma).
The oldest salmoniform record is provided by †*Eosalmo driftwoodensis* from the Driftwood Creek beds near Smithers (British Columbia, Canada) [61, 62]. Based on palynology and U–Pb zircon analysis, the age of the Drifwood Creek beds can be constrained as 52.1-51.4 Ma [63, 64]. Our phylogenomic data set includes *Salmo salar* as a representative of Salmoniformes.

Stomiatiformes: †*Paravinciguerria praecursor* Arambourg, 1954 (94.3-93.7 Ma).
†*Paravinciguerria praecursor* is known from the deposits of Jbel Tselfat (Morocco) and the Argille Varicolori succession near Floresta and Malvagna (Sicily, Italy) and is generally regarded as the earliest member of Stomiatiformes [65]. Both the deposits of Jbel Tselfat and the bituminous shales of the Argille Varicolori succession are associated with the Oceanic Anoxic Event 2 (OAE2) close to the Cenomanian-Turonian boundary [65, 66], and can be constrained to 94.3-93.7 Ma based on nannofossils [67, 68].

Osmeriformes: †*Speirsaenigma lindoei* Wilson and Williams, 1991 (60.7-59.9 Ma).
Of the few osmeriform fossils predating the Oligocene, the earliest record is †*Speirsaenigma lindoei* from the Paskapoo Formation (Alberta, Canada) [69, 70]. The specimens were collected from the "fish layer" at the Joffre Bridge Roadcut locality [69], the age of which falls within the Ti3 stage

of the Tiffanian North American Land Mammal Age (NALMA) [71] that can be constrained to 60.7-59.9 Ma [52]. Our phylogenomic dataset included the two osmeriforms *Osmerus eperlanus* and *Protosalanx hyalocranius*.

Osmeridae: †*Enoplophthalmus schlumbergeri* Sauvage, 1880 (33.9-32.6 Ma Ma).
The earliest records of the family Osmeridae are Oligocene in age [70], with the first appearance marked by †*Enoplophthalmus schlumbergeri* in the Campagne-Calavon Formation at Céreste (Alpes-de-Haute-Provence, France) [72, 73]. Through correlation with the Ronzon (France) and Hoogbutsel (Belgium) beds, the fossil age can be constrained to MP 21 [72] and thus to 33.9-32.6 Ma [52]. Of the three families within the suborder Osmeroidei, Plecoglossidae and Salangidae are sister groups [20]; therefore, the age of Osmeridae is identical to the time of divergence between Salangidae and Osmeridae, represented in our phylogenomic data set by *Protosalanx hyalocranius* and *Osmerus eperlanus*.

Aulopiformes: Alepisauroidei *indet.* Kriwet, 2003 (130.8-126.3 Ma).
The appearance of Aulopiformes in the fossil record is marked by isolated teeth of indeterminate Alepisauroidei from the Blesa Formation near Alcaine (Spain) [74, 75]. Owing to the presence of charophytes in the fossil-bearing layers, these can be associated with the *Atopochara trivolvis triquetra* biozone [74], indicating a Barremian age [76, 77]. The presence of Aulopiformes in the Barremian is further supported by †*Atolvorator longipectoralis* from the Morro do Chaves Formation at São Miguel dos Campos (Sergipe-Alagoas Basin, Brazil) [75, 78, 79], for which a late Barremian age is suggested by palynomorphs and pollen occurrences [80]. Given that the age of Alepisauroidei indet. could be early or late Barremian, the fossils date to 130.8-126.3 Ma. In our phylogenomic data set, Aulopiformes are represented by *Parasudis fraserbrunneri*.

Myctophiformes: †*Sardinioides* spp. van der Marck, 1858 (81.3–80.7 Ma).
A large number of Cretaceous fossil from Germany and Lebanon have been assigned to the order Myctophiformes, but the taxonomic position of many of these remains ambiguous [81]. Among the oldest of these are fossils from the Coesfeld Member at Sendenhorst (Münster Basin, Germany) that were originally described under the name †*Osmeroides monasteri* and later assigned to the genus †*Sardinioides*. This genus is robustly placed among Myctophiformes in cladistic analyses [82]. Based on its ammonite fauna, the Coesfeld Member can be constrained to an age between the top of the †*Scaphites hippocrepis* III Zone and the base of the †*Baculites maclearni* Zone [83], and thus to 81.3–80.7 Ma [68]. Myctophiformes are represented in our taxon set by *Benthosema glaciale*.

Acanthomorphata: †*Pseudomonocentris microspinosus* González-Rodríguez, Schultze, and Arratia, 2013, †*Handuichthys interopercularis* González-Rodríguez, Schultze, and Arratia, 2013, †*Dalgoichthys tropicalis* González-Rodríguez, Schultze, and Arratia, 2013, and †*Muhichthys cordobai* González-Rodríguez and Fielitz, 2008, (106.8-98.0 Ma).
Acanthomorph fishes are abundant in the fossil record of the Cenomanian [3, 84], with an age of at least 98.0 Ma for fossils from the Sannine Limestone at Hajula (Lebanon) [3, 85]. These acanthomorphs, however, might be predated by recently described specimens of †*Pseudomonocentris microspinosus*, †*Handuichthys interopercularis*, †*Dalgoichthys tropicalis* [86], and †*Muhichthys cor-*

dobai [87] from the El Doctor Formation at the Muhi Quarry near Zimapán (Hidalgo, Mexico). The age of these deposits can be constrained roughly to Albanian-Cenomanian [88] and were interpreted as “very late Albian or very early Cenomanian” [89]. We here assume that the Muhi Quarry falls into the second half of the Albian or the first half of the Cenomanian, and that the acanthomorphs from this location thus date to 106.8-97.2 Ma. We adjust the lower boundary of this range to 98.0 Ma to account for the minimum age of the Hajula acanthomorphs [3]. In our phylogeny, Acanthomorphata are represented by a total of 76 taxa.

Polymixiiformes: †*Homonotichthys dorsalis* (Dixon, 1850), †*Homonotichthys rotundus* (Woodward, 1902), and †*Homonotichthys pulchellus* (Dixon, 1850) (97.1-94.0 Ma).
Following Malmstrøm et al.[90] and Rabosky et al.[91], we here consider fossils of the genus †*Homonotichthys* (rather than †*Berycopsis* or †*Omosoma* with uncertain taxonomic assignments) as the earliest records of Polymixiiformes. †*Homonotichthys dorsalis*, †*H. rotundus*, and †*H. pulchellus* are all recorded from the Cenomanian English Chalk of Kent and Sussex (United Kingdom) where they occur in the *Holoaster subglobosus* ammonite zone [92]. This traditional zone spans the *Acanthoceras rhotomagense* and *Neocardioceras juddii* modern zones [93, 94]. The top of the *Neocardioceras juddii* zone has a date of 94.0 Ma, the *Cunningtoniceras inerme* zone began about 3.0 myr earlier, and *Acanthoceras rhotomagense* is about 100 kyr younger than *Cunningtoniceras inerme* [68]; thus, the first occurrence of †*Homonotichthys* dates to 97.1-94.0 Ma. Our phylogenomic data set includes *Polymixia japonica* as the single representative of Polymixiiformes.

Lampriformes: †*Aipichthys minor* (Pictet, 1850) (100.5-98.0 Ma).
Lampriforms first appear in the Cenomanian, with several species being recorded from the limestones of Haqil and Hajula (Lebanon), the English Chalk of Sussex (United Kingdom), and the Sierra Madre Formation in Chiapas (Mexico) [85, 95]. The oldest of these localities is the Hajula Limestone for which a minimum age of 98.0 Ma can be given based on the presence of *Mantelliceras mantelli* in overlying layers [3]. The maximum age of the Hajula Limestone is the base of the Cenomanian at 100.5 Ma. †*Aipichthys minor* is recorded from Hajula and is consistently placed on the lampriform stem in phylogenetic analyses [94, 96, 97]. Lampriformes are represented in our phylogenomic data set by *Regalecus glesne* and *Lampris guttatus.*

Lampridae: †*Megalampris keyesi* Gottfried, Fordyce, and Rust, 2006, (27.8-25.2 Ma).
The earliest record of the family Lampridae is †*Megalampris keyesi* from the Otekaike Limestone near Tokarahi (North Otago, New Zealand) [98]. The lower glauconitic portion of the Otekaike Limestone, from which the specimen was recorded, has a New Zealand Duntroonian age [98]. The Duntroonian is roughly equivalent to the Chattian [99] with a minimum age of 25.2 Ma determined by isotope stratigraphy [100], thus constraining the age of †*Megalampris keyesi* to 27.8-25.2 Ma. Our phylogenomic data set includes *Lampris guttatus* as a single representative of Lampridae.

Paracanthopterygii: †*Xenyllion stewarti* Newbrey, Murray, Wilson, Brinkmann, and Neumann, 2013 (98.2-97.8 Ma).
Members of the extinct family Sphenocephalidae have long been regarded as the earliest representatives of Paracanthopterygii [8, 101, 102], and despite repeated changes to the composition of Paracanthopterygii, this placement of Sphenocephalidae has recently been corroborated by phyloge-

netic analyses [103]. The earliest record of Sphenocephalidae is †*Xenyllion stewarti* from the Mowry Formation near Vernal (Utah, USA) [104], occurring in the *Neogastroplites americanus* ammonite zone [105] with an age of 98.2-97.8 Ma [68]. Our phylogenomic data set includes six representatives of Paracanthopterygii.

Percopsiformes: †*Mcconichthys longipinnis* Grande, 1988 (66.0-63.6 Ma).
The earliest record of Percopsiformes is provided by †*Mcconichthys longipinnis* from the Tullock Formation in McCone County (Montana, USA) [8, 89, 106, 107]. The Tullock Formation is associated with the Puercan North American Land Mammal Age and its base approximates the Cretaceous-Paleogene boundary [108]. The top of the Tullock Formation is younger than the C28r/C28n magnetochron boundary at 63.6 Ma [109]; thus, †*Mcconichthys longipinnis* is dated to 66.0-63.6 Ma [110]. Our phylogenomic data set includes two members of Percopsiformes, *Percopsis transmontana* and *Typhlichthys subterraneus.*

Percopsidae: †*Massamorichthys wilsoni* Murray, 1996 and †*Lateopisciculus turrifumosus* Murray and Wilson, 1996 (62.2-59.9 Ma).
The earliest known occurrences of Percopsidae are fossils assigned to †*Massamorichthys wilsoni* and †*Lateopisciculus turrifumosus* from the Paskapoo Formation (Alberta, Canada) [106, 111]. Of these, †*Massamorichthys wilsoni* is known from a vast number of fossils at the same locality as the osmeriform †*Speirsaenigma lindoei* (see above); thus, they share the age of 60.7-59.9 Ma. The age estimate of †*Lateopisciculus turrifumosus* is less precise. The species is known from the Smoky Tower locality that can only be constrained to the Tiffanian (NALMA) [111] and might thus be as old as 62.2 Ma [52]. The first occurrence of Percopsidae thus dates to 62.2-59.9 Ma. The family is represented in our phylogenomic data set by *Percopsis transmontana.*

Zeiformes: †*Cretazeus rinaldii* Tyler et al., 2000 (89.8-83.0 Ma).
The earliest zeiform record is †*Cretazeus rinaldii* from the Calcari di Melissano at Canale near Nardò (Italy) [112]. While the Calcari di Melissano were long considered to be Upper Campanian in age [113], newer studies support an older age of the formation between the Coniacian and the basal Campanian, with a minimum age around 83 Ma [114, 115]. Thus, we here interpret the first occurrence age of Zeariae as 89.8-83.0 Ma. Zeiformes are represented in our data set by *Zeus faber* and *Cyttopsis rosea.*

Gadiformes: †“*Protocodus*” sp. (63.3-59.7 Ma).
†“*Protocodus*” sp., known from a nearly complete specimen, is commonly considered the earliest skeletal fossil record of Gadiformes [101, 116–120] even though it has never been formally published. According to Rosenkrantz[116], the specimen was found in the Thyasira member of the Kangilia Formation (West Greenland), which is now considered part of the Eqalulik Formation [121]. Based on dinocyst and nannoplankton occurrence, the formation has been related to the NP4-NP5 zones with an age of 63.3-59.5 Ma [52]. In addition, 40Ar/39Ar age determination of volcanic rocks overlying the formation provides a minimum age of 60.7 $\pm$ 1.0 Ma [121]; thus, the age of first occurrence of Gadiformes can be constrained to 63.3-59.7 Ma. In our phylogenomic data set, Gadiformes are represented by *Gadus morhua.*

Acanthopterygii: †*Cryptoberyx minimus* Gaudant, 1978, †*Stichocentrus liratus* Patterson, 1967, †*Stichocentrus elegans* Gaudant, 1969, †*Stichocentrus spinulosus* Gayet, 1980, †*Stichopteryx lewisi* (Davis, 1887), †*Lissoberyx dayi* (Woodward, 1942), †*Lissoberyx arambourgi* Gaudant, 1969, †*Lissoberyx denticulatus* Gayet, 1980, †*Microcapros libanicus* Gayet, 1980, †*Libanoberyx spinosus* Gayet, 1980, and †*Hgulichthys spinus* Otero et al., 1995 (100.5-98.0 Ma).
The appearance of Acanthopterygii in the fossil record is marked by various beryciforms, trachichthyiforms, holocentriforms, and a single possible tetraodontiform from the Cenomanian deposits of Lebanon, Israel, Morocco, and the United Kingdom (see discussion in[90]). The oldest of these deposits is the Sannine Limestone at Hajula (Lebanon) [3] with an age of 100.5-98.0 Ma (see above). The species †*Cryptoberyx minimus*, †*Stichocentrus liratus*, †*Stichopteryx lewisi*, †*Lissoberyx dayi*, †*Lissoberyx arambourgi*, †*Lissoberyx denticulatus*, †*Microcapros libanicus*, †*Libanoberyx spinosus*, and †*Hgulichthys spinus* are all known from Hajula [85] and thus jointly represent the earliest record of Acanthopterygii. Our phylogenomic data set includes 67 representatives of Acanthopterygii.

Trachichthyiformes: †*Lissoberyx dayi* (Woodward, 1942), †*Lissoberyx arambourgi* Gaudant, 1969, †*Lissoberyx denticulatus* Gayet, 1980, †*Microcapros libanicus* Gayet, 1980, †*Libanoberyx spinosus* Gayet, 1980, and †*Hgulichthys spinus* Otero et al., 1995 (100.5-98.0 Ma).
Of the oldest members of Acanthopterygii from Hajula, several species have been placed in Trachichthyidae (now elevated to the order Trachichthyiformes): †*Lissoberyx dayi*, †*Lissoberyx arambourgi*, †*Lissoberyx denticulatus*, †*Microcapros libanicus*, †*Libanoberyx spinosus*, and †*Hgulichthys spinus* [85, 122–124]. These fossils all date to 100.5-98.0 Ma (see above). Our phylogenomic data set includes two representatives of Trachichthyiformes; *Monocentris japonica* and *Gephyroberyx darwini*.

Berycoidei: †*Berycomorus firdoussii* (Arambourg, 1967) (47.8-33.9 Ma).
The only known skeletal fossil record of Berycoidei is †*Berycomorus firdoussii* from the Pabdeh Formation at Ilam (Zagros Basin, Iran) [124]. The formation was erroneously assumed to be Rupelian in age by Arambourg[125] and has more recently been assigned a Middle to Late Eocene age [126]; thus, it dates to 47.8-33.9 Ma. Our phylogenomic data set includes *Beryx splendens* as a single representative of Berycoidei.

Stephanoberycoidei: †*Miobarbourisia aomori* Fujii, Uyeno, and Shimaguchi, 2007 (13.8-11.6 Ma), or Stephanoberycoidei indet. (56.0-47.8 Ma)
Even though several fossil species have been assigned to Stephanoberycoidei, most of them remain poorly known and their placement may not be reliable. Following Rabosky et al.[91], †*Miobarbourisia aomori* from Wadagawa Formation near Aomori City (Japan) [127] is here accepted as a member of Stephanoberycoidei. The Wadagawa Formation correlates to the middle part of the Californian Monterey Formation [128] and can be considered late Middle Miocene in age; thus 13.8-11.6 Ma. Further fossils assigned to Stephanoberycoidei have been reported from Upper Miocene deposits of California (USA) [129] and Italy [130]. Substantially older than †*Miobarbourisia aomori* is a '*Rondeletia*-like cetomimiform' from the Fur Formation of Jutland (Denmark) [131]. This 'cetomimiform' fossil has never been formally described but a preliminary examination by Friedman[124] showed

similarities with some stephanoberycoids. The Danish Fur Formation is considered Ypresian in age [131] and thus dates to 56.0-47.8 Ma. We here consider both †*Miobarbourisia aomori* and the fossil from the Danish Fur Formation as equally probable first occurrences of Stephanoberycoidei (weights: 0.5/0.5). The suborder is represented in our phylogenomic data set by *Acanthochaenus luetkenii*.

Holocentriformes: †*Stichocentrus liratus* Patterson, 1967, †*Stichocentrus elegans* Gaudant, 1969, †*Stichocentrus spinulosus* Gayet, 1980 (100.5-98.0 Ma).
Of the earliest members of Acanthopterygii from Hajula, those assigned to genus †*Stichocentrus* are generally considered holocentroids (and therefore holocentriforms) [8, 85, 124, 132] and have repeatedly been accepted as the earliest records of the group [8, 91]. These fossils date to 100.5-98.0 Ma (see above). Our phylogenomic data set includes the two holocentriforms *Myripristis jacobus* and *Holocentrus rufus*.

Percomorphaceae: †*Cretatriacanthus guidottii* Tyler and Sorbini, 1996 (89.8-83.0 Ma), or †*Plectocretacicus clarae* Sorbini, 1979 (100.5-98.0 Ma).
Cretaceous armoured acanthomorphs of the extinct superfamily Plectocretacicoidea have frequently been considered the earliest records of tetraodontiform fishes [2, 16, 17, 133, 134], a placement that, if correct, would also make them the earliest percomorphs. Four species of plectocretacicoids are currently known. The oldest of these is †*Plectocretacicus clarae* from the limestone of Haqil for which an age of 100.3-98.0 Ma can be given due to the cooccurrence of *Mantelliceras mantelli* [28]. While the monophyly of Plectocretacicoidea and their position as a stem group of Tetraodontiformes has been supported by phylogenetic analyses [2, 17], both have become increasingly questioned in recent years, following a preliminary reexamination of the fossil material [4] and the discovery of similar armoured acanthomorphs that were placed outside crown-group of percomorphs [86]. The phylogenetic position of the older plectocretacicoids is therefore currently unclear, with some authors accepting the tetraodontiform affiliation [10, 17] and others arguing that at least †*Plectocretacicus clarae* and †*Protriacanthus gortanii* should not be considered part of Tetraodontiformes [3–6, 8, 115]. In the latter case, the earliest record of Tetraodontiformes would be †*Cretatriacanthus guidottii* from the Calcari di Melissano at Canale near Nardò (Italy), exhibiting more tetraodontiform synapomorphies than the other plectocretacicoids [3, 5, 6, 115, 135]. †*Cretatriacanthus guidottii* thus has the same age as the zeiform †*Cretazeus rinaldii*; 89.8-83.0 Ma (see above). Following the group of authors questioning the tetraodontiform placement of †*Plectocretacicus clarae*, we here consider †*Cretatriacanthus guidottii* as a more probable first occurrence of Tetraodontiformes. However, after accounting for a possibility that †*Plectocretacicus clarae* is not a tetraodontiform but still a percomorph, we consider both taxa as equally probable first occurrences of Percomorphaceae (weights: 0.5/0.5). Our phylogenomic data set includes 61 representatives of Percomorphaceae.

Ophidiaria: †*Pastorius methenyi* Carnevale and Johnson, 2015 (76.4-69.8 Ma).
†*Pastorius methenyi* from the Liburnica Formation near Trebiciano (Italy) has recently been described as the oldest member of Ophidiiformes [136, 137], taking that title from the Eocene †*Eolamprologus senectus*. The fossil-bearing layers at Trebiciano have a Late Campanian or Early Maastrichtian age [136, 138] and thus date to 76.4-69.8 Ma. With *Brotula barbata* and *Carapus acus*, two members of

Ophidiaria are included in our phylogenomic data set.

Batrachoidaria: †*Louckaichthys novosadi* Přikryl and Carnevale, 2017 (32.0-29.6 Ma).
The fossil record of Batrachoidiformes has recently been extended to the Oligocene, with the description of †*Louckaichthys novosadi* from the Menelitic Formation at the Loučka locality near Valašské Meziříčí (Czech Republic) [139] that now represents the first occurrence of the order. The Dynów Member of the Menilitic Formation, from which the fossil was collected, is classified into the Rupelian nannoplancton zone NP23 [140], and thus dates to 32.0-29.6 Ma. Our phylogenomic data set includes a single representative of Batrachoidaria, *Chatrabus melanurus*.

Gobiaria: †*Carlomonnius quasigobius* Bannikov and Carnevale, 2016 (49.2-48.9 Ma).
The first gobiiform specimen was recently identified from the Calcari nummulitici at Monte Bolca near Verona (Italy) and described as †*Carlomonnius quasigobius* [141]. The age of the fossil-bearing layers at Monte Bolca can be classified into nannoplancton zone NP14 based on the presence of *Discoaster sublodoensis* [3, 142]. Larger foraminiferal assemblages of *Alveolina cremae*, *A. rugosa*, *A. distefanoi*, and *A. rutimeyeri* further allow the constraint to to Shallow Benthic Zone SBZ11 [143]. The top of SBZ11 (48.9 Ma) is just above the base of NP14 (49.2 Ma); thus, the age of the Monte Bolca deposits dates to 49.2-48.9 Ma. Gobiaria are represented in our phylogenomic data set by *Lesueurigobius* cf. *sanzoi* and *Periophthalmodon schlosseri*.

***Hippocampus*:** *Hippocampus* †*sarmaticus* and *Hippocampus* †*slovenicus* Žalohar, Hitij, and Križnar, 2009 (12.2-11.3 Ma).
The genus *Hippocampus* first appears in the Miocene with the two species *Hippocampus* †*sarmaticus* and *H.* †*slovenicus* from the Coprolitic Horizon at the Tunjice Hills (Slovenia) [144, 145]. Based on cooccurring diatomaceous microflora, the Coprolitic Horizon can be assigned to the Lower Sarmatian Central Paratethys age which can be constrained to 12.2-11.3 Ma [146, 147]. The genus *Hippocampus* is represented in our phylogenomic data set by *Hippocampus comes*. The genus *Syngnathus* is more closely related to other genera than to *Hippocampus* [145]; therefore, we don't constrain the age of *Syngnathus* directly.

Tetraodontiformes: †*Cretatriacanthus guidottii* Tyler and Sorbini, 1996 (89.8-83.0 Ma), or †*Plectocretacicus clarae* Sorbini, 1979 (100.5-98.0 Ma).
We here consider †*Cretatriacanthus guidottii*, with an age of 89.8-83.0 Ma, as a more probable first record of Tetraodontiformes than the older †*Plectocretacicus clarae* (weights: 0.67/0.33) (see above). Tetraodontiformes are represented in our phylogenomic data set by three species.

Lophiiformes: †*Eophryne barbutii* Carnevale and Pietsch, 2009, †*Sharfia mirabilis* Pietsch and Carnevale, 2011, †*Tarkus squirei* Carnevale and Pietsch, 2011, and †*Caruso brachysomus* Carnevale and Pietsch, 2012 (49.2-48.9 Ma).
A number of lophiiform families appear simultaneously in the fossil record of the Eocene, with †*Eophryne barbutii* [148], †*Sharfia mirabilis* [149], †*Tarkus squirei* [150], and †*Caruso brachysomus* [151] all occurring in the Calcari nummulitici at Monte Bolca (Italy). The first occurrence of Lophiiformes therefore dates to 49.2-48.9 Ma (see above). Our phylogenomic data set includes a single representative of Lophiiformes, *Antennarius striatus*.

Morone: †*Morone aequalis* (Weiler, 1942) (20.4-16.0 Ma).
The genus *Morone* is represented in the fossil record by †*Morone aequalis* from the Frankfurt Formation (former Upper Hydrobia Beds) at Frankfurt (Germany) [152]. The layer producing the fossil is part of the lowermost Frankfurt Formation [153]. The Frankfurt Formation is considered Burdigalian in age [154] and thus dates to 20.4-16.0 Ma. The genus *Morone* is represented in our phylogenomic data set by *Morone saxatilis*.

Channoidei: †*Eochanna chorlakkiensis* Roe, 1991, and †*Anchichanna kuldanensis* Murray and Thewissen, 2008 (48.9-37.8 Ma).
The earliest records of Channoidei are provided by two species of channids from the Kuldana Formation in the Kohat District of Pakistan: †*Eochanna chorlakkiensis* [155] was collected near Chorlakki and †*Anchichanna kuldanensis* [156] was found in the Ganda Kas area of the Kala Chitta Hills; the two sites are about 30 km apart. The Kuldana Formation is reported as early to middle Eocene in age [155, 156] but no older than SBZ12 [157]. Thus, the earliest record of Channoidei dates to 48.9-37.8 Ma. In our phylogenomic data set, Channoidei are represented by *Channa argus*.

Anabantoidei: *Osphronemus goramy* Lacepède, 1801, and †*Ombilinichthys yamini* Murray, Zaim, Rizal, Aswan, Gunnell, and Ciochon, 2015 (37.8-23.0 Ma)
The only two fossils known of Anabantoidei have both been reported from the Sangkarewang Formation in the Ombilin Basin (West Sumatra, Indonesia) and have been assigned to the extant species *Osphronemus goramy* and the extinct †*Ombilinichthys yamini* [158, 159]. The age of the Sangkarewang Formation is very poorly known and various authors have considered it Cretaceous, Paleogene, or Miocene. Reviewing the available evidence, Murray et al.[159] concluded that "a late Eocene or Oligocene age is not unreasonable". Following this assessment, we here assume an age of 37.8-23.0 Ma for fossils from the Sangkarewang Formation. Anabantoidei are represented in our phylogenomic data set by *Anabas testudineus* and *Helostoma temminckii*.

Aplocheiloidei: †*Kenyaichthys kipkechi* Altner and Reichenbacher, 2015 (6.0-5.7 Ma).
The only known fossil record of Aplocheiloidei is the recently described †*Kenyaichthys kipkechi* from the Lukeino Formation in the Tugen Hills (Central Rift Valley, Kenya) [160]. The Lukeino Formation is late Miocene in age and has been constrained to 6.0-5.7 Ma [161, 162]. Our phylogenomic data set includes two representatives of Aplocheiloidei; *Kryptolebias marmoratus* and *Nothobranchius furzeri*.

Cyprinodontoidei: †*Prolebias stenoura* Sauvage, 1874, †*Francolebias aymardi* (Sauvage, 1869), and †*Francolebias delphinensis* (Gaudant, 1989) (33.9-28.1 Ma), or Poeciliidae *indet.* (56.0-47.8 Ma).
Undescribed poeciliid fossils have been reported from the Maíz Gordo Formation near Salta (Argentina) [163]. The Maíz Gordo Formation can be assigned to the Riochican or the early Casamayoran South American Land Mammal Ages (SALMA), suggesting an Ypresian age [164, 165]. However, the taxonomic assignment of these undescribed fossils is questionable. No poeciliid fossils were included in Cione and Báez'[166] list of paleocene fishes from Argentina, and according to López-Fernández and Albert[167], the only confirmed fossils of poeciliids in Argentina are of middle-late Miocene age. No other Eocene fossils are known of Cyprinodontoidei. The earliest records of

Cyprinodontoidei outside of South America are †*Prolebias stenoura* from the deposits at Corent (Puy-de-Dôme, France), †*Francolebias aymardi* from the Calcaires marneux of Ronzon (Haute-Loire, France) and †*Francolebias delphinensis* from sediments near Montbrun-les Bains (Drôme, France) [160, 168–170], all of which have a lower Oligocene age. Thus, the first occurrence of Cyprinodontoidei dates to 56.0-47.8 Ma if the taxonomic assignment of Poecilidae *indet.* is correct, or to 33.9-28.1 Ma if it is not. We assume equal probabilities for both ages (weights: 0.5/0.5). Our phylogenomic data set includes four representatives of Cyprinodontoidei.

Cichlinae: *Gymnogeophagus* †*eocenicus* Malabarba, Malabarba, and del Papa, 2010, †*Plesioheros chauliodus* Alano Perez, Malabarba, and del Papa, 2010, and †*Proterocara argentina* Malabarba, Zuleta, and del Papa, 2006 (45.0-40.0 Ma).
Gymnogeophagus †*eocenicus*, †*Plesioheros chauliodus*, and †*Proterocara argentina* from the Lumbrera Formation of the Salta Province (Argentina) have been placed within the crown of Cichlinae [171–173]. The Lumbrera Formation has traditionally been assigned to the Casamayoran (SALMA) [174] and direct dating of a tuff layer near the top of the formation provides a minimum age of 39.9 Ma [174]. The age of fossils can therefore be assumed to be 45.0-40.0 Ma [175]. Our phylogenomic data set includes seven species representative of Cichlinae.

Heroini: †*Plesioheros chauliodus* Alano Perez, Malabarba, and del Papa, 2010 (45.0-40.0 Ma).
Of the fossils known from the Lumbrera Formation, †*Plesioheros chauliodus* can be assigned to the tribe Heroini [172]; thus, this earliest occurrence of this tribe dates to 45.0-40.0 Ma. Our phylogenomic data set includes three representatives of Heroini. Note that we do not constrain the age of Geophagini because their probable sister lineage, the tribe Chaetobranchini (possible together with Astronotini) [18, 171, 176, 177], is not included in the data set.

Cichlasomatini: †*Tremembichthys garciae* Malabarba and Malabarba, 2008 (35.0-30.0 Ma)
Of three fossils that have been assigned to Cichlasomatini, the oldest is †*Tremembichthys garciae* from the Entre-Córregos Formation near Aiuruoca (Minas Gerais, Brazil) [178, 179]. Palynomorphs at the base of the Entre-Córregos Formation indicate an age near the Eocene-Oligocene boundary, around 35.0-30.0 Ma [180, 181]. Cichlasomatini are represented in our phylogenomic data set by three species.

Pseudocrenilabrinae: †*Mahengechromis* spp. Murray, 2000 (46.0-45.0 Ma).
The earliest record of Pseudocrenilabrinae is provided by specimens from the Mahenge paleolake (Tanzania) assigned to genus †*Mahengechromis* [182]. U/Pb isotopic analyses of Mahenge zircon constrain the age of the paleolake sediments to 46.0-45.0 Ma [175, 183]. Our phylogenomic data set includes eight representatives of Pseudocrenilabrinae.

Heterochromini: ?*Heterochromis* sp. (33.9-16.0 Ma).
Three fossil specimens from the Ad Darb Formation (Tihamat Asir, Saudi Arabia) have been considered closely related to genus *Heterochromis* on the basis of predorsal bone count and scale structure [184, 185]. The Ad Darb Formation is assumed to be either Oligocene [184] or early Miocene [186] in age and therefore dates to 33.9-16.0 Ma. Our phylogenomic data set includes the single extant species of Heterochromini, *Heterochromis multidens*.

Unnamed clade: †*Mahengechromis* spp. Murray, 2000 (46.0-45.0 Ma).
Even though the clade comprising all tribes of African cichlids except Heterochromini and Tylochromini has not been named, it is strongly supported by phylogenetic analyses [18, 187, 188] and morphologically recognizable by having only a single predorsal bone [189]; therefore, the clade can (and should be[18]) used for time calibration with the CladeAge approach. A position of †*Mahengechromis* within this clade is supported by its single predorsal bone [185, 190, 191]. The unnamed clade is represented in our phylogenomic data set by six species.

Haplotilapiini *sensu* Schliewen and Stiassny, 2003: *Oreochromis* †*kabchorensis* Penk et al., 2019 (12.5-12.0 Ma).
Phylogenetic analyses strongly support the monophyly of Haplotilapiini as well as a sister-group relationship with either Hemichromini or a clade combining Hemichromini and Pelmatochromini [18, 187, 188, 192]. Thus, the sister group of Haplotilapiini is included in our data set. Haplotilapiini are morphologically recognizable by having tricuspid teeth in the inner row of their dentition [193]. The oldest known record of Haplotilapiini is provided by specimens from Ngorora fish Lagerstätte at Kabchore (Tugen Hills, Central Kenya) [194]. The Kabchore locality has been assigned to Member C of the Ngorora Formation with an age of 12.5-12.0 Ma [195]. Our phylogenomic data set includes three representatives of Haplotilapiini.

Oreochromini: *Oreochromis* †*kabchorensis* Penk et al., 2019 (12.5-12.0 Ma).
Given that the earliest records of Haplotilapiini have been placed in genus *Oreochromis* (see above), they also provide a minimum age for Oreochromini of 12.5-12.0 Ma. Oreochromini are represented in our phylogenomic data set by *Oreochromis niloticus*.

Supplementary Figures

Supplementary Figure 1: Species tree inferred with the concatenated "permissive" set of gene alignments. Node values indicate Bayesian posterior probabilities (BPP) estimated with BEAST 2.

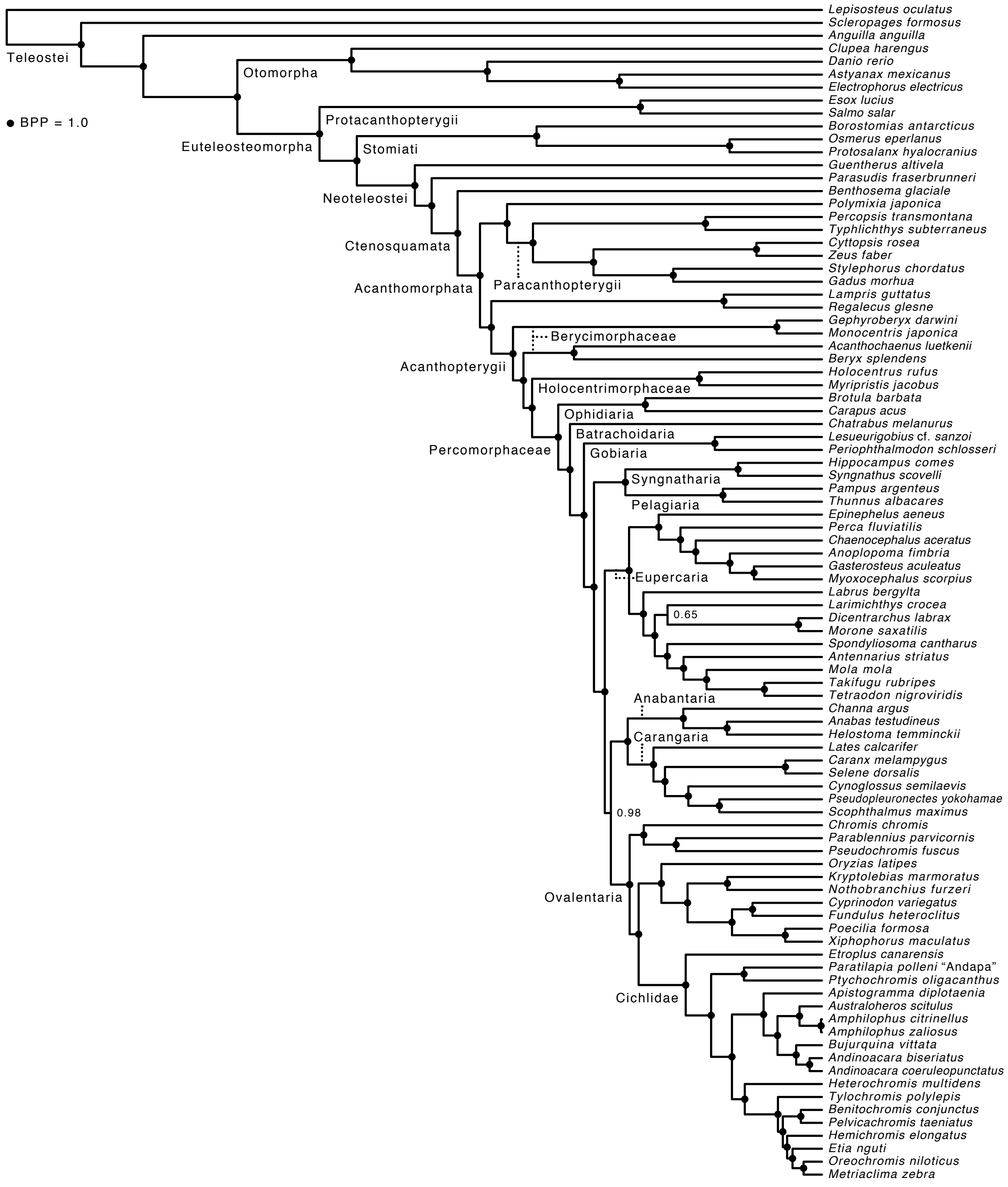

Supplementary Figure 2: Species tree inferred with the concatenated "strict" set of gene alignments. Node values indicate Bayesian posterior probabilities (BPP) estimated with BEAST 2.

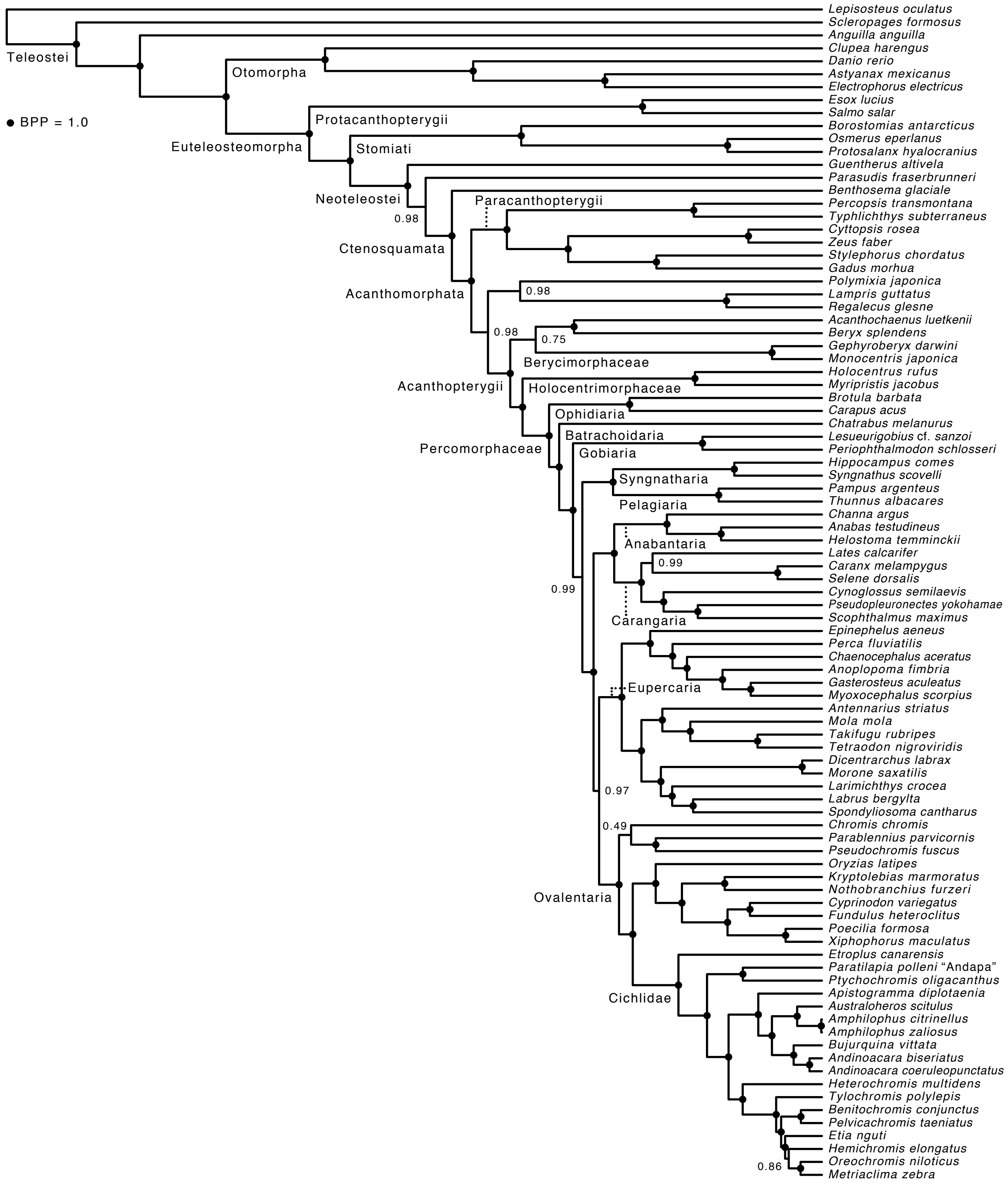

Supplementary Figure 3: Species tree inferred with the multi-species coalescent model and the "permissive" set of genes. Node values indicate Bootstrap support (BS) estimated with ASTRAL.

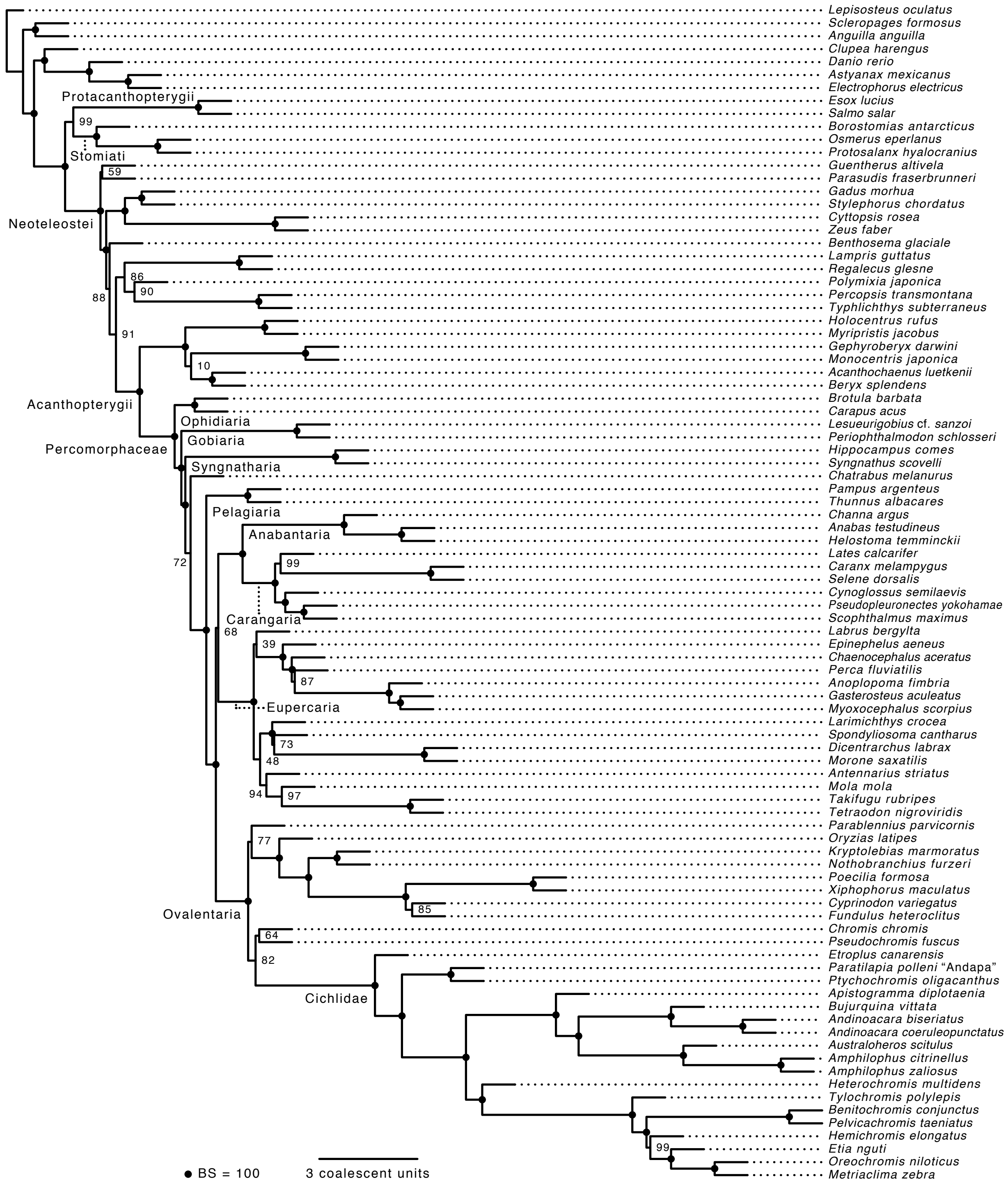

Supplementary Figure 4: Species tree inferred with the multi-species coalescent model and the "strict" set of genes. Node values indicate Bootstrap support (BS) estimated with ASTRAL.

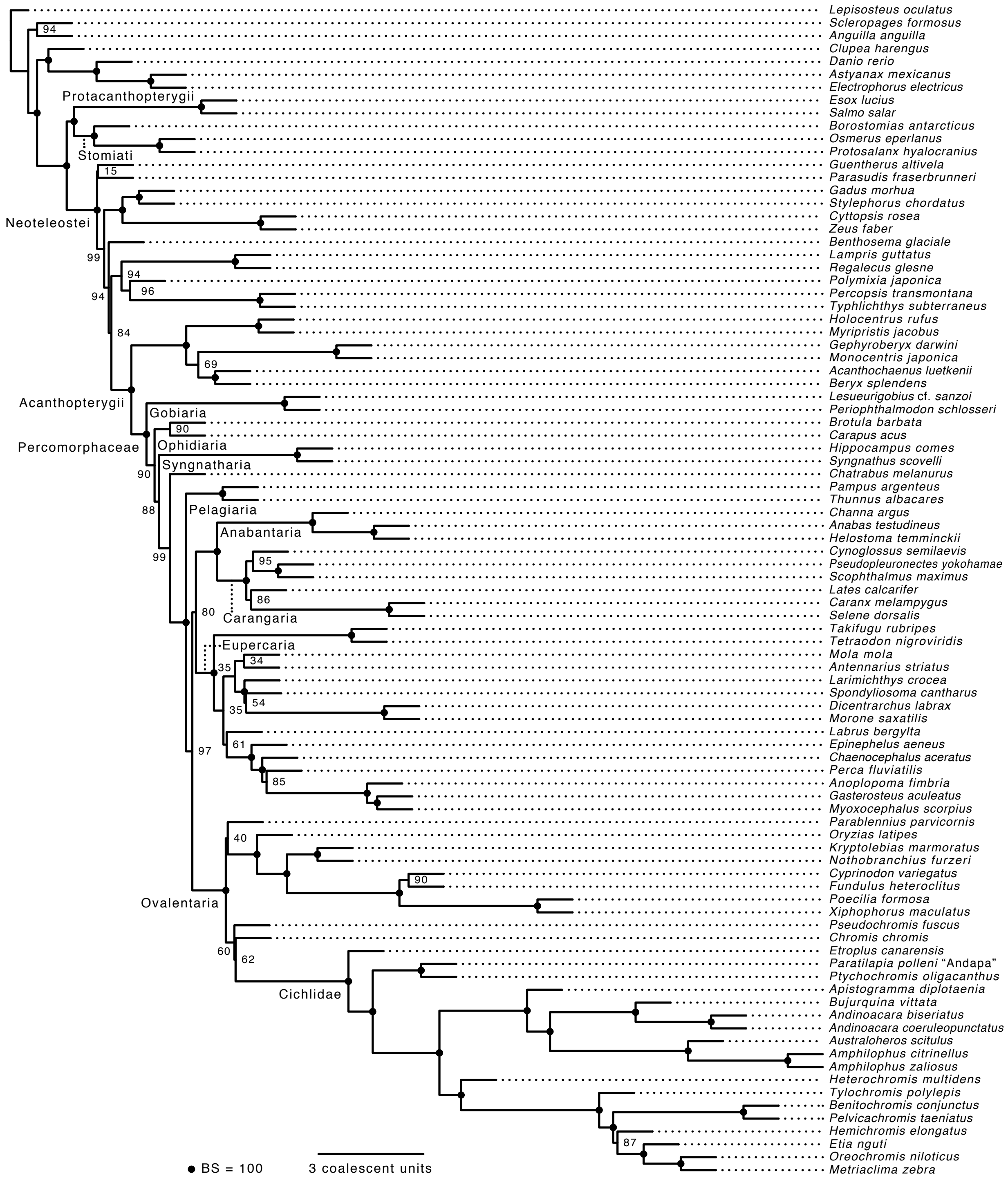

Supplementary Figure 5: Convergence tests with two possible first occurrences of a clade. The ages of two simulated fossils (each with an uncertainty of 10 Myr) are shown in purple (younger fossil) and orange (older fossil), with temporal distances between the fossils of 30 (A, E, I), 60 (B, F, J), 90 (C, G, K), and 120 Myr (D, H, L). The calibration densities $f_{\text{younger}}(t)$ (purple), $f_{\text{older}}(t)$ (orange), and $f(t)$ (grey) are shown in A-D, corresponding posterior densities are shown in E-H, and the MCMC traces for the age of the calibrated node, sampled in every 100th iteration, are shown in I-L.

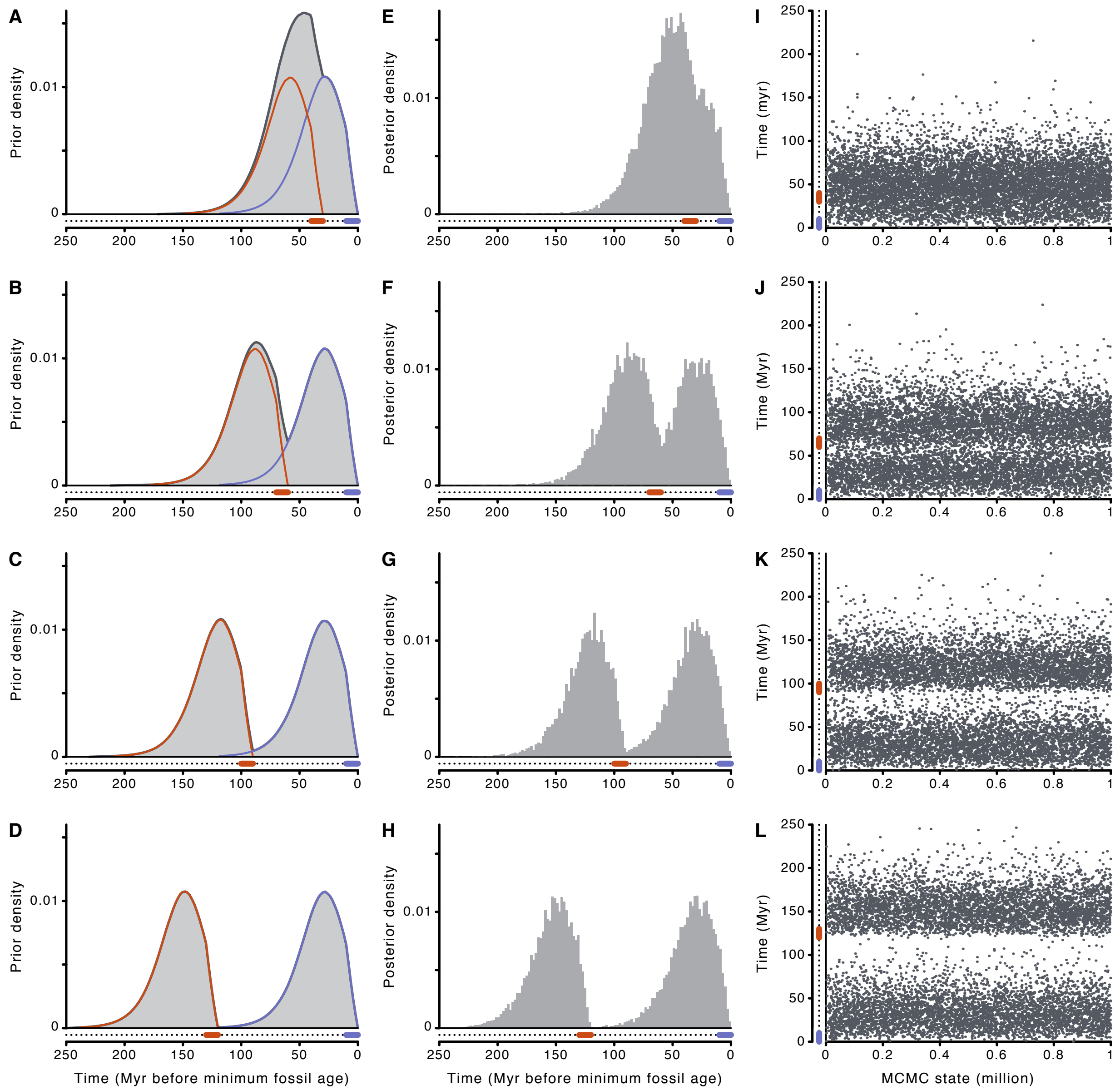

Supplementary Figure 6: Divergence times inferred with the "permissive" set of genes. Grey node bars indicate 95% highest-posterior density (HPD) intervals for node ages.

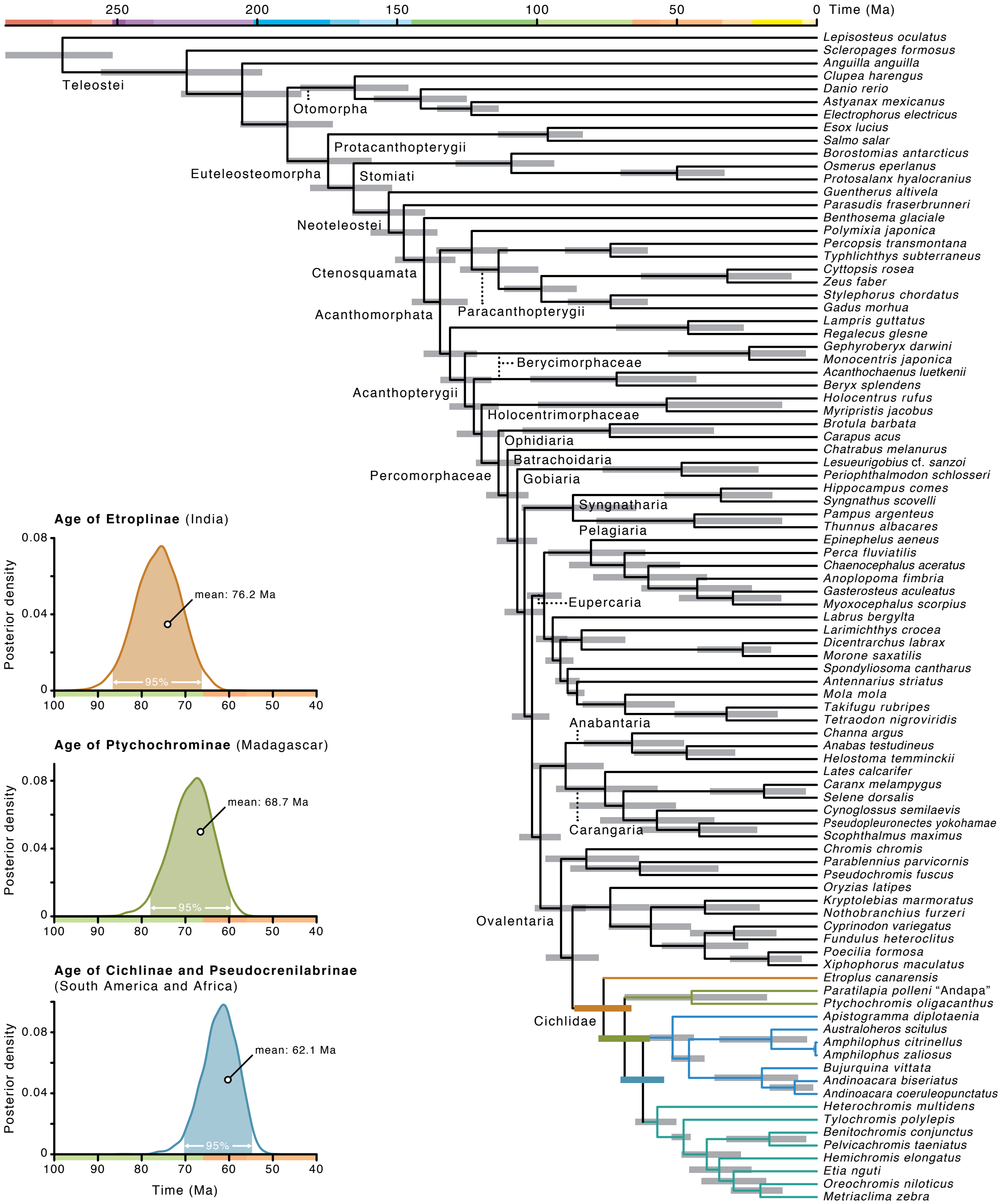

Supplementary Figure 7: Divergence times inferred with the "strict" set of genes. Grey node bars indicate 95% HPD intervals for node ages.

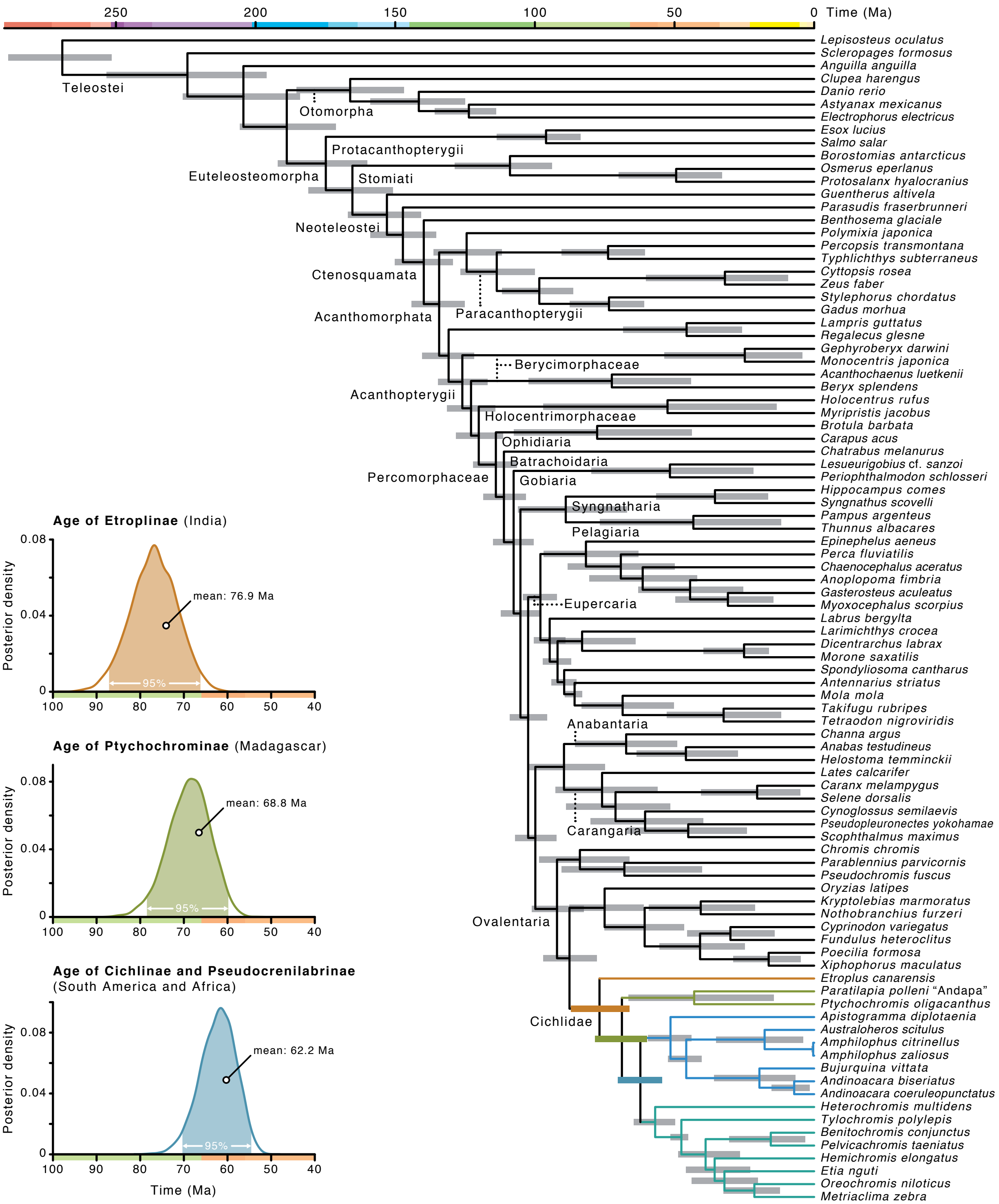

Supplementary Tables

Supplementary Table 1: Cichlid species sampled for whole-genome sequencing.
Samples with source information "Own collection" were obtained by members of the Salzburger lab during field trips to Cameroon and Zambia.

Species	Voucher	Source	Country of origin	Coverage
Etroplus canarensis	JWD4	Aquarium trade	India	19.01
Paratilapia polleni "Andapa"	JWD5	Zoo Zurich	Madagascar	23.12
Ptychochromis oligacanthus	FPO1	Zoo Brussels	Madagascar	7.25
Apistogramma diplotaenia	JWD9	Z. Musilová	Venezuela	8.23
Australoheros scitulus	JWE2	Aquarium trade	Uruguay	10.48
Amphilophus zaliosus	JWE1	M. Barluenga	Nicaragua	8.34
Bujurquina vittata	JWD8	Z. Musilová	Paraguay	9.17
Andinoacara biseriatus	JWD7	Z. Musilová	Ecuador	8.98
Heterochromis multidens	JWF1	Own collection	Cameroon	22.25
Tylochromis polylepis	JAH5	Own collection	Zambia	17.53
Benitochromis conjunctus	JWE4	Own collection	Cameroon	9.20
Pelvicachromis taeniatus	JWI4	Own collection	Cameroon	9.51
Hemichromis elongatus	JWE9	Own collection	Cameroon	23.07
Etia nguti	JWD3	Own collection	Cameroon	17.70

Supplementary Table 2: Whole-genome assemblies generated for this study.
The results of the BUSCO analysis are given in the order complete (c), complete and single copy (c+s), complete and duplicated (c+d), fragmented (f), missing (m). The BUSCO test library contained a total of 4,584 conserved actinopterygian genes.

Species	Assembly size	N50	— BUSCOs (c/c+s/c+d/f/m) —					Accession
Etroplus canarensis	975,424,675	18,345	3,338	3,258	80	714	532	SAMN12123891
Paratilapia polleni "Andapa"	814,091,157	21,259	3,549	3,477	72	636	399	SAMN12123892
Ptychochromis oligacanthus	707,176,575	8,314	2,499	2,464	35	1,185	900	SAMN12123633
Apistogramma diplotaenia	627,325,156	25,146	3,875	3,803	72	421	288	SAMN12123895
Australoheros scitulus	709,595,499	10,904	2,987	2,927	60	911	686	SAMN12123897
Amphilophus zaliosus	692,081,349	9,617	2,611	2,563	48	1,149	824	SAMN12123896
Bujurquina vittata	686,045,042	28,150	3,790	3,703	87	525	269	SAMN12123894
Andinoacara biseriatus	682,812,987	25,636	3,752	3,677	75	478	354	SAMN12123893
Heterochromis multidens	833,453,564	17,755	3,285	3,212	73	794	505	SAMN12123905
Tylochromis polylepis	744,326,816	18,818	3,528	3,455	73	634	422	SAMN12123821
Benitochromis conjunctus	665,812,357	27,758	3,722	3,636	86	520	342	SAMN12123899
Pelvicachromis taeniatus	693,375,608	26,667	3,812	3,731	81	464	308	SAMN12123930
Hemichromis elongatus	694,371,948	37,079	4,056	3,947	109	302	226	SAMN12123904
Etia nguti	737,212,856	33,462	3,968	3,866	102	372	244	SAMN12123890

Supplementary Table 3: Teleost species used for marker selection.
The 15 species listed here were selected out of 42 teleost species included in release 94 of the Ensembl database to represent diverse teleost lineages with a focus on cichlids and their closer relatives. Exon-sequence similarity was quantified between zebrafish (*Danio rerio*) and all other species.

Species	Family	Order	Group
Danio rerio	Danionidae	Cypriniformes	Section Otophysa
Esox lucius	Esocidae	Esociformes	Subcohort Protacanthopterygii
Gadus morhua	Gadidae	Gadiformes	Subseries Gadariae
Periophthalmus magnuspinnatus	Gobiidae	Gobiiformes	Series Gobiaria
Hippocampus comes	Syngnathidae	Syngnathiformes	Series Syngnatharia
Takifugu rubripes	Tetraodontidae	Tetraodontiformes	Series Eupercaria
Labrus bergylta	Labridae	Labriformes	Series Eupercaria
Gasterosteus aculeatus	Gasterosteidae	Perciformes	Series Eupercaria
Scophthalmus maximus	Scophthalmidae	Pleuronectiformes	Series Carangaria
Amphiprion percula	Pomacentridae	incertae sedis	Series Ovalentaria
Oryzias latipes	Adrianichthyidae	Beloniformes	Series Ovalentaria
Xiphophorus maculatus	Poeciliidae	Cyprinodontiformes	Series Ovalentaria
Amphilophus citrinellus	Cichlidae	Cichliformes	Series Ovalentaria
Oreochromis niloticus	Cichlidae	Cichliformes	Series Ovalentaria
Metriaclima zebra	Cichlidae	Cichliformes	Series Ovalentaria

Supplementary Table 4: Assembly files used for marker selection.
Assembly files for the 15 species (see Supplementary Table 3) listed here are part of Ensembl databank release 94.

Species	Assembly file
Danio rerio	Danio_rerio.GRCz11.dna.toplevel.fa.gz
Esox lucius	Esox_lucius.Eluc_V3.cdna.all.fa.gz
Gadus morhua	Gadus_morhua.gadMor1.cdna.all.fa.gz
Periophthalmus magnuspinnatus	Periophthalmus_magnuspinnatus.PM.fa.cdna.all.fa.gz
Hippocampus comes	Hippocampus_comes.H_comes_QL1_v1.cdna.all.fa.gz
Takifugu rubripes	Takifugu_rubripes.FUGU5.cdna.all.fa.gz
Labrus bergylta	Labrus_bergylta.BallGen_V1.cdna.all.fa.gz
Gasterosteus aculeatus	Gasterosteus_aculeatus.BROADS1.cdna.all.fa.gz
Scophthalmus maximus	Scophthalmus_maximus.ASM318616v1.cdna.all.fa.gz
Amphiprion percula	Amphiprion_percula.Nemo_v1.cdna.all.fa.gz
Oryzias latipes	Oryzias_latipes_hsok.ASM223469v1.cdna.all.fa.gz
Xiphophorus maculatus	Xiphophorus_maculatus.X_maculatus-5.0-male.cdna.all.fa.gz
Amphilophus citrinellus	Amphilophus_citrinellus.Midas_v5.cdna.all.fa.gz
Oreochromis niloticus	Oreochromis_niloticus.Orenil1.0.cdna.all.fa.gz
Metriaclima zebra	Maylandia_zebra.M_zebra_UMD2a.cdna.all.fa.gz

Supplementary Table 5: Targeted assemblies generated for this study.
Listed below are the percentages of exon sequences that could be assembled, relative to the most successful assembly. Targeted assembly with aTRAM was not performed for the three species that had the highest assembly success rates with Kollector.

Species	Kollector	aTRAM
Etroplus canarensis	2.7%	82.7%
Paratilapia polleni "Andapa"	11.4%	70.2%
Ptychochromis oligacanthus	4.3%	97.4%
Apistogramma diplotaenia	4.4%	100.0%
Australoheros scitulus	9.3%	84.9%
Amphilophus zaliosus	6.8%	89.8%
Bujurquina vittata	6.7%	92.6%
Andinoacara biseriatus	6.8%	90.4%
Heterochromis multidens	21.1%	78.3%
Tylochromis polylepis	71.1%	52.6%
Benitochromis conjunctus	64.0%	87.8%
Pelvicachromis taeniatus	77.4%	-
Hemichromis elongatus	100.0%	-
Etia nguti	76.2%	-

Supplementary Table 6: Teleost species used in phylogenetic analyses.

Species	Family	Order	Group
Lepisosteus oculatus	Lepisosteidae	Lepisosteiformes	Infraclass Holostei
Scleropages formosus	Osteoglossidae	Osteoglossiformes	Supercohort Osteoglossomorpha
Anguilla anguilla	Anguillidae	Anguilliformes	Cohort Elopomorpha
Clupea harengus	Clupeidae	Clupeiformes	Cohort Otomorpha
Danio rerio	Danionidae	Cypriniformes	Cohort Otomorpha
Astyanax mexicanus	Characidae	Characiformes	Cohort Otomorpha
Electrophorus electricus	Gymnotidae	Gymnotiformes	Cohort Otomorpha
Esox lucius	Esocidae	Esociformes	Subcohort Protacanthopterygii
Salmo salar	Salmonidae	Salmoniformes	Subcohort Protacanthopterygii
Borostomias antarcticus	Stomiidae	Stomiatiformes	Subcohort Stomiati
Osmerus eperlanus	Osmeridae	Osmeriformes	Subcohort Stomiati
Protosalanx hyalocranius	Salangidae	Osmeriformes	Subcohort Stomiati
Guentherus altivela	Ateleopodidae	Ateleopodiformes	Infracohort Ateleopodia
Parasudis fraserbrunneri	Chlorophthalmidae	Aulopiformes	Section Cyclosquamata
Benthosema glaciale	Myctophidae	Myctophiformes	Subsection Myctophata
Polymixia japonica	Polymixiidae	Polymixiiformes	Division Polymixiipterygii
Percopsis transmontana	Percopsidae	Percopsiformes	Division Paracanthopterygii
Typhlichthys subterraneus	Amblyopsidae	Percopsiformes	Division Paracanthopterygii
Cyttopsis rosea	Parazenidae	Zeiformes	Division Paracanthopterygii
Zeus faber	Zeidae	Zeiformes	Division Paracanthopterygii
Stylephorus chordatus	Stylephoridae	Stylephoriformes	Division Paracanthopterygii
Gadus morhua	Gadidae	Gadiformes	Division Paracanthopterygii
Lampris guttatus	Lampridae	Lampriformes	Division Lampripterygii
Regalecus glesne	Regalecidae	Lampriformes	Division Lampripterygii
Gephyroberyx darwini	Trachichthyidae	Trachichthyiformes	Subdivision Berycimorphaceae
Monocentris japonica	Monocentridae	Trachichthyiformes	Subdivision Berycimorphaceae
Acanthochaenus luetkenii	Stephanoberycidae	Beryciformes	Subdivision Berycimorphaceae
Beryx splendens	Berycidae	Beryciformes	Subdivision Berycimorphaceae
Holocentrus rufus	Holocentridae	Holocentriformes	Subdivision Holocentrimorphaceae
Myripristis jacobus	Holocentridae	Holocentriformes	Subdivision Holocentrimorphaceae
Brotula barbata	Ophidiidae	Ophidiiformes	Series Ophidiaria
Carapus acus	Ophidiidae	Ophidiiformes	Series Ophidiaria
Chatrabus melanurus	Batrachoididae	Batrachoidiformes	Series Batrachoidaria
Lesueurigobius cf. *sanzoi*	Gobiidae	Gobiiformes	Series Gobiaria
Periophthalmodon schlosseri	Oxudercidae	Gobiiformes	Series Gobiaria
Hippocampus comes	Syngnathidae	Syngnathiformes	Series Syngnatharia
Syngnathus scovelli	Syngnathidae	Syngnathiformes	Series Syngnatharia
Pampus argenteus	Stromateidae	Scombriformes	Series Pelagiaria
Thunnus albacares	Scombridae	Scombriformes	Series Pelagiaria
Epinephelus aeneus	Serranidae	Perciformes	Series Eupercaria
Perca fluviatilis	Percidae	Perciformes	Series Eupercaria
Chaenocephalus aceratus	Channichthyidae	Perciformes	Series Eupercaria
Anoplopoma fimbria	Anoplopomatidae	Perciformes	Series Eupercaria
Gasterosteus aculeatus	Gasterosteidae	Perciformes	Series Eupercaria
Myoxocephalus scorpius	Psychrolutidae	Perciformes	Series Eupercaria

Supplementary Table 6 (continued): Teleost species used in phylogenetic analyses.

Species	Family (Subfamily)	Order	Group
Labrus bergylta	Labridae	Labriformes	Series Eupercaria
Larimichthys crocea	Sciaenidae	incertae sedis	Series Eupercaria
Dicentrarchus labrax	Moronidae	incertae sedis	Series Eupercaria
Morone saxatilis	Moronidae	incertae sedis	Series Eupercaria
Spondyliosoma cantharus	Sparidae	Spariformes	Series Eupercaria
Antennarius striatus	Antennariidae	Lophiiformes	Series Eupercaria
Mola mola	Molidae	Tetraodontiformes	Series Eupercaria
Takifugu rubripes	Tetraodontidae	Tetraodontiformes	Series Eupercaria
Tetraodon nigroviridis	Tetraodontidae	Tetraodontiformes	Series Eupercaria
Channa argus	Channidae	Anabantiformes	Series Anabantaria
Anabas testudineus	Anabantidae	Anabantiformes	Series Anabantaria
Helostoma temminckii	Helostomatidae	Anabantiformes	Series Anabantaria
Lates calcarifer	Centropomidae	*incertae sedis*	Series Carangaria
Caranx melampygus	Carangidae	Carangiformes	Series Carangaria
Selene dorsalis	Carangidae	Carangiformes	Series Carangaria
Cynoglossus semilaevis	Cynoglossidae	Pleuronectiformes	Series Carangaria
Pseudopleuronectes yokohamae	Pleuronectidae	Pleuronectiformes	Series Carangaria
Scophthalmus maximus	Scophthalmidae	Pleuronectiformes	Series Carangaria
Chromis chromis	Pomacentridae	*incertae sedis*	Series Ovalentaria
Parablennius parvicornis	Blenniidae	Blenniiformes	Series Ovalentaria
Pseudochromis fuscus	Pseudochromidae	incertae sedis	Series Ovalentaria
Oryzias latipes	Adrianichthyidae	Beloniformes	Series Ovalentaria
Kryptolebias marmoratus	Rivulidae	Cyprinodontiformes	Series Ovalentaria
Nothobranchius furzeri	Nothobranchiidae	Cyprinodontiformes	Series Ovalentaria
Cyprinodon variegatus	Cyprinodontidae	Cyprinodontiformes	Series Ovalentaria
Fundulus heteroclitus	Fundulidae	Cyprinodontiformes	Series Ovalentaria
Poecilia formosa	Poeciliidae	Cyprinodontiformes	Series Ovalentaria
Xiphophorus maculatus	Poeciliidae	Cyprinodontiformes	Series Ovalentaria
Etroplus canarensis	Cichlidae (Etroplinae)	Cichliformes	Series Ovalentaria
Paratilapia polleni "Andapa"	Cichlidae (Ptychochrominae)	Cichliformes	Series Ovalentaria
Ptychochromis oligacanthus	Cichlidae (Ptychochrominae)	Cichliformes	Series Ovalentaria
Apistogramma diplotaenia	Cichlidae (Cichlinae)	Cichliformes	Series Ovalentaria
Australoheros scitulus	Cichlidae (Cichlinae)	Cichliformes	Series Ovalentaria
Amphilophus citrinellus	Cichlidae (Cichlinae)	Cichliformes	Series Ovalentaria
Amphilophus zaliosus	Cichlidae (Cichlinae)	Cichliformes	Series Ovalentaria
Bujurquina vittata	Cichlidae (Cichlinae)	Cichliformes	Series Ovalentaria
Andinoacara biseriatus	Cichlidae (Cichlinae)	Cichliformes	Series Ovalentaria
Andinoacara coeruleopunctatus	Cichlidae (Cichlinae)	Cichliformes	Series Ovalentaria
Heterochromis multidens	Cichlidae (Pseudocrenilabrinae)	Cichliformes	Series Ovalentaria
Tylochromis polylepis	Cichlidae (Pseudocrenilabrinae)	Cichliformes	Series Ovalentaria
Benitochromis conjunctus	Cichlidae (Pseudocrenilabrinae)	Cichliformes	Series Ovalentaria
Pelvicachromis taeniatus	Cichlidae (Pseudocrenilabrinae)	Cichliformes	Series Ovalentaria
Hemichromis elongatus	Cichlidae (Pseudocrenilabrinae)	Cichliformes	Series Ovalentaria
Etia nguti	Cichlidae (Pseudocrenilabrinae)	Cichliformes	Series Ovalentaria
Oreochromis niloticus	Cichlidae (Pseudocrenilabrinae)	Cichliformes	Series Ovalentaria
Metriaclima zebra	Cichlidae (Pseudocrenilabrinae)	Cichliformes	Series Ovalentaria

Supplementary Table 7: Assembly files used in phylogenetic analyses.
Assembly files are either hosted at the Ensembl (Ensembl.org), NCBI (ncbi.nlm.nih.gov), or EBI (ebi.ac.uk) databases or deposited on datadryad.org, figshare.com, parrot.genomics.cn, surfdrive.surf.nl, cichlid.gurdon.cam.ac.uk, efishgenomics.integrativebiology.msu.edu, or creskolab.uoregon.edu. Details about new assemblies are given in Supplementary Table 2.

Species	Assembly file(s)
Lepisosteus oculatus	Lepisosteus_oculatus.LepOcu1.dna.toplevel.fa.gz
Scleropages formosus	Scleropages_formosus.ASM162426v1.dna.toplevel.fa.gz
Anguilla anguilla	Anguilla_anguilla_assembly_racon_and_double_pilon_corrected.fasta
Clupea harengus	GCA_900323705.1_C.harengus_genomic.fna.gz
Danio rerio	Danio_rerio.GRCz11.dna.toplevel.fa.gz
Astyanax mexicanus	Astyanax_mexicanus.Astyanax_mexicanus-2.0.dna.toplevel.fa.gz
Electrophorus electricus	SSPACEgenomeLLT_454_10212014.fasta.gz
Esox lucius	Esox_lucius.Eluc_V3.dna.toplevel.fa.gz
Salmo salar	GCF_000233375.1_ICSASG_v2_genomic.fna.gz
Borostomias antarcticus	fish_65.scf.fasta.gz
Osmerus eperlanus	fish_54.scf.fasta.gz
Protosalanx hyalocranius	clearhead_icefish.fa.tar.gz
Guentherus altivela	fish_50.scf.fasta.gz
Parasudis fraserbrunneri	fish_42.scf.fasta.gz
Benthosema glaciale	fish_66.scf.fasta.gz
Polymixia japonica	fish_24.scf.fasta.gz
Percopsis transmontana	fish_26.scf.fasta.gz
Typhlichthys subterraneus	fish_27.scf.fasta.gz
Cyttopsis rosea	fish_30.scf.fasta.gz
Zeus faber	fish_28.scf.fasta.gz
Stylephorus chordatus	fish_80.scf.fasta.gz
Gadus morhua	LN845748.fasta, LN845749.fasta, LN845750.fasta, LN845751.fasta, LN845752.fasta, LN845753.fasta, LN845754.fasta, LN845755.fasta, LN845756.fasta, LN845757.fasta, LN845758.fasta, LN845759.fasta, LN845760.fasta, LN845761.fasta, LN845762.fasta, LN845763.fasta, LN845764.fasta, LN845765.fasta, LN845766.fasta, LN845767.fasta, LN845768.fasta, LN845769.fasta, LN845770.fasta
Lampris guttatus	fish_48.scf.fasta.gz
Regalecus glesne	fish_47.scf.fasta.gz
Gephyroberyx darwini	CABFOQ01.fasta.gz
Monocentris japonica	fish_74.scf.fasta.gz
Acanthochaenus luetkenii	fish_79.scf.fasta.gz
Beryx splendens	fish_69.scf.fasta.gz
Holocentrus rufus	fish_35.scf.fasta.gz
Myripristis jacobus	fish_34.scf.fasta.gz
Brotula barbata	fish_32.scf.fasta.gz
Carapus acus	fish_33.scf.fasta.gz
Chatrabus melanurus	fish_40.scf.fasta.gz
Lesueurigobius cf. *sanzoi*	fish_96.scf.fasta.gz
Periophthalmodon schlosseri	GCA_000787095.1_PS.fa_genomic.fna.gz
Hippocampus comes	GCF_001891065.1_H_comes_QL1_v1_genomic.fna.gz
Syngnathus scovelli	ssc_2016_12_20_chromlevel.fa.gz
Pampus argenteus	GCA_000697985.1_PamArg1.0_genomic.fna.gz
Thunnus albacares	fish_83.scf.fasta.gz

Supplementary Table 7 (continued): Assembly files used in phylogenetic analyses.

Species	Assembly file(s)
Epinephelus aeneus	Epae.scf.fasta.gz
Perca fluviatilis	fish_55.scf.fasta.gz
Chaenocephalus aceratus	fish_61.scf.fasta.gz
Anoplopoma fimbria	GCA_000499045.1_AnoFim1.0_genomic.fna.gz
Gasterosteus aculeatus	Gasterosteus_aculeatus.BROADS1.dna.toplevel.fa.gz
Myoxocephalus scorpius	fish_91.scf.fasta.gz
Labrus bergylta	Labrus_bergylta.BallGen_V1.dna.toplevel.fa.gz
Larimichthys crocea	GCA_900246015.1_Larimichthys_crocea_chromosome_1.0_genomic.fna.gz
Dicentrarchus labrax	GCA_000689215.1_seabass_V1.0_genomic.fna.gz
Morone saxatilis	GCA_001663605.1_SBDraft1_genomic.fna.gz
Spondyliosoma cantharus	fish_81.scf.fasta.gz
Antennarius striatus	fish_52.scf.fasta.gz
Mola mola	Mola_mola.ASM169857v1.dna.toplevel.fa.gz
Takifugu rubripes	Takifugu_rubripes.FUGU5.dna.toplevel.fa.gz
Tetraodon nigroviridis	Tetraodon_nigroviridis.TETRAODON8.dna.toplevel.fa.gz
Channa argus	Argus_liugm_genome.fa.gz
Anabas testudineus	fish_85.scf.fasta.gz
Helostoma temminckii	fish_84.scf.fasta.gz
Lates calcarifer	GCA_001640805.1_ASM164080v1_genomic.fna.gz
Caranx melampygus	Came.scf.fasta.gz
Selene dorsalis	fish_86.scf.fasta.gz
Cynoglossus semilaevis	GCF_000523025.1_Cse_v1.0_genomic.fna.gz
Pseudopleuronectes yokohamae	GCA_000787555.1_Pyoko_1.0_genomic.fna.gz
Scophthalmus maximus	Scophthalmus_maximus.ASM318616v1.dna.toplevel.fa.gz
Chromis chromis	fish_87.scf.fasta.gz
Parablennius parvicornis	fish_88.scf.fasta.gz
Pseudochromis fuscus	fish_90.scf.fasta.gz
Oryzias latipes	Oryzias_latipes_hsok.ASM223469v1.dna.toplevel.fa.gz
Kryptolebias marmoratus	Kryptolebias_marmoratus.ASM164957v1.dna.toplevel.fa.gz
Nothobranchius furzeri	LN609377.fasta, LN609378.fasta, LN609379.fasta, LN609380.fasta, LN609381.fasta<br>LN609382.fasta, LN609383.fasta, LN609384.fasta, LN609385.fasta, LN609386.fasta<br>LN609387.fasta, LN609388.fasta, LN609389.fasta, LN609390.fasta, LN609391.fasta<br>LN609392.fasta, LN609393.fasta, LN609394.fasta, LN609395.fasta
Cyprinodon variegatus	Cyprinodon_variegatus.C_variegatus-1.0.dna.toplevel.fa.gz
Fundulus heteroclitus	Fundulus_heteroclitus.Fundulus_heteroclitus-3.0.2.dna.toplevel.fa.gz
Poecilia formosa	Poecilia_formosa.PoeFor_5.1.2.dna.toplevel.fa.gz
Xiphophorus maculatus	Xiphophorus_maculatus.X_maculatus-5.0-male.dna.toplevel.fa.gz
Etroplus canarensis	New
Paratilapia polleni "Andapa"	New
Ptychochromis oligacanthus	New
Apistogramma diplotaenia	New
Australoheros scitulus	New
Amphilophus citrinellus	Amphilophus_citrinellus.Midas_v5.dna.toplevel.fa.gz
Amphilophus zaliosus	New
Bujurquina vittata	New
Andinoacara biseriatus	New
Andinoacara coeruleopunctatus	Andinoacara_coeruleopunctatus_final_min1000bp_scaffolds.fa.gz

Supplementary Table 7 (continued): Assembly files used in phylogenetic analyses.

Species	Assembly file(s)
Heterochromis multidens	New
Tylochromis polylepis	New
Benitochromis conjunctus	New
Pelvicachromis taeniatus	New
Hemichromis elongatus	New
Etia nguti	New
Oreochromis niloticus	MKQE02.1.fsa_nt.gz, MKQE02.2.fsa_nt.gz
Metriaclima zebra	AGTA05.1.fsa_nt.gz, AGTA05.2.fsa_nt.gz

Supplementary Table 8: Markers selected for phylogenetic analyses.
“Gene ID” indicates the Ensembl ID of the zebrafish gene; “mean BPP” specifies the mean node support measured as Bayesian posterior probability in single-gene analyses with BEAST 2. “Rate” and “Coefficient” specify the mean and the coefficient of variation of the substitution-rate estimate in these single-gene analyses. The last column indicates whether the marker is included in the permissive (p) and strict (s) marker sets. var., variable.

Gene ID	# exons	Length (bp)	# var. sites	Mean BPP	Rate	Coefficient	Set
ENSDARG00000000853	4	702	356	0.673	0.00148	0.652	p
ENSDARG00000001129	4	702	338	0.655	0.00130	0.693	p
ENSDARG00000002332	3	513	237	0.570	0.00132	0.474	p+s
ENSDARG00000002402	6	1,287	572	0.735	0.00112	0.594	p+s
ENSDARG00000002600	5	930	470	0.756	0.00133	0.561	p+s
ENSDARG00000002952	4	777	356	0.716	0.00127	0.516	p+s
ENSDARG00000003449	4	645	301	0.547	0.00086	0.624	p
ENSDARG00000003495	7	1,332	652	0.797	0.00136	0.598	p+s
ENSDARG00000003963	5	1,110	494	0.777	0.00080	0.598	p+s
ENSDARG00000004173	6	1,143	522	0.769	0.00116	0.572	p+s
ENSDARG00000004302	4	807	360	0.696	0.00094	0.621	p
ENSDARG00000004581	3	483	243	0.531	0.00124	0.515	p+s
ENSDARG00000005218	4	756	443	0.819	0.00144	0.570	p
ENSDARG00000005236	4	720	312	0.669	0.00110	0.562	p+s
ENSDARG00000007708	4	708	321	0.691	0.00104	0.571	p+s
ENSDARG00000007901	6	1,095	566	0.746	0.00122	0.518	p+s
ENSDARG00000008224	3	564	268	0.553	0.00126	0.532	p+s
ENSDARG00000008575	9	1,737	937	0.841	0.00118	0.570	p
ENSDARG00000008637	4	765	454	0.720	0.00138	0.492	p+s
ENSDARG00000009830	6	1,287	647	0.879	0.00110	0.599	p+s
ENSDARG00000009953	6	1,179	608	0.814	0.00113	0.662	p
ENSDARG00000009982	4	714	342	0.755	0.00141	0.530	p
ENSDARG00000010238	3	531	259	0.620	0.00117	0.635	p
ENSDARG00000010862	3	507	307	0.659	0.00121	0.469	p+s
ENSDARG00000012138	4	699	346	0.591	0.00140	0.468	p+s
ENSDARG00000012378	3	522	241	0.614	0.00108	0.593	p+s
ENSDARG00000013079	5	942	485	0.757	0.00142	0.602	p
ENSDARG00000013150	6	1,038	506	0.768	0.00127	0.625	p
ENSDARG00000014004	3	537	269	0.611	0.00124	0.543	p+s
ENSDARG00000014634	3	483	227	0.528	0.00104	0.363	p+s
ENSDARG00000016415	6	1,131	624	0.832	0.00143	0.569	p
ENSDARG00000016753	6	1,176	550	0.812	0.00119	0.555	p+s
ENSDARG00000016775	6	1,095	553	0.836	0.00146	0.552	p
ENSDARG00000016782	5	948	432	0.739	0.00110	0.624	p
ENSDARG00000017006	4	807	396	0.678	0.00115	0.586	p+s
ENSDARG00000017986	9	1,599	822	0.807	0.00122	0.590	p+s
ENSDARG00000018192	13	2,550	1,169	0.907	0.00117	0.580	p+s
ENSDARG00000018325	6	1,254	681	0.749	0.00134	0.564	p+s
ENSDARG00000018904	3	507	227	0.445	0.00110	0.633	p
ENSDARG00000018973	3	588	258	0.746	0.00124	0.545	p+s
ENSDARG00000019000	9	1,560	644	0.800	0.00096	0.580	p+s

Supplementary Table 8 (continued): Markers selected for phylogenetic analyses.

Gene ID	# exons	Length (bp)	# var. sites	Mean BPP	Rate	Coefficient	Set
ENSDARG00000019451	3	873	397	0.711	0.00124	0.520	p+s
ENSDARG00000020242	3	624	257	0.688	0.00100	0.558	p+s
ENSDARG00000020693	3	618	341	0.550	0.00137	0.551	p+s
ENSDARG00000021945	6	1,182	485	0.843	0.00104	0.617	p
ENSDARG00000022974	4	897	409	0.732	0.00103	0.610	p
ENSDARG00000024317	3	498	277	0.495	0.00109	0.407	p
ENSDARG00000025011	4	771	375	0.662	0.00120	0.571	p+s
ENSDARG00000025094	3	513	257	0.619	0.00094	0.506	p+s
ENSDARG00000025212	3	576	222	0.610	0.00083	0.525	p+s
ENSDARG00000026180	19	3,696	1,366	0.925	0.00090	0.634	p
ENSDARG00000027016	3	570	327	0.603	0.00115	0.660	p
ENSDARG00000027353	6	999	550	0.822	0.00156	0.589	p
ENSDARG00000028748	3	519	223	0.720	0.00119	0.551	p
ENSDARG00000028971	4	858	416	0.746	0.00111	0.504	p+s
ENSDARG00000029157	6	1,146	533	0.765	0.00111	0.559	p+s
ENSDARG00000029472	3	549	253	0.600	0.00108	0.566	p+s
ENSDARG00000029931	3	486	213	0.675	0.00078	0.581	p+s
ENSDARG00000030022	3	561	321	0.712	0.00145	0.658	p
ENSDARG00000030665	3	534	259	0.627	0.00156	0.579	p
ENSDARG00000034396	4	774	419	0.683	0.00137	0.554	p+s
ENSDARG00000034823	3	585	232	0.599	0.00112	0.392	p+s
ENSDARG00000035535	4	630	299	0.585	0.00123	0.557	p+s
ENSDARG00000035751	5	894	463	0.737	0.00121	0.659	p
ENSDARG00000035978	6	1,218	630	0.849	0.00122	0.613	p
ENSDARG00000039931	3	630	360	0.600	0.00131	0.557	p+s
ENSDARG00000040571	5	993	555	0.782	0.00110	0.570	p+s
ENSDARG00000041133	3	693	357	0.661	0.00122	0.685	p
ENSDARG00000041217	3	504	292	0.708	0.00128	0.631	p
ENSDARG00000042087	3	525	256	0.641	0.00125	0.618	p
ENSDARG00000042147	3	486	192	0.568	0.00109	0.493	p+s
ENSDARG00000043019	6	1,107	508	0.780	0.00093	0.660	p
ENSDARG00000043359	3	480	209	0.517	0.00096	0.525	p+s
ENSDARG00000044820	3	843	377	0.607	0.00099	0.566	p+s
ENSDARG00000045275	3	501	218	0.550	0.00096	0.572	p+s
ENSDARG00000045399	3	504	222	0.704	0.00092	0.454	p+s
ENSDARG00000045560	3	528	266	0.651	0.00126	0.531	p+s
ENSDARG00000051921	3	582	284	0.625	0.00144	0.520	p
ENSDARG00000052377	3	531	255	0.606	0.00091	0.515	p+s
ENSDARG00000054224	3	540	289	0.613	0.00155	0.641	p
ENSDARG00000055554	3	1,002	528	0.801	0.00122	0.635	p
ENSDARG00000056557	6	1,119	503	0.794	0.00088	0.621	p
ENSDARG00000056587	3	1,113	679	0.813	0.00147	0.570	p
ENSDARG00000056648	4	771	385	0.653	0.00130	0.652	p
ENSDARG00000057738	3	537	272	0.582	0.00108	0.507	p+s
ENSDARG00000058419	11	2,142	1,061	0.865	0.00106	0.584	p+s
ENSDARG00000059116	10	1,926	876	0.808	0.00102	0.604	p
ENSDARG00000059323	5	1,128	639	0.844	0.00119	0.486	p+s

Supplementary Table 8 (continued): Markers selected for phylogenetic analyses.

Gene ID	# exons	Length (bp)	# var. sites	Mean BPP	Rate	Coefficient	Set
ENSDARG00000059553	5	975	535	0.727	0.00116	0.545	p
ENSDARG00000059701	3	504	267	0.642	0.00136	0.472	p+s
ENSDARG00000059911	3	525	306	0.654	0.00137	0.557	p+s
ENSDARG00000060323	3	597	279	0.607	0.00085	0.631	p
ENSDARG00000060785	3	789	469	0.680	0.00157	0.699	p
ENSDARG00000061039	3	585	302	0.606	0.00152	0.621	p
ENSDARG00000061203	5	1,017	461	0.578	0.00103	0.569	p+s
ENSDARG00000061590	4	693	358	0.619	0.00141	0.539	p
ENSDARG00000061723	3	552	287	0.679	0.00123	0.607	p
ENSDARG00000061960	3	543	232	0.644	0.00086	0.616	p
ENSDARG00000062198	3	591	342	0.532	0.00155	0.596	p
ENSDARG00000062472	6	1,131	549	0.789	0.00139	0.620	p
ENSDARG00000062795	3	591	324	0.661	0.00116	0.387	p+s
ENSDARG00000062846	3	612	333	0.675	0.00156	0.663	p
ENSDARG00000062973	3	537	256	0.516	0.00100	0.633	p
ENSDARG00000067913	3	513	243	0.695	0.00107	0.496	p+s
ENSDARG00000068187	4	642	325	0.659	0.00113	0.594	p+s
ENSDARG00000068910	5	930	484	0.717	0.00107	0.575	p+s
ENSDARG00000069289	3	555	253	0.668	0.00110	0.606	p
ENSDARG00000069420	3	657	291	0.494	0.00126	0.526	p+s
ENSDARG00000074524	3	555	299	0.629	0.00131	0.649	p
ENSDARG00000074686	3	612	283	0.637	0.00103	0.588	p+s
ENSDARG00000074759	3	570	217	0.624	0.00102	0.664	p
ENSDARG00000075612	3	552	284	0.599	0.00108	0.488	p+s
ENSDARG00000075884	7	1,593	882	0.784	0.00142	0.521	p
ENSDARG00000076815	7	1,434	627	0.851	0.00095	0.599	p+s
ENSDARG00000077162	3	4,638	2,316	0.796	0.00153	0.662	p
ENSDARG00000077469	3	519	298	0.719	0.00130	0.538	p+s
ENSDARG00000077536	14	2,499	1,111	0.819	0.00112	0.645	p
ENSDARG00000077570	5	1,032	522	0.675	0.00120	0.592	p+s
ENSDARG00000078127	5	849	395	0.661	0.00106	0.542	p+s
ENSDARG00000078853	4	624	320	0.690	0.00103	0.553	p+s
ENSDARG00000079163	3	456	231	0.682	0.00116	0.569	p
ENSDARG00000079308	3	678	374	0.653	0.00138	0.678	p
ENSDARG00000079848	4	702	350	0.654	0.00129	0.544	p+s
ENSDARG00000079949	6	1,158	502	0.659	0.00087	0.566	p+s
ENSDARG00000086790	3	630	269	0.671	0.00155	0.541	p
ENSDARG00000090183	4	1,191	587	0.777	0.00109	0.638	p
ENSDARG00000091367	3	537	226	0.637	0.00098	0.605	p
ENSDARG00000093413	3	519	215	0.484	0.00118	0.646	p
ENSDARG00000094380	5	882	505	0.812	0.00133	0.668	p
ENSDARG00000095890	5	1,029	531	0.871	0.00117	0.623	p
ENSDARG00000098247	3	561	248	0.521	0.00122	0.437	p+s
ENSDARG00000098916	4	684	329	0.718	0.00125	0.534	p+s
ENSDARG00000099034	4	738	395	0.516	0.00150	0.672	p
ENSDARG00000099139	3	546	277	0.615	0.00091	0.427	p+s
ENSDARG00000099458	3	576	263	0.551	0.00144	0.429	p

Supplementary Table 8 (continued): Markers selected for phylogenetic analyses.

Gene ID	# exons	Length (bp)	# var. sites	Mean BPP	Rate	Coefficient	Set
ENSDARG00000099798	3	561	262	0.595	0.00099	0.649	p
ENSDARG00000100013	5	858	351	0.712	0.00096	0.622	p
ENSDARG00000100419	8	1,401	726	0.902	0.00112	0.581	p+s
ENSDARG00000100524	6	1,119	574	0.832	0.00122	0.561	p+s
ENSDARG00000101062	3	549	314	0.668	0.00122	0.487	p+s
ENSDARG00000101507	4	729	376	0.819	0.00132	0.611	p
ENSDARG00000101676	4	1,029	483	0.690	0.00132	0.648	p
ENSDARG00000102184	5	888	346	0.754	0.00111	0.641	p
ENSDARG00000102307	4	726	329	0.660	0.00115	0.564	p+s
ENSDARG00000102587	3	519	246	0.603	0.00107	0.501	p+s
ENSDARG00000103799	3	510	274	0.649	0.00093	0.449	p+s
ENSDARG00000104953	4	660	255	0.551	0.00084	0.683	p
Total (permissive set):							
147 genes	646	127,638	62,190	0.688	0.00118	0.573	
Total (strict set):							
77 genes	332	62,776	30,463	0.678	0.00114	0.535	

Supplementary Table 9: Bayesian age estimates for the divergence of American and African cichlid fishes.
Studies reporting Bayesian molecular divergence-time estimates of African and Neotropical cichlid fishes. Taxon numbers in parentheses indicate sampled species of Pseudocrenilabrinae and Cichlinae. Calibration numbers in parentheses specify how many divergences were constrained within Cichlidae.

Study	Ref.	# taxa	# sites	% complete	# cal.	Age estimate (Ma)
Genner et al. (2007)	[196]	34 (18, 6)	905	96.0	4 (0)	98.4 (121.9-75.4)
Azuma et al. (2008)	[197]	54 (4, 2)	10 034	99.9	18 (0)	89.0 (108.0-72.0)
Santini et al. (2009)	[113]	227 (6, 1)	1 445	93.1	45 (2)	49.0 (66.0-37.0)
Matschiner et al. (2011)	[198]	67 (4, 2)	4 599	93.5	6 (0)	59.7 (80.7-40.2)
Matschiner (unpubl.)		9 (3, 1)	28 239	92.6	3 (1)	55.2 (67.4-49.7)
McMahan et al. (2013)	[199]	133 (19, 57)	2 069	88.2	4 (3)	70.8 (83.7-60.2)
Near et al. (2013)	[200]	579 (2, 3)	8 577	84.5	37 (0)	25.9 (29.6-22.0)
Friedman et al. (2013)	[5]	158 (54, 29)	7 887	93.7	10 (0)	46.4 (54.9-40.9)
Betancur-R. et al. (2013)	[201]	202 (3, 4)	17 812	51.5	59 (2)	62.1 (70.2-54.7)
Matschiner et al. (2017)	[18]	366 (64, 79)	27 650	40.7	147 (23)	81.6 (89.4-74.0)
Hughes et al. (2018)	[7]	302 (9, 2)	10 203	96.4	31 (1)	52.2 (65.9-45.7)
Musilova et al. (2019)	[9]	101 (5, 1)	71 902	93.6	28 (2)	55.1 (66.9-45.3)
Roth et al. (2010)	[202]	71 (1, 1)	108 372	93.2	34 (2)	50.3 (57.6-45.1)
This study		91 (8, 7)	127 638	93.5	51 (8)	62.1 (70.1-54.6)
This study (excl. cichlid fossils)					43 (0)	38.7 (50.8-27.1)

Supplementary References

1. Tyler, J. C. & Sorbini, L. New superfamily and three new families of tetraodontiform fishes from the Upper Cretaceous: the earliest and most morphologically primitive plectognaths. *Smithson. Contr. Paleobiol.* **82,** 1–59 (1996).

2. Santini, F. & Tyler, J. C. A phylogeny of the families of fossil and extant tetraodontiform fishes (Acanthomorpha, Tetraodontiformes), Upper Cretaceous to Recent. *Zool. J. Linn. Soc.* **139,** 565–617 (2003).

3. Benton, M. J. *et al.* Constraints on the timescale of animal evolutionary history. *Palaeontol. Electron.* **18.1.1FC,** 1–106 (2015).

4. Santini, F., Sorenson, L., Marcroft, T., Dornburg, A. & Alfaro, M. E. A multilocus molecular phylogeny of boxfishes (Aracanidae, Ostraciidae; Tetraodontiformes). *Mol. Phylogenet. Evol.* **66,** 153–160 (2013).

5. Friedman, M. *et al.* Molecular and fossil evidence place the origin of cichlid fishes long after Gondwanan rifting. *Proc. R. Soc. B* **280,** 20131733 (2013).

6. Dornburg, A., Townsend, J. P., Friedman, M. & Near, T. J. Phylogenetic informativeness reconciles ray-finned fish molecular divergence times. *BMC Evol. Biol.* **14,** 169 (2014).

7. Hughes, L. C. *et al.* Comprehensive phylogeny of ray-finned fishes (Actinopterygii) based on transcriptomic and genomic data. *Proc. Natl. Acad. Sci. USA* **5,** 201719358 (2018).

8. Alfaro, M. E. *et al.* Explosive diversification of marine fishes at the Cretaceous–Palaeogene boundary. *Nat. Ecol. Evol.* **2,** 688–696 (2018).

9. Musilova, Z. *et al.* Vision using multiple distinct rod opsins in deep-sea fishes. *Science* **364,** 588–592 (2019).

10. Arcila, D. & Tyler, J. C. Mass extinction in tetraodontiform fishes linked to the Palaeocene–Eocene thermal maximum. *Proc. R. Soc. B* **284,** 20171771 (2017).

11. Pyron, R. A. Divergence time estimation using fossils as terminal taxa and the origins of Lissamphibia. *Syst. Biol.* **60,** 466–481 (2011).

12. Ronquist, F. *et al.* A total-evidence approach to dating with fossils, applied to the early radiation of the Hymenoptera. *Syst. Biol.* **61,** 973–999 (2012).

13. Gavryushkina, A. *et al.* Bayesian total-evidence dating reveals the recent crown radiation of penguins. *Syst. Biol.* **66,** 57–73 (2017).

14. Guindon, S. Bayesian estimation of divergence times from large sequence alignments. *Mol. Biol. Evol.* **27,** 1768–1781 (2010).

15. Guindon, S. Accounting for calibration uncertainty: Bayesian molecular dating as a "doubly intractable" problem. *Syst. Biol.* **67,** 651–661 (2018).

16. Benton, M. & Donoghue, P. Paleontological evidence to date the tree of life. *Mol. Biol. Evol.* **24,** 26–53 (2007).

17. Arcila, D., Pyron, R. A., Tyler, J. C., Orti, G. & Betancur-R, R. An evaluation of fossil tip-dating versus node-age calibrations in tetraodontiform fishes (Teleostei: Percomorphaceae). *Mol. Phylogenet. Evol.* **82,** 131–145 (2015).

18. Matschiner, M. *et al.* Bayesian phylogenetic estimation of clade ages supports trans-Atlantic dispersal of cichlid fishes. *Syst. Biol.* **66,** 3–22 (2017).

19. Brown, J. W. & Smith, S. A. The past sure is tense: on interpreting phylogenetic divergence time estimates. *Syst. Biol.* **67,** 340–353 (2018).

20. Betancur-R, R. *et al.* Phylogenetic classification of bony fishes. *BMC Evol. Biol.* **17,** 162 (2017).

21. Cohen, K. M., Finney, S. C., Gibbard, P. L. & Fan, J. X. The ICS international chronostratigraphic chart. *Episodes* **36,** 199–204 (2013).

22. Cavin, L. Diversity of Mesozoic semionotiform fishes and the origin of gars (Lepisosteidae). *Naturwissenschaften* **97,** 1035–1040 (2010).

23. López-Arbarello, A. Phylogenetic interrelationships of ginglymodian fishes (Actinopterygii: Neopterygii). *PLoS ONE* **7,** e39370 (2012).

24. Giles, S., Xu, G.-H., Near, T. J. & Friedman, M. Early members of 'living fossil' lineage imply later origin of modern ray-finned fishes. *Nature* **549,** 265–268 (2017).

25. Kirkby, J. W. On the remains of fish and plants from the Upper Limestone of the Permian series of Durham. *Ann. Mag. Nat. Hist. 3* **9,** 267–269 (1862).

26. Broughton, R. E., Betancur-R, R., Li, C., Arratia, G. & Orti, G. Multi-locus phylogenetic analysis reveals the pattern and tempo of bony fish evolution. *PLoS Currents: Tree of Life,* doi:10.1371/currents.tol.2ca8041495ffafd0c92756e75247483e (2013).

27. Lee, M. R. *Formation and diagenesis of slope limestones within the Upper Permian (Zechstein) Raisby Formation, north-east England* in *Proceedings of the Yorkshire Geological Society* (1993), 215–227.

28. Benton, M. J., Donoghue, P. C. J. & Asher, R. J. in *The Timetree of Life* (eds Hedges, S. B. & Kumar, S.) 35–86 (Oxford University Press, Oxford, United Kingdom, 2009).

29. Arratia, G. Morphology, taxonomy, and phylogeny of Triassic pholidophorid fishes (Actinopterygii, Teleostei). *J. Vertebr. Paleontol.* **33,** 1–138 (2013).

30. Arratia, G. New Triassic teleosts (Actinopterygii, Teleosteomorpha) from northern Italy and their phylogenetic relationships among the most basal teleosts. *J. Vertebr. Paleontol.* **3,** e1312690 (2017).

31. Tintori, A. The actinopterygian fish *Prohalecites* from the Triassic of Northern Italy. *Paleontology* **33,** 155–174 (1990).

32. Arratia, G. & Tintori, A. in *Mesozoic Fishes 2 – Systematics and Fossil Record* (eds Arratia, G. & Schultze, H.-P.) 121–142 (Verlag Dr. Friedrich Pfeil, München, Germany, 1999).

33. Wilson, M. V. H. & Murray, A. M. Osteoglossomorpha: phylogeny, biogeography, and fossil record and the significance of key African and Chinese fossil taxa. *Geological Society, London, Special Publications* **295,** 185–219 (2008).

34. Chen, P. *et al.* Jianshangou Bed of the Yixian Formation in West Liaoning, China. *Sci China Ser D* **48,** 298 (2005).

35. Chen, D. *et al.* Timing of the late Jehol Biota: New geochronometric constraints from the Jixi Basin, NE China. *Palaeogeogr. Palaeoclimatol. Palaeoecol.* **492,** 41–49 (2018).

36. Arratia, G. Remarkable teleostean fishes from the Late Jurassic of southern Germany and their phylogenetic relationships. *Mitteilungen aus dem Museum für Naturkunde in Berlin, Geowissenschaftliche Reihe* **3,** 137–179 (2000).

37. Schweigert, G. Ammonite biostratigraphy as a tool for dating Upper Jurassic lithographic limestones from South Germany – first results and open questions. *N. Jb. Geol. Pal. A* **245,** 117–125 (2007).

38. Dietl, G. & Schweigert, G. The Nusplingen Lithographic Limestone - A "fossil Lagerstaette" of Late Kimmeridgian age from the Swabian Alb (Germany). *Rivista Italiana di Paleontologia e Stratigrafia* **110,** 303–309 (2004).

39. Fürsich, F. T., Mäuser, M., Schneider, S. & Werner, W. The Wattendorf Plattenkalk (Upper Kimmeridgian) – a new conservation lagerstätte from the northern Franconian Alb, southern Germany. *N. Jb. Geol. Pal. A* **245,** 45–58 (2007).

40. Ogg, J. G., Hinnov, L. A. & Huang, C. in *The Geologic Time Scale 2012* (ed Gradstein, F. M.) 731–791 (Elsevier, Amsterdam, Netherlands, 2012).

41. Arratia, G. Basal teleosts and teleostean phylogeny. *Palaeo Ichthyologica* **7,** 5–168 (1997).

42. Uyeno, T. & Yabumoto, Y. Early Cretaceous freshwater fishes from Northern Kyushu, Japan: II. Restoration of two species of the clupeoid fish genus *Diplomystus*. *Bulletin of the Kitakyushu Museum of Natural History* **2,** 25–31 (1980).

43. Matsumoto, T. *et al.* Correlation of marine and non-marine formation in the Cretaceous of Japan. *Fossils* **31,** 1–26 (1982).

44. Kimura, T., Ohana, T. & Naito, G. *Cupressionocladus* sp., newly found from the Lower Cretaceous Wakino Formation, West Japan. *Bulletin of the Kitakyushu Museum of Natural History* **11,** 79–86 (1992).

45. Murray, A. M. & Wilson, M. V. H. in *Mesozoic Fishes 5 - Global Diversity and Evolution* (eds Arratia, G., Schultze, H.-P. & Wilson, M. V. H.) 267–290 (Verlag Dr. Friedrich Pfeil, München, Germany, 2013).

46. De Figueiredo, F. J. & Ribeiro, D. R. M. Reassessment and relationships of †*Scutatuspinosus itapagipensis* (Teleostei, Clupeomorpha, †Ellimmichthyiformes) from the Neocomian of Recôncavo Basin, Northeastern Brazil. *An. Acad. Bras. Ciênc.* **89,** 799–823 (2017).

47. Marramà, G., Bannikov, A. F., Kriwet, J. & Carnevale, G. An Eocene paraclupeid fish (Teleostei, Ellimmichthyiformes) from Bolca, Italy: the youngest marine record of double-armoured herrings. *Pap. Palaeontol.* **34,** 234 (2018).

48. Poyato-Ariza, F. J., López-Horgue, M. A. & Garcıéa-Garmilla, F. A new early Cretaceous clupeomorph fish from the Arratia Valley, Basque Country, Spain. *Cretaceous Res.* **21,** 571–585 (2000).

49. Liu, J., Chang, M.-M., Wilson, M. V. H. & Murray, A. M. A new family of Cypriniformes (Teleostei, Ostariophysi) based on a redescription of †*Jianghanichthys hubeiensis* (Lei, 1977) from the Eocene Yangxi Formation of China. *J. Vertebr. Paleontol.* **35,** e1004073 (2015).

50. Hirt, M. V. *et al.* Effects of gene choice, base composition and rate heterogeneity on inference and estimates of divergence times in cypriniform fishes. *Biol. J. Linn. Soc.* **121,** 319–339 (2017).

51. Wilson, M. V. H. Oldest known *Esox* (Pisces: Esocidae), part of a new Paleocene teleost fauna from western Canada. *Can. J. Earth Sci.* **17,** 307–312 (1980).

52. Vandenberghe, N., Hilgen, F. J. & Speijer, R. P. in *The Geologic Time Scale 2012* (ed Gradstein, F. M.) 855–921 (Elsevier, Amsterdam, Netherlands, 2012).

53. Filleul, A. & Maisey, J. G. Redescription of *Santanichthys diasii* (Otophysi, Characiformes) from the Albian of the Santana Formation and comments on its implications for otophysan relationships. *Am. Mus. Novit.* **3455,** 21 pp. (2004).

54. Otero, O., Valentin, X. & Garcia, G. Cretaceous characiform fishes (Teleostei: Ostariophysi) from Northern Tethys: description of new material from the Maastrichtian of Provence (Southern France) and palaeobiogeographical implications. *Geological Society, London, Special Publications* **295,** 155–164 (2008).

55. Dai, W. *et al.* Phylogenomic perspective on the relationships and evolutionary history of the major otocephalan lineages. *Sci. Rep.* **8,** 205 (2018).

56. Martill, D. M. The age of the Cretaceous Santana Formation fossil Konservat Lagerstätte of north-east Brazil: a historical review and an appraisal of the biochronostratigraphic utility of its palaeobiota. *Cretaceous Res.* **28,** 895–920 (2007).

57. Silveira Antonietto, L., Aparecido do Carmo, D., Claudia Viviers, M. & Rodrigues Adôrno, R. Biostratigraphic and paleozoogeographic review of the upper Aptian-Albian ostracods of Riachuelo Formation, Sergipe-Alagoas Basin, Northeastern Brazil. *Rev. Bras. Paleontolog.* **18,** 355–368 (2015).

58. Brito, P. M., Lindoso, R. M., Carvalho, I. S. & de Paula Machado, G. Discovery of †Obaichthyidae gars (Holostei, Ginglymodi, Lepisosteiformes) in the Aptian Codó Formation of the Parnaıéba Basin: Remarks on paleobiogeographical and temporal range. *Cretaceous Res.* **59,** 10–17 (2016).

59. Wilson, M., Brinkman, D. B. & Neuman, A. G. Cretaceous Esocoidei (Teleostei): early radiation of the pikes in North American fresh waters. *J. Paleontol.* (1992).

60. Payenberg, T. H., Braman, D. R., Davis, D. W. & Miall, A. D. Litho- and chronostratigraphic relationships of the Santonian-Campanian Milk River Formation in southern Alberta and Eagle Formation in Montana utilising stratigraphy, U-Pb geochronology, and palynology. *Can. J. Earth Sci.* **39,** 1553–1577 (2002).

61. Wilson, M. V. H. & Li, G.-Q. Osteology and systematic position of the Eocene salmonid †*Eosalmo driftwoodensis* Wilson from western North America. *Zool. J. Linn. Soc.* **125,** 279–311 (1999).

62. Gallo, V., de Figueiredo, F. J. & Azevedo, S. A. *Santanasalmo elegans* gen. et sp. nov., a basal euteleostean fish from the Lower Cretaceous of the Araripe Basin, northeastern Brazil. *Cretaceous Res.* **30,** 1357–1366 (2009).

63. Moss, P. T., Greenwood, D. R. & Archibald, S. B. Regional and local vegetation community dynamics of the Eocene Okanagan Highlands (British Columbia - Washington State) from palynology. *Can. J. Earth Sci.* **42,** 187–204 (2005).

64. Eberle, J. J. & Rybczynski, N. Early Eocene mammals from the Driftwood Creek Beds, Driftwood Canyon Provincial Park, northern British Columbia. *J. Vertebr. Paleontol.* **34,** 737–746 (2014).

65. Carnevale, G. & Rindone, A. The teleost fish *Paravinciguerria praecursor* Arambourg, 1954 in the Cenomanian of north-eastern Sicily. *Bollettino della Società Paleontologica Italiana* **50,** 1–10 (2011).

66. Khalloufi, B., Ouarhache, D. & Lelièvre, H. New paleontological and geological data about Jbel Tselfat (Late Cretaceous of Morocco). *Hist. Biol.* **22,** 57–70 (2010).

67. Scopelliti, G. *et al.* Cenomanian–Turonian carbonate and organic-carbon isotope records, biostratigraphy and provenance of a key section in NE Sicily, Italy: Palaeoceanographic and palaeogeographic implications. *Palaeogeogr. Palaeoclimatol. Palaeoecol.* **265,** 59–77 (2008).

68. Ogg, J. G. & Hinnov, L. A. in *The Geologic Time Scale 2012* (ed Gradstein, F. M.) 793–853 (Elsevier, Amsterdam, Netherlands, 2012).

69. Wilson, M. V. H. & Williams, R. G. New paleocene genus and species of smelt (Teleostei: Osmeridae) from freshwater deposits of the Paskapoo Formation, Alberta, Canada, and comments on osmerid phylogeny. *J. Vertebr. Paleontol.* **11,** 434–451 (1991).

70. Wilson, M. V. H. & Williams, R. G. in *Origin and Phylogenetic Interrelationships of Teleosts* (eds Nelson, J. S., Schultze, H.-P. & Wilson, M. V. H.) 379–409 (Verlag Dr. Friedrich Pfeil, München, Germany, 2010).

71. Hoffman, G. L. & Stockey, R. A. Geological setting and paleobotany of the Joffre Bridge Roadcut fossil locality (Late Paleocene), Red Deer Valley, Alberta. *Can. J. Earth Sci.* **36,** 2073–2084 (1999).

72. Gaudant, J. Présence d'un Osmeridae: *Enoplophthalmus schlumbergeri* Sauvage, 1880 dans l'Oligocène inférieur des environs de Céreste (Alpes-de-Haute-Provence, France). *Geodiversitas* **35,** 345–357 (2013).

73. Cavin, L. *Les poissons d'eau douce: 250 millions d'années d'histoire évolutive* (ISTE Editions, Ltd., London, United Kingdom, 2018).

74. Kriwet, J. Lancetfish teeth (Neoteleostei, Alepisauroidei) from the Early Cretaceous of Alcaine, NE Spain. *Lethaia* **36,** 323–331 (2003).

75. Davis, M. P. & Fielitz, C. Estimating divergence times of lizardfishes and their allies (Euteleostei: Aulopiformes) and the timing of deep-sea adaptations. *Mol. Phylogenet. Evol.* **57,** 1194–1208 (2010).

76. Soria, A. R., Martıén-Closas, C., Meléndez, A., Meléndez, M. N. & Aurell, M. Estratigrafia del Cretacico inferior continental de la Cordillera Iberica central. *Estudios Geologicos* **51,** 141–152 (1995).

77. Martıén-Closas, C. & Wang, Q. Historical biogeography of the lineage *Atopochara trivolvis* PECK 1941 (Cretaceous Charophyta). *Palaeogeogr. Palaeoclimatol. Palaeoecol.* **260,** 435–451 (2008).

78. Gallo, V. & Coelho, P. M. in *Mesozoic Fishes 4 - Homology and Phylogeny* (eds Arratia, G., Schultze, H.-P. & Wilson, M. V. H.) 351–371 (Verlag Dr. Friedrich Pfeil, München, Germany, 2008).

79. Newbrey, M. G. & Konishi, T. A new lizardfish (Teleostei, Aulopiformes) from the Late Cretaceous Bearpaw Formation of Alberta, Canada, with a revised diagnosis of *Apateodus* (Aulopiformes, Ichthyotringoidei). *J. Vertebr. Paleontol.* **35,** e918042 (2015).

80. Garcia, G. G., Garcia, A. J. V. & Henriques, M. H. P. Palynology of the Morro do Chaves Formation (Lower Cretaceous), Sergipe Alagoas Basin, NE Brazil: Paleoenvironmental implications for the early history of the South Atlantic. *Cretaceous Res.* **90,** 7–20 (2018).

81. Prokofiev, A. M. Fossil myctophoid fishes (Myctophiformes: Myctophoidei) from Russia and adjacent regions. *J. Ichthyol.* **46,** S38–S83 (2006).

82. Dietze, K. Morphology and phylogenetic relationships of certain neoteleostean fishes from the Upper Cretaceous of Sendenhorst, Germany. *Cretaceous Res.* **30,** 559–574 (2009).

83. Dornburg, A., Friedman, M. & Near, T. J. Phylogenetic analysis of molecular and morphological data highlights uncertainty in the relationships of fossil and living species of Elopomorpha (Actinopterygii: Teleostei). *Mol. Phylogenet. Evol.* **89,** 205–218 (2015).

84. Murray, A. M. Mid-Cretaceous acanthomorph fishes with the description of a new species from the Turonian of Lac des Bois, Northwest Territories, Canada. *Vertebrate Anatomy Morphology Palaeontology* **1,** 101–115 (2016).

85. Forey, P. L., Yi, L., Patterson, C. & Davies, C. E. Fossil fishes from the Cenomanian (Upper Cretaceous) of Namoura, Lebanon. *J. Syst. Palaeontol.* **1,** 227–330 (2003).

86. Gonzáles-Rodrıéguez, K. A., Schultze, H.-P. & Arratia, G. in *Mesozoic Fishes 5 - Global Diversity and Evolution* (eds Arratia, G., Schultze, H.-P. & Wilson, M. V. H.) 457–487 (Verlag Dr. Friedrich Pfeil, München, Germany, 2013).

87. González-Rodrıéguez, K. & Fielitz, C. in *Mesozoic Fishes 4 - Homology and Phylogeny* (eds Arratia, G., Schultze, H.-P. & Wilson, M. V. H.) 399–411 (Verlag Dr. Friedrich Pfeil, München, Germany, 2008).

88. Bravo-Cuevas, V. M., Gonzáles-Rodrıéguez, K. A., Esquivel-Macıéas, C. & Fielitz, C. Advances on Stratigraphy and Paleontology of the Muhi Quarry from the Mid-Cretaceous (Albian-Cenomanian) of Hidalgo, Central Mexico. *Boletıén de la Sociedad Geológica Mexicana* **61,** 15–165 (2009).

89. Nelson, J. S., Grande, T. C. & Wilson, M. V. H. *Fishes of the World* 5th ed. (John Wiley & Sons, Inc., Hoboken, New Jersey, 2016).

90. Malmstrøm, M. *et al.* Evolution of the immune system influences speciation rates in teleost fishes. *Nat. Genet.* **48,** 1204–1210 (2016).

91. Rabosky, D. L. *et al.* An inverse latitudinal gradient in speciation rate for marine fishes. *Nature* **559,** 392–395 (2018).

92. Patterson, C. A review of Mesozoic acanthopterygian fishes, with special reference to those of the English Chalk. *Phil. Trans. R. Soc. B* **247,** 213–482 (1964).

93. Jacobs, L. L., Ferguson, K., Polcyn, M. J. & Rennison, C. Cretaceous δ13C stratigraphy and the age of dolichosaurs and early mosasaurs. *Neth J Geosci* **84,** 257–268 (2005).

94. Delbarre, D. J., Davesne, D. & Friedman, M. Anatomy and relationships of *Aipichthys pretiosus* and '*Aipichthys*' *nuchalis* (Acanthomorpha: Lampridomorpha), with a review of Late Cretaceous relatives of oarfishes and their allies. *J. Syst. Palaeontol.* **14,** 545–567 (2016).

95. Alvarado-Ortega, J. & Than-Marchese, B. A Cenomanian aipichthyoid fish (Teleostei, Acanthomorpha) from America, *Zoqueichthys carolinae* gen. and sp. nov. from El Chango quarry (Cintalapa Member, Sierra Madre Formation), Chiapas, Mexico. *Rev. Mex. Cienc. Geol.* **29,** 735–748 (2012).

96. Davesne, D. *et al.* Early fossils illuminate character evolution and interrelationships of Lampridiformes (Teleostei, Acanthomorpha). *Zool. J. Linn. Soc.* **172,** 475–498 (2014).

97. Davesne, D. *et al.* The phylogenetic intrarelationships of spiny-rayed fishes (Acanthomorpha, Teleostei, Actinopterygii): fossil taxa increase the congruence of morphology with molecular data. *Front. Ecol. Evol.* **4,** 735 (2016).

98. Gottfried, M. D., Fordyce, R. E. & Rust, S. *Megalampris keyesi*, a giant moonfish (Teleostei, Lampridiformes) from the Late Oligocene of New Zealand. *J. Vertebr. Paleontol.* **26,** 544–551 (2006).

99. Gottfried, M. D. & Fordyce, R. E. An associated specimen of *Carcharodon angustidens* (Chondrichthyes, Lamnidae) from the Late Oligocene of New Zealand, with comments on *Carcharodon* interrelationships. *J. Vertebr. Paleontol.* **21,** 730–739 (2001).

100. Graham, I. J., Morgans, H. E. G., Waghorn, D. B., Trotter, J. A. & Whitford, D. J. Strontium isotope stratigraphy of the Oligocene-Miocene Otekaike Limestone (Trig Z section) in southern New Zealand: age of the Duntroonian/Waitakian Stage boundary. *New Zeal. J. Geol. Geop.* **43,** 335–347 (2000).

101. Rosen, D. E. & Patterson, C. The structure and relationships of the paracanthopterygian fishes. *Bulletin of the American Museum of Natural History* **141,** 357–474 (1969).

102. Wilson, M. V. H. & Murray, A. M. in *Mesozoic Fishes - Systematics and Paleoecology* (eds Arratia, G. & Viohl, G.) 369–382 (Verlag Dr. Friedrich Pfeil, München, Germany, 1996).

103. Grande, T., Borden, W. C. & Smith, W. L. in *Mesozoic Fishes 5 - Global diversity and Evolution* (eds Arratia, G., Schultze, H.-P. & Wilson, M. V. H.) 385–418 (Verlag Dr. Friedrich Pfeil, München, Germany, 2013).

104. Newbrey, M. G., Murray, A. M., Wilson, M. V. H., Brinkman, D. B. & Neuman, A. G. in *Mesozoic Fishes 5 - Global Diversity and Evolution* (eds Arratia, G., Schultze, H.-P. & Wilson, M. V. H.) 363–384 (Verlag Dr. Friedrich Pfeil, München, Germany, 2013).

105. Stewart, J. D. in *Mesozoic Fishes - Systematics and Paleoecology* (eds Arratia, G. & Viohl, G.) 383–394 (Verlag Dr. Friedrich Pfeil, München, Germany, 1996).

106. Murray, A. M. A new Paleocene genus and species of percopsid, *Massamorichthys wilsoni* (Paracanthopterygii) from Joffre Bridge, Alberta, Canada. *J. Vertebr. Paleontol.* **16,** 642–652 (1996).

107. Murray, A. M. & Wilson, M. V. H. in *Mesozoic Fishes 2 - Systematics and Fossil Record* (eds Arratia, G. & Schultze, H.-P.) 397–411 (Verlag Dr. Friedrich Pfeil, München, Germany, 1999).

108. Clemens, W. A. *Purgatorius* (Plesiadapiformes, Primates?, Mammalia), a Paleocene immigrant into northeastern Montana: stratigraphic occurrences and incisor proportions. *Bull. Carnegie Mus. Nat. Hist.* **122,** 3–14 (2004).

109. Clemens, W. A. in *The Hell Creek Formation and the Cretaceous-Tertiary boundary in the northern Great Plains: An integrated continental record of the end of the Cretaceous* (eds Hartman, J. H., Johnson, K. R. & Nichols, D. J.) 217–245 (Geological Society of America, Boulder, Colorado, 2002).

110. Renne, P. R. *et al.* Time scales of critical events around the Cretaceous-Paleogene boundary. *Science* **339,** 684–687 (2013).

111. Murray, A. M. & Wilson, M. V. H. A new Palaeocene genus and species of percopsiform (Teleostei: Paracanthopterygii) from the Paskapoo Formation, Smoky Tower, Alberta. *Can. J. Earth Sci.* **33,** 429–438 (1996).

112. Tyler, J. C. & Santini, F. A phylogeny of the fossil and extant zeiform-like fishes, Upper Cretaceous to Recent, with comments on the putative zeomorph clade (Acanthomorpha). *Zool. Scr.* **34,** 157–175 (2005).

113. Santini, F., Harmon, L. J., Carnevale, G. & Alfaro, M. E. Did genome duplication drive the origin of teleosts? A comparative study of diversification in ray-finned fishes. *BMC Evol. Biol.* **9,** 194 (2009).

114. Schlüter, M., Steuber, T. & Parente, M. Chronostratigraphy of Campanian–Maastrichtian platform carbonates and rudist associations of Salento (Apulia, Italy). *Cretaceous Res.* **29,** 100–114 (2008).

115. Chen, W.-J. *et al.* New insights on early evolution of spiny-rayed fishes (Teleostei: Acanthomorpha). *Front. Mar. Sci.* **1,** 53 (2014).

116. Rosenkrantz, A. Marine Upper Cretaceous and lowermost Tertiary deposits in West Greenland. *Meddelelser fra Dansk Geologisk Forening* **19,** 406–453 (1970).

117. Cohen, D. M. in *Ontogeny and Systematics of Fishes* 259–282 (The American Society of Ichthyologists and Herpetologists, 1984).

118. Eastman, J. T. & Grande, L. Late Eocene gadiform (Teleostei) skull from Seymour Island, Antarctic Peninsula. *Antarct. Sci.* **3,** 87–95 (1991).

119. Endo, H. Phylogeny of the Order Gadiformes (Teleostei, Paracanthopterygii). *Memoirs of the Graduate School of Fisheries Sciences, Hokkaido University* **49,** 75–149 (2002).

120. Kriwet, J. & Hecht, T. A review of early gadiform evolution and diversification: first record of a rattail fish skull (Gadiformes, Macrouridae) from the Eocene of Antarctica, with otoliths preserved in situ. *Naturwissenschaften* **95,** 899–907 (2008).

121. Dam, G. *et al.* Lithostratigraphy of the Cretaceous–Paleocene Nuussuaq Group, Nuussuaq Basin, West Greenland. *Geological Survey of Denmark and Greenland Bulletin* **19,** 1–171 (2009).

122. Moore, J. A. Phylogeny of the Trachichthyiformes (Teleostei: Percomorpha). *Bull. Mar. Sci.* **52,** 114–136 (1993).

123. Otero, O., Dutour, Y. & Gayet, M. *Hgulichthys*, nouveau genre de Lissoberycinae (Trachichthyiformes, Trachichthyoidea) du Cénomanien inférieur marin de Hgula (Liban). Implications phylogénétiques. *Geobios* **28,** 711–717 (1995).

124. Friedman, M. Ecomorphological selectivity among marine teleost fishes during the end-Cretaceous extinction. *Proc. Natl. Acad. Sci. USA* **106,** 5218–5223 (2009).

125. Arambourg, C. *Résultats scientifiques de la mission C. Arambourg en Syrie et en Iran (1938-1939). II. Les poissons Oligocènes de l'Iran* (Notes et Mémoires sur le Moyen-Orient, 1967).

126. Afsari, S., Yazdi, M., Bahrami, A. & Carnevale, G. A new deep-sea hatchetfish (Teleostei: Stomiiformes: Sternoptychidae) from the Eocene of Ilam, Zagros Basin, Iran. *Bollettino della Società Paleontologica Italiana* **53,** 27–37 (2014).

127. Fujii, E., Uyeno, T. & Shimaguchi, T. *Miobarbourisia aomori* gen. et sp. nov. (order Stephanoberyciformes), Miocene whalefish from Aomori, Japan. *Bulletin of the National Museum of Nature and Science. Series C, Geology & Paleontology* **33,** 89–93 (2007).

128. Aoyagi, K. & Omokawa, M. Diagenesis of Neogene diatoms and their importance as a source of petroleum in Japan. *The Island Arc* **2,** 273–279 (1993).

129. Ebeling, A. W. *Scopelogadus* (?) *capistranensis*, a new fossil melamphaid (Pisces: Teleostei) from Capistrano Beach, California. *Postilla* **71,** 1–6 (1962).

130. Corsi, A., Landini, W. & Sorbini, C. A new ichthyofauna from the Upper Miocene of Ca' Matterella (Ravenna, Italy); paleoecological and paleobiogeographical considerations. *Studi e Ricerche sui Giaciamenti Terziari di Bolca* **8,** 56–76 (1999).

131. Bonde, N. Osteoglossomorphs of the marine Lower Eocene of Denmark – with remarks on other Eocene taxa and their importance for palaeobiogeography. *Geological Society, London, Special Publications* **295,** 253–310 (2008).

132. Patterson, C. An overview of the early fossil record of acanthomorphs. *Bull. Mar. Sci.* **52,** 29–59 (1993).

133. Tyler, J. C., Jerzmanska, A., Bannikov, A. F. & Swidnicki, J. Two new genera and species of Oligocene spikefishes (Tetraodontiformes: Triacanthodidae), the first fossils of the Hollardiinae and Triacanthodinae. *Smithson. Contr. Paleobiol.* **75,** 1–27 (1993).

134. Alfaro, M. E., Santini, F. & Brock, C. D. Do reefs drive diversification in marine teleosts? Evidence from the pufferfishes and their allies (order Tetraodontiformes). *Evolution* **61,** 2104–2126 (2007).

135. Santini, F. *et al.* Do habitat shifts drive diversification in teleost fishes? An example from the pufferfishes (Tetraodontidae). *J. Evol. Biol.* **26,** 1003–1018 (2013).

136. Carnevale, G. & Johnson, G. D. A Cretaceous cusk-eel (Teleostei, Ophidiiformes) from Italy and the Mesozoic diversification of percomorph fishes. *Copeia* **103,** 771–791 (2015).

137. Přikryl, T. & Carnevale, G. Ophidiiform fishes from the Oligocene–early Miocene of Moravia, Czech Republic. *Bull. Geosci.* **93,** 477–489 (2018).

138. Dalla Vecchia, F. M. I dinosauri del Villaggio del Pescatore (Trieste): qualche aggiornamento. *Atti del Museo Civico di Storia Naturale di Trieste* **Suppl. al 53,** 111–130 (2008).

139. Přikryl, T. & Carnevale, G. An Oligocene toadfish (Teleostei, Percomorpha) from Moravia, Czech Republic: The earliest skeletal record for the order Batrachoidiformes. *Bull. Geosci.* **92,** 123–131 (2017).

140. Gregorová, R., Micklich, N. & Přikryl, T. First record of the family Moronidae (Perciformes) in the Menilitic Formation of the Litenčice locality (Oligocene, Rupelian; Moravia, Czech Republic). *N. Jb. Geol. Pal. A* **280,** 79–86 (2016).

141. Bannikov, A. F. & Carnevale, G. †*Carlomonnius quasigobius* gen. et sp. nov.: the first gobioid fish from the Eocene of Monte Bolca, Italy. *Bull. Geosci.* **91,** 13–22 (2016).

142. Monsch, K. A. A revision of scombrid fishes (Scombroidei, Perciformes) from the Middle Eocene of Monte Bolca, Italy. *Palaeontology* **49,** 873–888 (2006).

143. Trevisani, E., Papazzoni, C. A., Ragazzi, E. & Roghi, G. Early Eocene amber from the "Pesciara di Bolca" (Lessini Mountains, Northern Italy). *Palaeogeogr. Palaeoclimatol. Palaeoecol.* **223,** 260–274 (2005).

144. Žalohar, J., Hitij, T. & Križnar, M. Two new species of seahorses (Syngnathidae, Hippocampus) from the Middle Miocene (Sarmatian) Coprolitic Horizon in Tunjice Hills, Slovenia: The oldest fossil record of seahorses. *Annales de Paléontologie* **95,** 71–96 (2009).

145. Wilson, A. B. & Orr, J. W. The evolutionary origins of Syngnathidae: pipefishes and seahorses. *J. Fish Biol.* **78,** 1603–1623 (2011).

146. Harzhauser, M. & Piller, W. E. Integrated stratigraphy of the Sarmatian (Upper Middle Miocene) in the western Central Paratethys. *Stratigraphy* **1,** 65–86 (2004).

147. Vasiliev, I. *et al.* The age of the Sarmatian–Pannonian transition in the Transylvanian Basin (Central Paratethys). *Palaeogeogr. Palaeoclimatol. Palaeoecol.* **297,** 54–69 (2010).

148. Carnevale, G. & Pietsch, T. W. An Eocene frogfish from Monte Bolca, Italy: The earliest known skeletal record for the family. *Palaeontology* **52,** 745–752 (2009).

149. Pietsch, T. W. & Carnevale, G. A new genus and species of anglerfish (Teleostei: Lophiiformes: Lophiidae) from the Eocene of Monte Bolca, Italy. *Copeia* **2011,** 64–71 (2011).

150. Carnevale, G. & Pietsch, T. W. Batfishes from the Eocene of Monte Bolca. *Geolog. Mag.* **148,** 461–472 (2011).

151. Carnevale, G. & Pietsch, T. W. †*Caruso*, a new genus of anglerfishes from the Eocene of Monte Bolca, Italy, with a comparative osteology and phylogeny of the teleost family Lophiidae. *J. Syst. Palaeontol.* **10,** 47–72 (2012).

152. Martini, E., Ott, W. & Keller, M. Der Grenzbereich Untere/Obere Hydrobien-Schichten - ein neuer Aufschluss in Frankfurt am Main: Nannoplankton und Fisch-Skelette (*Morone aequalis*). *Bericht des Offenbacher Vereins für Naturkunde* **104,** 2–18 (2004).

153. Martini, E. & Schiller, W. Contribution to the biodiversity of the lower Frankfurt Formation (Miocene) in the Hanau Basin (Germany): a new *Hymenomonas* (calcareous nannoplankton) and a new *Scrippsiella* (dinoflagellate) species. *Palaeobio. Palaeoenv.* **97,** 687–701 (2017).

154. Reichenbacher, B., Gaudant, J. & Griessemer, T. W. A late Burdigalian gobiid fish, *Gobius brevis* (Agassiz, 1839), in the Upper Hydrobia Beds in the middle Upper Rhine Graben (W-Germany). *Paläontologische Zeitschrift* **81,** 365–375 (2007).

155. Roe, L. J. Phylogenetic and ecologial significance of Channidae (Osteichthyes, Teleostei) from the Early Eocene Kuldana Formation of Kohat, Pakistan. *Contributions from the Museum of Paleontology* **28,** 93–100 (1991).

156. Murray, A. M. & Thewissen, J. G. M. Eocene actinopterygian fishes from Pakistan with the description of a new genus and species of channid channiformes. *J. Vertebr. Paleontol.* **28,** 41–52 (2008).

157. Gingerich, P. D. Stratigraphic and micropaleontological constraints on the middle Eocene age of the mammal-bearing Kuldana Formation of Pakistan. *J. Vertebr. Paleontol.* **23,** 643–651 (2003).

158. Sanders, M. Die fossilen Fische der alttertiären Süsswasserablagerungen aus Mittel-Sumatra. *Verhandelingen van het Geologisch-Mijnbouwkundig Genootschap voor Nederland en Koloni'n. Geologische Series* **11,** 1–144 (1934).

159. Murray, A. M. *et al.* A fossil gourami (Teleostei, Anabantoidei) from probable Eocene deposits of the Ombilin Basin, Sumatra, Indonesia. *J. Vertebr. Paleontol.* **35,** e906444 (2015).

160. Altner, M. & Reichenbacher, B. †Kenyaichthyidae fam. nov. and †*Kenyaichthys* gen. nov. – First record of a fossil aplocheiloid killifish (Teleostei, Cyprinodontiformes). *PLoS ONE* **10,** e0123056 (2015).

161. Hill, A., Curtis, G. & Drake, R. Sedimentary stratigraphy of the Tugen Hills, Baringo, Kenya. *Geological Society, London, Special Publications* **25,** 285–295 (1986).

162. Sawada, Y. *et al.* The age of *Orrorin tugenensis*, an early hominid from the Tugen Hills, Kenya. *C. R. Palevol.* **1,** 293–303 (2002).

163. Cione, A. L. Los perces continentales del Cenozoico de Argentina. *Actas del Congreso Argentino de Paleontologia y Biostratigrafia* **4,** 101–106 (Oct. 1986).

164. Del Papa, C. E. Sedimentation on a ramp type lake margin: Paleocene-Eocene Maıéz Gordo Formation, northwestern Argentina. *J. S. Am. Earth Sci.* **12,** 389–400 (1999).

165. Vucetich, M. G. *et al.* Mamıéferos continentales del Paleógeno argentino: las investigaciones de los últimos cincuenta años. *Ameghiniana* **Publicación Especial 11,** 239–255 (2007).

166. Cione, A. L. & Báez, A. M. Peces continentales y anfibios cenozoicos de Argentina: los últimos cincuenta años. *Ameghiniana* **Publicación Especial 11,** 195–220 (2007).

167. López-Fernández, H. & Albert, J. S. in *Historical Biogeography of Neotropical Freshwater Fishes* (eds Albert, J. S. & Reis, R. E.) 105–118 (University of California Press, London, United Kingdom, 2011).

168. Gaudant, J. Les cyprinodontiformes (poissons téléostéens) oligocènes de Ronzon, Le Puy-en-Velay (Haute-Loire): Anatomie et signification paléoécologique. *Geobios* **21,** 773–785 (1988).

169. Gaudant, J. Découverte d'une nouvelle espèce de poissons cyprinodontiformes (*Prolebias delphinensis* nov. sp.) dans l'Oligocène du bassin de Montbrun-les-Bains (Drôme). *Géologie Méditerranéene* **XVI,** 355–367 (1989).

170. Costa, W. J. E. M. The caudal skeleton of extant and fossil cyprinodontiform fishes (Teleostei: Atherinomorpha): comparative morphology and delimitation of phylogenetic characters. *Vertebr. Zool.* **62,** 161–180 (2012).

171. Smith, W. L., Chakrabarty, P. & Sparks, J. S. Phylogeny, taxonomy, and evolution of Neotropical cichlids (Teleostei: Cichlidae: Cichlinae). *Cladistics* **24,** 625–641 (2008).

172. Perez, P. A., Malabarba, M. C. & Del Papa, C. A new genus and species of Heroini (Perciformes: Cichlidae) from the early Eocene of southern South America. *Neotrop. Ichthyol.* **8,** 631–642 (2010).

173. Malabarba, M. C., Malabarba, L. R. & Del Papa, C. *Gymnogeophagus eocenicus*, n. sp (Perciformes: Cichlidae), an Eocene cichlid from the Lumbrera formation in Argentina. *J. Vertebr. Paleontol.* **30,** 341–350 (2010).

174. Del Papa, C. *et al.* Sedimentological, geochemical and paleontological insights applied to continental omission surfaces: A new approach for reconstructing an Eocene foreland basin in NW Argentina. *J. S. Am. Earth Sci.* **29,** 327–345 (2010).

175. Matschiner, M. Gondwanan vicariance or trans-Atlantic dispersal of cichlid fishes: a review of the molecular evidence. *Hydrobiologia* **832,** 9–37 (2019).

176. López-Fernández, H., Winemiller, K. O. & Honeycutt, R. L. Multilocus phylogeny and rapid radiations in Neotropical cichlid fishes (Perciformes: Cichlidae: Cichlinae). *Mol. Phylogenet. Evol.* **55,** 1070–1086 (2010).

177. Ilves, K. L., Torti, D. & López-Fernández, H. Exon-based phylogenomics strengthens the phylogeny of Neotropical cichlids and identifies remaining conflicting clades (Cichliformes: Cichlidae: Cichlinae). *Mol. Phylogenet. Evol.* **118,** 232–243 (2018).

178. Bardack, D. New Tertiary teleosts from Argentina. *Am. Mus. Novit.* **2041,** 1–28 (1961).

179. Malabarba, M. C. & Malabarba, L. R. A new cichlid *Tremembichthys garcia* (Actinopterygii, Perciformes) from the Eocene-Oligocene of Eastern Brazil. *Rev. Bras. Paleontolog.* **11,** 59–68 (2008).

180. Garcia, M. J., Santos, M. & Hasui, Y. Palinologia da parte aflorante da Formação Entre-Córregos, Bacia de Aiuruoca, Terciário do Estado de Minas Gerais, Brasil. *Revista Universidade de Guarulhos - Geociências* **5,** 259 (2000).

181. Weiss, F. E. *Characiformes fósseis (Teleostei: Ostariophysi) da formação Entre-Córregos, Bacia Terciária de Aiuruoca, Minas Gerais* PhD thesis (Instituto de Biociências da Universidade Federal do Rio Grande do Sul, Porto Alegre, 2008).

182. Murray, A. M. Eocene cichlid fishes from Tanzania, East Africa. *J. Vertebr. Paleontol.* **20,** 651–664 (2001).

183. Harrison, T. *et al.* in *Eocene Diversity: Unusual Occurrences and Rarely Sampled Habitats* (ed Gunnell, G. F.) 39–74 (Kluwer Academic/Plenum Publishers, New York, 2001).

184. Lippitsch, E. & Micklich, N. Cichlid fish biodiversity in an Oligocene lake. *Ital. J. Zool.* **65,** 185–188 (1998).

185. Murray, A. M. The fossil record and biogeography of the Cichlidae (Actinopterygii: Labroidei). *Biol. J. Linn. Soc.* **74,** 517–532 (2001).

186. Brown, G. F., Schmidt, D. L. & Huffman Jr, A. C. Geology of the Arabian Peninsula. *US Geological Survey Professional Paper* **560 A** (1989).

187. Schwarzer, J., Misof, B., Tautz, D. & Schliewen, U. K. The root of the East African cichlid radiations. *BMC Evol. Biol.* **9,** 186 (2009).

188. Irisarri, I. *et al.* Phylogenomics uncovers early hybridization and adaptive loci shaping the radiation of Lake Tanganyika cichlid fishes. *Nat. Commun.* **9,** 3159 (2018).

189. Stiassny, M. L. J. in *Cichlid Fishes - Behaviour, Ecology and Evolution* (ed Keenleyside, M. H. A.) 1–35 (Chapman & Hall, London, United Kingdom, 1991).

190. Murray, A. M. *The Eocene cichlids (Perciformes: Labroidei) of Mahenge, Tanzania* PhD thesis (McGill University, Montreal, 2000).

191. Murray, A. M. The oldest fossil cichlids (Teleostei: Perciformes): indication of a 45 million-year-old species flock. *Proc. R. Soc. B* **268,** 679–684 (2001).

192. Schwarzer, J., Lamboj, A., Langen, K., Misof, B. & Schliewen, U. K. Phylogeny and age of chromidotilapiine cichlids (Teleostei: Cichlidae). *Hydrobiologia* **748,** 185–199 (2015).

193. Schliewen, U. K. & Stiassny, M. L. J. *Etia nguti*, a new genus and species of cichlid fish from the River Mamfue, Upper Cross River basin in Cameroon, West-Central Africa. *Ichthyol. Explor. Freshwaters* **14,** 61–71 (2003).

194. Penk, S. B. R., Altner, M., Cerwenka, A., Schliewen, U. K. & Reichenbacher, B. New fossil cichlid from the middle Miocene of east Africa revealed as oldest known member of the Oreochromini. *Sci. Rep.* **9,** 10198 (2019).

195. Rasmussen, C. *et al.* Middle–late Miocene palaeoenvironments, palynological data and a fossil fish Lagerstätte from the Central Kenya Rift (East Africa). *Geolog. Mag.* **154,** 24–56 (2017).

196. Genner, M. J. *et al.* Age of cichlids: new dates for ancient lake fish radiations. *Mol. Biol. Evol.* **24,** 1269–1282 (2007).

197. Azuma, Y., Kumazawa, Y., Miya, M., Mabuchi, K. & Nishida, M. Mitogenomic evaluation of the historical biogeography of cichlids toward reliable dating of teleostean divergences. *BMC Evol. Biol.* **8,** 215 (2008).

198. Matschiner, M., Hanel, R. & Salzburger, W. On the origin and trigger of the notothenioid adaptive radiation. *PLOS ONE* **6,** e18911 (2011).

199. McMahan, C. D., Chakrabarty, P., Sparks, J. S., Smith, W. L. & Davis, M. P. Temporal patterns of diversification across global cichlid biodiversity (Acanthomorpha: Cichlidae). *PLoS ONE* **8,** e71162 (2013).

200. Near, T. J. *et al.* Phylogeny and tempo of diversification in the superradiation of spiny-rayed fishes. *Proc. Natl. Acad. Sci. USA* **110,** 12738–12743 (2013).

201. Betancur-R, R. *et al.* The Tree of Life and a new classification of bony fishes. *PLoS Currents: Tree of Life,* doi:10.1371/currents.tol.53ba26640df0ccaee75bb165c8c26288 (2013).

202. Roth, O. *et al.* Evolution of male pregnancy associated with remodeling of canonical vertebrate immunity in seahorses and pipefishes. *Proc. Natl. Acad. Sci. USA* **117,** 9431–9439 (2020).
